# Identification of a Dual Inhibitor of Secreted Phospholipase A_2_ (GIIA sPLA_2_) and SARS-CoV-2 Main Protease

**DOI:** 10.3390/ph15080961

**Published:** 2022-08-03

**Authors:** Maria A. Theodoropoulou, Giorgos S. Koutoulogenis, Linlin Zhang, Ifigeneia Akrani, Emmanuel Mikros, Rolf Hilgenfeld, George Kokotos

**Affiliations:** 1 Department of Chemistry, National and Kapodistrian University of Athens, Panepistimiopolis, 15771 Athens, Greece; martheod@chem.uoa.gr (M.A.T.); gkoutoul@chem.uoa.gr (G.S.K.); 2 Center of Excellence for Drug Design and Discovery, National and Kapodistrian University of Athens, 15771 Athens, Greece; 3 Institute of Molecular Medicine, University of Lübeck, Ratzeburger Allee 160, 23562 Lübeck, Germany; llzhang@biochem.uni-luebeck.de (L.Z.); rolf.hilgenfeld@uni-luebeck.de (R.H.); 4 Department of Pharmacy, National and Kapodistrian University of Athens, Panepistimiopolis, 15771 Athens, Greece; iakrani@pharm.uoa.gr (I.A.); mikros@pharm.uoa.gr (E.M.); 5 Athena Research and Innovation Center in Information Communication & Knowledge Technologies, 15125 Marousi, Greece; 6 German Center for Infection Research (DZIF), Hamburg–Lübeck–Borstel–Riems Site, University of Lübeck, 23562 Lübeck, Germany

**Keywords:** COVID-19, inhibitors, main protease, 2-oxoamides, phospholipase A_2_, SARS-CoV-2

## Abstract

The development of novel agents to combat COVID-19 is of high importance. SARS-CoV-2 main protease (M^pro^) is a highly attractive target for the development of novel antivirals and a variety of inhibitors have already been developed. Accumulating evidence on the pathobiology of COVID-19 has shown that lipids and lipid metabolizing enzymes are critically involved in the severity of the infection. The purpose of the present study was to identify an inhibitor able to simultaneously inhibit both SARS-CoV-2 M^pro^ and phospholipase A_2_ (PLA_2_), an enzyme which plays a significant role in inflammatory diseases. Evaluating several PLA_2_ inhibitors, we demonstrate that the previously known potent inhibitor of Group IIA secretory PLA_2_, GK241, may also weakly inhibit SARS-CoV-2 M^pro^. Molecular mechanics docking and molecular dynamics calculations shed light on the interactions between GK241 and SARS-CoV-2 M^pro^. 2-Oxoamide GK241 may represent a lead molecular structure for the development of dual PLA_2_ and SARS-CoV-2 M^pro^ inhibitors.

## 1. Introduction

With more than 450 million cases of infected people and 6 million casualties globally, the discovery of efficient agents to treat COVID-19, which is caused by the severe acute respiratory syndrome coronavirus-2 (SARS-CoV-2), is an unmet need [1]. Pioneering studies showed that enzymes, such as RNA-dependent RNA polymerase (RdRp) and SARS-CoV-2 main protease (M^pro^), are attractive targets for the development of novel antiviral agents [2,3].

Studies aiming to understand the pathobiology of COVID-19 have also demonstrated the involvement of lipids and lipid metabolizing enzymes in this potentially lethal infection [4,5]. Phospholipases A_2_ (PLA_2_s) are enzymes which catalyze the hydrolysis of membrane glycerophospholipids, releasing free fatty acids (FFAs) and lysophospholipids and initiating arachidonic acid (AA) cascade and promotion of inflammation [6,7,8]. Proteomics studies on SARS-CoV-2 infected cells have revealed alterations of proteins linked to the inflammatory response due to the viral infection [9]. The expression of two PLA_2_s, namely cytosolic PLA_2_ (GIVA cPLA_2_) and secreted PLA_2_ (GIIA sPLA_2_) was notably differentiated after 24 h of infection [9]. Plasma metabolomic and lipidomic studies associated with COVID-19 showed elevated levels of FFAs and reduction in phosphatidylcholines (PCs), which indicated increased enzymatic activity of PLA_2_s [10]. Large-scale plasma analysis has revealed that lipids are strongly involved in the response to infection [11]. The concentrations of oleic acid (OA, C18:1) and AA (C20:4) were directly correlated to the severity of the disease in COVID-19 patients who required admission to an intensive care unit [11]. Most recently, an independent cohort study has demonstrated elevated levels of GIIA sPLA_2_ in the plasma of deceased patients in comparison to patients with severe or mild COVID-19, indicating that GIIA sPLA_2_ is associated with increased mortality due to COVID-19 [12]. This study highlights the importance of GIIA sPLA_2_, establishing this enzyme as a factor that leads to severe COVID-19 morbidity and mortality, and suggesting it as a therapeutic target to prevent COVID-19 mortality.

Previously described data [4,5,9,10,11,12], as well as data reported in recent review articles summarizing the role of PLA_2_s in inflammatory diseases [13,14], prompted us to explore if we could identify a small-molecule inhibitor able to simultaneously inhibit PLA_2_ and SARS-CoV-2 M^pro^. It would be advantageous, if we could target two enzymes with a dual inhibitor able to simultaneously block virus replication by inhibiting SARS-CoV-2 M^pro^ and regulate the inflammatory response by inhibiting PLA_2_. In the present study, we focus on 2-oxoamide (also known as α-ketoamide) small molecules as appropriate agents to inhibit both PLA_2_ and SARS-CoV-2 M^pro^ and we describe the first dual inhibitor of GIIA sPLA_2_ and SARS-CoV-2 M^pro^.

## 2. Results

### 2.1. Design of Inhibitors

**Figure 1:**
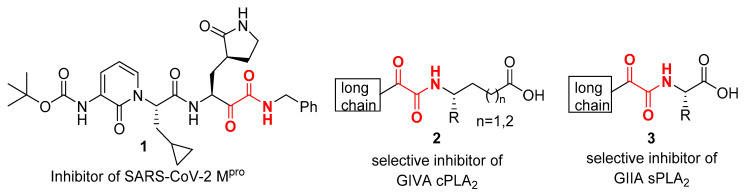
Structures of 2-oxoamide inhibitor of SARS-CoV-2 M^pro^ and 2-oxoamide inhibitors of GIVA cPLA_2_ and GIIA sPLA_2_.

In 2020, Hilgenfeld and coworkers demonstrated that 2-oxoamides are potent inhibitors of SARS-CoV-2 M^pro^ [15] and reported the X-ray structure of SARS-CoV-2 M^pro^ in complex with the 2-oxoamide inhibitor **1** (Figure 1) [15]. SARS-CoV-2 M^pro^ is a cysteine protease and its key cysteine residue may attack small-molecule inhibitors containing either a reactive carbonyl group or a Michael acceptor functionality [15,16,17,18,19,20,21]. In previous years, we have designed and synthesized a variety of 2-oxoamides as inhibitors of PLA_2_s. More specifically, we have developed 2-oxoamides (**2**), which are based on non-natural δ- or γ-amino acids and selectively inhibit GIVA cPLA_2_ [22,23], while 2-oxoamides (**3**) based on natural α-amino acids selectively inhibit GIIA sPLA_2_ [24].

To explore if known inhibitors of PLA_2_s may inhibit SARS-CoV-2 M^pro^, we selected three 2-oxoamides previously developed by us, which are selective inhibitors of either GIVA cPLA_2_ (AX109 and AX074) [22,23] or GIIA sPLA_2_ (GK241) [24], and pentafluoroethyl ketone GK187, which selectively inhibits calcium-independent GVIA iPLA_2_ [25]. Their structures are shown in Table 1. Since our initial in vitro studies have shown that GIIA sPLA_2_ inhibitor GK241 inhibits SARS-CoV-2 M^pro^, we have also synthesized several GK241 analogs for SAR studies.

### 2.2. Synthesis

**Scheme 1:**
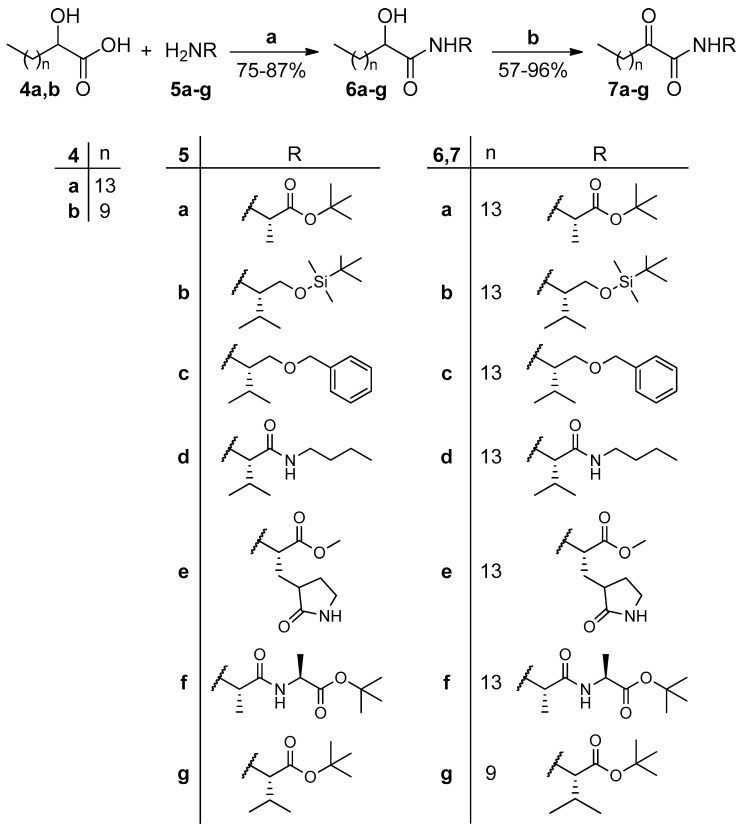
Synthesis of 2-oxoamides from 2-hydroxycarboxylic acids **4a,b** and amines **5a**–**g**. (a) EDC∙HCl, HOBt, Et_3_N, dry CH_2_Cl_2_, 1 h at 0 °C, 16 h at r.t.; (b) Dess–Martin periodinane, dry CH_2_Cl_2_, 1 h, r.t.

2-Oxoamides **7a**–**g** were synthesized in two steps from long chain α-hydroxycarboxylic acids **4a,b** and amines **5a**–**g** (see Scheme 1 below). The coupling was carried out employing 1-ethyl-3-(3-dimethylaminopropyl)carbodiimide hydrochloride (EDC∙HCl) as the coupling agent in the presence of 1-hydroxybenzotriazole (HOBt) and was followed by an oxidation reaction using Dess–Martin periodinane (Scheme 1) [26].

**Scheme 2:**
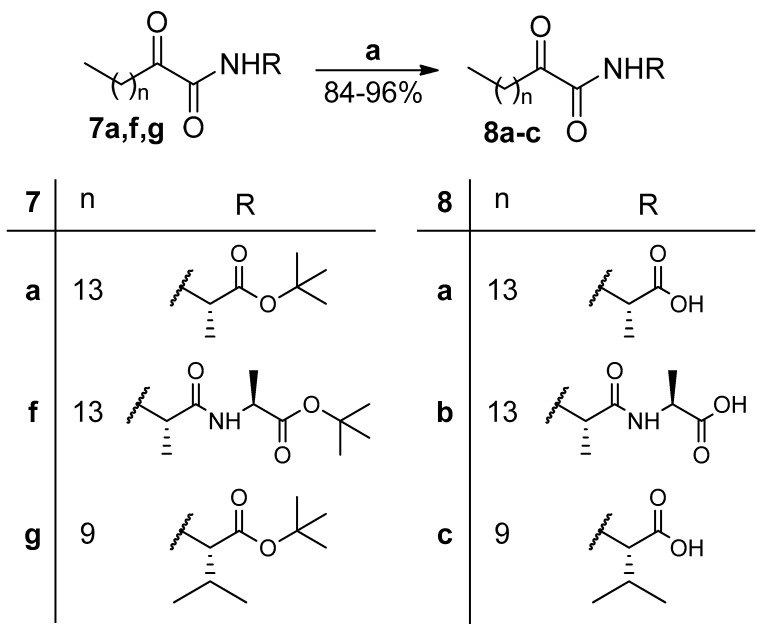
Removal of *tert*-butyl ester group. (a) 50% TFA in CH_2_Cl_2_ (0.5 M), 3 h, r.t.

*tert*-Butyl esters **7a** and **7f**,**g** were deprotected by treatment with trifluoroacetic acid (TFA) to provide the corresponding carboxylic acids **8a**–**c** (Scheme 2).

**Scheme 3:**
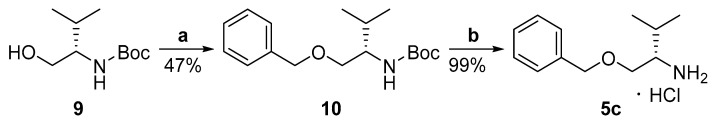
Synthesis of amine **5c**. (a) BnBr, NaH, dry DMF, 16 h, 0 °C to r.t.; (b) 4N HCl/MeOH, 2 h, r.t.

Amine **5b** was synthesized from carbobenzoxy-L-valinol by protection of the hydroxyl group, using *tert*-butyldimethylsilyl chloride, as described in [27], and then removal of the Cbz group. Amine **5c** was synthesized from *tert*-butyloxycarbonyl-L-valinol (**9**) by protection of the hydroxyl group, followed by removal of the Boc group (Scheme 3).

### 2.3. Inhibition of SARS-CoV-2 M^pro^ by 2-Oxoamide PLA_2_ Inhibitors and Analogs

The inhibitory potency of the known PLA_2_ inhibitors and all of the new 2-oxoamides synthesized against SARS-CoV-2 M^pro^ was assessed by determining the extent of enzyme inhibition (% inhibition); the results are summarized in Table 1 and Table 2. In these experiments, 40 μM or 100 μM of 2-oxoamide, 0.5 μM of SARS-CoV-2 M^pro^, and 10 μM of the fluorescence resonance energy transfer (FRET) substrate Dabcyl-KTSAVLQ↓SGFRKM-E(Edans)-NH_2_ in 20 mM tris(hydroxymethyl)aminomethane (Tris) buffer (pH 7.3), 100 mM NaCl, 1 mM ethylene diamine tetra-acetic acid (EDTA), were used.

As shown in Table 1, 2-oxoamide GIVA cPLA_2_ inhibitors AX109 and AX074 weakly inhibited SARS-CoV-2 M^pro^ at concentrations of 40 μM and 100 μM (entries 1 and 2, Table 1), but none of them higher than 50% even at 100 μM. Interestingly, the 2-oxoamide GIIA sPLA_2_ inhibitor GK241 inhibited SARS-CoV-2 M^pro^ almost completely at 100 μM and by 76.9% at 40 μM (entry 3, Table 1). Pentafluoroethyl ketone GK187, which is a selective and potent GVIA iPLA_2_ inhibitor, showed a very weak effect even at 100 μM. Overall, the selective GIIA sPLA_2_ inhibitor GK241 was found to inhibit SARS-CoV-2 M^pro^_._ The IC_50_ value for GK241, determined by the inhibition curve, was found to be 24 μM.

The results for the in vitro inhibition of SARS-CoV-2 M^pro^ by the analogs of GK241 are summarized in Table 2. Conversion of the free carboxyl to the corresponding amide **7d** or to a hydroxymethyl-protected group **7c**, **7b** resulted in abolishment of the inhibitory potency (entries 2–4, Table 2), indicating that a free carboxyl group was necessary for the inhibition. When the long chain of GK241 was replaced by a shorter one (reduction by four carbon atoms), the inhibitory potency of **8d** on SARS-CoV-2 M^pro^ was abolished (entry 9, Table 2).

When the valine residue of GK241 was replaced by alanine, **8a** (entry 7, Table 2) was found to inhibit SARS-CoV-2 M^pro^ by 65.49% at 40 μM, while the conversion of the free carboxyl group to an ester (**7a**, entry 1, Table 2) again led to the abolishment of the inhibitory potency. The derivatives **8b** and **7f**, based on an Ala-Ala dipeptide, presented almost no activity (entries 8 and 6, Table 2). Finally, compound **7e**, based on a glutamine surrogate, did not present any activity (entry 5, Table 2).

### 2.4. Molecular Mechanics Docking and Molecular Dynamics Calculations

**Figure 2:**
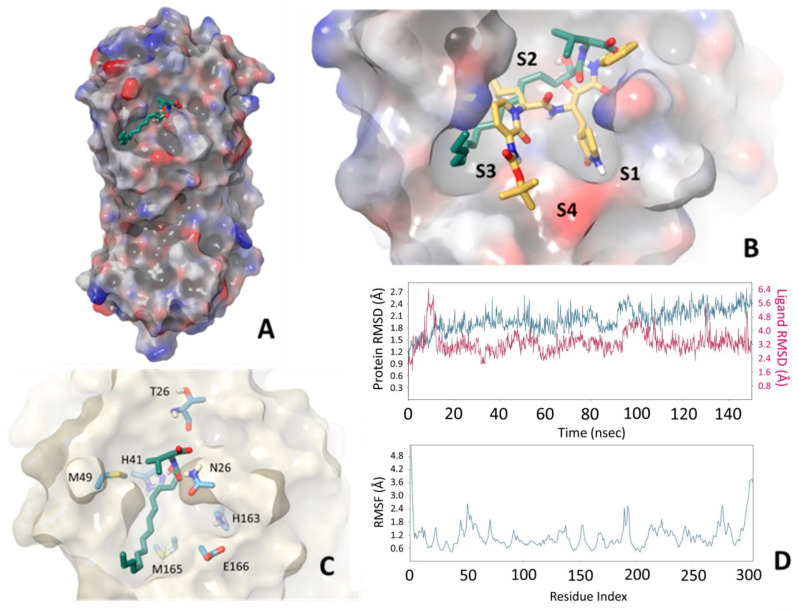
Structural characteristics of SARS-CoV-2 M^pro^-GK241 interaction as provided by docking and MD simulations. (**A**) GK241 occupying the main cavity of the enzyme active site. (**B**) Comparison of structure 7 with crystal structure PDB 6Y2F. The formation of the covalent bond produces exactly the same configuration, and the amide bond has similar orientation, while the long aliphatic chain occupies part of the S2 and S3 and S4 cavities. (**C**) Interactions of structure 7 with different protein residues. (**D**) RMSD for the protein (blue) and the ligand (magenta), as well as protein residue RMSF during the 150 nsec MD simulation.

To obtain a better insight into the interactions between SARS-CoV-2 M**^pro^** and the most active compound GK241, we applied molecular mechanics docking and molecular dynamics (MD) calculations. More specifically, to simulate the specific interaction, compound GK241 was subjected to covalent docking calculations as implemented in the Maestro Schroedinger suite by creating a covalent bond between Cys145 and the 2-carbonyl carbon of the 2-oxoamide moiety. The procedure transformed carbonyl to an *sp^3^* carbon atom and the adjacent oxygen to a hydroxyl group. The crystal structure of SARS-CoV-2 M^pro^ protein PDB 6Y2F [15] was adequately prepared for simulations by adding the two missing flexible residues E47 and D48, as this loop was near the binding cavity and initial calculations showed that it could interact with the ligand. Moreover, initial calculations showed that the formation of the covalent bond could result in both *R* and *S* configurations for the 2-carbon atom, which is not surprising, since there are examples in the literature reporting inhibitors that may lead to both *R* and *S* configurations [28]. Thus, docking calculations were finally performed to collect 100 structures for each 2-carbon atom configuration. These structures were further ranked according to the ligand binding energy calculated using the MM-GBSA approach, ranging between −58.69 to −10.66 kcal/mol. Common structural characteristic of the generated structures for both the *R* and *S* configuration groups was the tendency to orient the long aliphatic chain to S2, S3, S4 protease clefts (Figure 2A,B), while differences were mainly observed in the conformation of the covalent bond formatted between Cys145 and the 2-oxoamide carbonyl, as expressed by the dihedral angle Cβ**_cys145_**-S**_cys145_**-C2-O2. The lowest energy structures of up to 3 kcal relative binding energy were grouped according to common conformational characteristics of the ligand; representative structures are shown in Figure 2C and Figure S1 (Supplementary Materials). The resulting docking structures appeared to sample the available conformational space by orienting the valine moiety carboxylate (structures 3 and 5) or isopropyl (structures 2 and 4) to the S1 cavity, while in structures 1 and 6 the –OH group or the initial part of the aliphatic chain were oriented in this cleft. Another interesting structural characteristic was that only structures 2, 5 and 7 interacted with H41 specifically through the –OH group. These seven selected structures were further subjected to 50 nsec MD calculations to assess the stability of the protein-ligand interaction. The calculated binding energies and mean RMSD for both protein and ligand are summarized in Table S1 (Supplementary Materials) along with major interactions between the ligand and specific protein residues. Visual inspection of the MD trajectories and conformational flexibility of both the protein and the ligand showed that, in most of the structures, the long aliphatic chain had the tendency to widely explore the conformational space, probably inducing protein conformational changes. The RMSF calculated for the protein Cα showed that the loop between residues 45–50, in particular, exhibited increased flexibility (Figure S2, Supplementary Materials) which appeared to be related to interactions with part of the long aliphatic chain. A relative flexibility was also observed for residues 185–192, specifically in the case of structure 1. Among the seven structures, the most stable during MD simulations was found to be structure 7, and, thus, MD simulations were extended to 150 nsec to validate the initial observation (Figure 2D). In structure 7, the 2-carbon atom exhibited an *S* configuration and both the –OH and amide moieties aligned, in general, very well with the ketoamide crystal structure reported by Hilgenfeld and coworkers [15] (Figure 2A,B). The –OH group interacted with H41 for 50% of the MD sampled structures, while the valine side-chain occupied the S1′ cavity with the carboxylate interacting with Q166 and the amide NH forming an H-bond with the T26 backbone carbonyl during the whole MD simulation, both directly and through a water molecule. Both protein and ligand RMSD appeared to be low during the whole MD simulation and the protein RMSF validated the stability of all parts of the protein (Figure 2C,D).

In order to further rationalize the experimental results, the derivatives **8d** and **8a** were subjected to covalent docking following the same procedure as above, resulting in 100 structures for each of the *R* and *S* 2-carbon configurations. The calculated MM-GBSA interaction energies were higher in both cases than those observed for GK241, ranging between −50.91 and −8.35 kcal/mol for **8d** and −56.99 and −25.98 kcal/mol for **8a**. For comparison with GK241, we selected structures, similar to structure 7, specifically having an *S* configuration and forming an H bond between the –OH and residue H41, which are presented in Figure S3 (Supplementary Materials) along with the crystal structure and structure 7 for comparison. Concerning the derivative **8d,** a major difference observed was that the 10-carbon-atom aliphatic chain was always oriented differently covering part of the S1 and S2 cavities, as shown in Figure S3A (Supplementary Materials). This major difference in the conformation of the aliphatic side-chain reflected a major difference in the MM-GBSA calculated binding energy of ~4 kcal compared to GK241 structure 7 and was in agreement with the experimental results showing no activity at 40 μΜ concentration. On the other hand, derivative **8a** showed major similarities with GK241 as far as the aliphatic chain was concerned, mainly occupying the same part of the enzyme active site. However, the alanine moiety of **8a** occupied the S1 cavity differently to GK241 as the N-Cα**_ala_** bond adopted a different conformation compared to GK241, resulting in a different orientation of the methyl group of the alanine moiety compared to the corresponding valine isopropyl. These differences can explain the small differences observed in the experimental activity of these derivatives.

## 3. Discussion

PLA_2_s are a superfamily of enzymes [6,7,8] which are involved in almost any inflammatory disease [6,7,8,13,14,29]. In humans, three PLA_2_ types, represented by GIIA sPLA_2_, GIVA cPLA_2_ and GVIA iPLA_2_, are of high medicinal interest and have been targets for the development of small-molecule synthetic inhibitors [13]. Among the various classes of synthetic inhibitors, 2-oxoamides constitute a class of compounds whose members can selectively inhibit either GIVA cPLA_2_ or GIIA sPLA_2_. The results of the present study showed that the selective 2-oxamide inhibitors of GIVA cPLA_2_ AX109 and AX074 [22,23] did not exhibit any appreciable inhibition of SARS-CoV-2 M^pro^. Similarly, the selective pentafluoroethyl ketone inhibitor of GVIA iPLA_2_ GK187 [25] did not show appreciable inhibition of SARS-CoV-2 M^pro^. On the contrary, the potent 2-oxamide inhibitor of GIIA sPLA_2_ (IC_50_ 143 nM) [24] was found to inhibit SARS-CoV-2 M^pro^ with an IC_50_ value of 24 μM. Given that GIIA sPLA_2_ has most recently been recognized as a factor contributing to the severity and mortality of COVID-19 [12,30], this finding is of high importance.

Peptide and peptide-mimetic 2-oxoamides have been identified as potent inhibitors of SARS-CoV-2 M^pro^ and their interaction with the catalytic site of the cysteine protease SARS-CoV-2 M^pro^ has been defined by determining the X-ray structure of the enzyme-inhibitor complex [15]. Inhibitors of SARS-CoV-2 M^pro^ have attracted high interest as candidate antiviral drugs [31,32], and, recently, the inhibitor PF-07321332 (nirmatrelvir) has received emergency approval by the Food and Drug Administration (FDA).

Inflammation is a critical factor in COVID-19 [5,33], and, consequently, agents able to combat virus replication, and, at the same time, regulate inflammation, could offer a new approach for the treatment of COVID-19. Since GIIA sPLA_2_ is associated with increased mortality by COVID-19 [12], and lipid mediators arising from the activity of PLA_2_ have been correlated with severe SARS-CoV-2 infection in humans [34], a therapeutic compound able to simultaneously inhibit both SARS-CoV-2 M^pro^ and GIIA sPLA_2_ would be of great value, as it would significantly reduce the risk of COVID-19 mortality. For the first time, a dual inhibitor of GIIA sPLA_2_ and SARS-CoV-2 M^pro^ is identified. GK241 shows weak inhibitory activity against SARS-CoV-2 M^pro^ compared to other known 2-oxoamide SARS-CoV-2 M^pro^ inhibitors; however, it may represent a basis for the development of a new class of potent dual-action inhibitors.

## 4. Materials and Methods

### 4.1. General Chemistry Methods

Forced-flow chromatography on Merck^®^ (Merck, Darmstadt, Germany) Kieselgel 60 F_254_ 230–400 mesh was used for the purification of the products, while aluminum-backed silica plates (0.2 mm, 60 F_254_) were used for thin-layer chromatography (TLC). The visualization of the developed chromatograms was performed by fluorescence quenching using phosphomolybdic acid, ninhydrin or potassium permanganate stains. The melting points were determined on a Buchi^®^ 530 apparatus (Buchi, Flawil, Switzerland) and were uncorrected. Specific rotations were measured on an AA-65 series (Optical Activity Ltd., Bury, UK) polarimeter. ^1^H and ^13^C NMR spectra were recorded on a Varian^®^ Mercury (Varian, Palo Alto, CA, USA) (200 MHz and 50 MHz, respectively) or a Bruker Avance Neo (Bruker, Faellanden, Switzerland) (400 MHz and 100 MHz, respectively) and were internally referenced to residual solvent signals. The data for ^1^H NMR are reported as follows: chemical shift (δ ppm), multiplicity (s = singlet, d = doublet, t = triplet, qu = quintet, m = multiplet, and br s = broad signal), coupling constant, integration and peak assignment. The data for ^13^C NMR are reported in terms of the chemical shift (δ ppm). High-resolution mass spectrometry (HRMS) spectra were recorded on a Bruker^®^ Maxis Impact QTOF (Bruker Daltonics, Bremen, Germany) spectrometer.

### 4.2. General Procedure for the Coupling of α-Hydroxycarboxylic Acids **4a,b** with Amines **5a–g**

To a stirred solution of amine **5a**–**g** (1.0 mmol), cooled to 0 °C, triethylamine (2.2 mmol, 0.31 mL), EDC∙HCl (1.1 mmol, 211 mg), HOBt (1 mmol, 135 mg) and α-hydroxycarboxylic acid **4a,b** (1.0 mmol) were added consecutively. After stirring for 1 h at 0 °C and for 16 hrs at room temperature (r.t.), the solvent was removed under reduced pressure. The residue was diluted in EtOAc and washed with brine (10 mL), an aqueous solution of 1N HCl (10 mL), brine (10 mL), an aqueous solution of 5% NaHCO_3_ (10 mL) and brine (10 mL), consecutively. The organic layer was dried over Na_2_SO_4_ and concentrated under reduced pressure. The resulting α-hydroxyamide **6a**–**g** was further purified (if necessary) by flash chromatography eluting with the appropriate mixture of EtOAc:petroleum ether (40–60 °C).

#### 4.2.1. (2*S*)-*tert*-Butyl 2-(2-hydroxyhexadecanamido)propanoate (**6a**)

Yield 85%; White solid; mp: 56–57 °C; Diastereoisomer 1: ^1^H NMR (200 MHz, CDCl_3_): *δ* = 7.23–7.16 (m, 1H, NH), 4.40–4.23 (m, 2H, CH, OH), 4.02–3.97 (m, 1H, CH), 1.67–1.07 (m, 38H, 13 × CH_2_, 4 × CH_3_), 0.76 (t, *J* = 5.9 Hz, 3H, C*H_3_*CH_2_); ^13^C NMR (50 MHz, CDCl_3_): *δ* = 174.3, 172.4, 82.0, 71.9, 48.2, 34.7, 31.9, 29.7, 29.64, 29.58, 29.5, 29.3, 27.9, 25.0, 22.7, 18.4, 14.1; Diastereoisomer 2: ^1^H NMR (200 MHz, CDCl_3_): *δ* = 7.11 (d, *J* = 7.6 Hz, 1H, NH), 4.43 (dt, *J* = 14.5, 7.2 Hz, 1H, CH), 4.10–4.04 (m, 1H, CH), 3.40 (br s, 1H, OH), 1.78–1.12 (m, 38H, 13 × CH_2_, 4 × CH_3_), 0.85 (t, *J* = 6.4 Hz, 3H, C*H_3_*CH_2_); ^13^C NMR (50 MHz, CDCl_3_): *δ* = 173.9, 172.2, 82.2, 72.1, 48.4, 34.9, 32.0, 29.79, 29.75, 29.7, 29.6, 29.53, 29.46, 28.0, 25.1, 22.8, 18.6, 14.2; HRMS (ESI) [M + Na]^+^ *m/z*: 422.3243; (calculated for [C_23_H_45_NNaO_4_]^+^ 422.3241).

#### 4.2.2. *N*-((*S*)-1-((*tert*-Butyldimethylsilyl)oxy)-3-methylbutan-2-yl)-2-hydroxyhexadecanamide (**6b**)

Yield 80%; Colorless solid of low melting point; ^1^H NMR (200 MHz, CDCl_3_): *δ* = 6.81–6.73 (m, 1H, NH), 4.11–4.04 (m, 1H, CH), 3.76–3.40 (m, 5H, CH_2_O, 2 × CH, OH), 1.94–1.18 (m, 26H, 13 × CH_2_), 0.93–0.74 (m, 18H, 6 × CH_3_), 0.02 (s, 6H, 2 × SiCH_3_); ^13^C NMR (50 MHz, CDCl_3_): *δ* = 173.9, 173.7, 72.2, 72.0, 62.9, 55.4, 35.3, 35.2, 32.0, 29.81, 29.77, 29.7, 29.6, 29.5, 28.9, 25.9, 25.2, 25.0, 22.8, 19.7, 19.0, 18.3, 14.2, −5.5; HRMS (ESI) [M + Na]^+^ *m/z*: 494.4000; (calculated for [C_27_H_57_NNaO_3_Si]^+^ 494.4000).

#### 4.2.3. *N*-((*S*)-1-(Benzyloxy)-3-methylbutan-2-yl)-2-hydroxyhexadecanamide (**6c**)

Yield 77%; White solid; mp: 63–64 °C; ^1^H NMR (200 MHz, CDCl_3_): *δ* = 7.38–7.18 (m, 5H, 5 × ArH), 6.80–6.68 (m, 1H, NH), 4.54–4.41 (m, 2H, CH_2_Ar), 4.12–3.99 (m, 1H, C*H*OH), 3.91–3.79 (m, 1H, C*H*NH), 3.61–3.32 (m, 3H, CH, CH_2_O), 2.09–1.13 (m, 26H, 13 × CH_2_), 0.93–0.84 (m, 9H, 3 × CH_3_); ^13^C NMR (50 MHz, CDCl_3_): *δ* = 173.99, 173.96, 138.15, 138.10, 128.5, 127.9, 127.81, 127.76, 73.3, 72.3, 72.0, 70.21, 70.18, 54.0, 53.9, 35.2, 32.0, 29.81, 29.77, 29.72, 29.69, 29.6, 29.5, 29.3, 25.1, 25.0, 22.8, 19.6, 19.0, 14.2; HRMS (ESI) [M + H]^+^ *m/z*: 448.3785; (calculated for [C_28_H_50_NO_3_]^+^ 448.3785).

#### 4.2.4. *N*-((*S*)-1-(Butylamino)-3-methyl-1-oxobutan-2-yl)-2-hydroxyhexadecanamide (**6d**)

Yield 76%; White solid; mp: 95–97 °C; ^1^H NMR (200 MHz, CDCl_3_): *δ* = 7.53 (d, *J* = 9.3 Hz, 1H, N*H*CH), 7.19 (t, *J* = 5.8 Hz, 0.5H, N*H*CH_2_), 7.02 (t, *J* = 5.7 Hz, 0.5H, N*H*CH_2_), 4.97 (d, *J* = 4.9 Hz, 0.5H, CH), 4.50 (d, *J* = 5.3 Hz, 0.5H, CH), 4.32–4.20 (m, 1H, CH), 4.17–4.04 (m, 1H, CH), 3.36–3.01 (m, 3H, NHC*H_2_*, OH), 2.17–2.00 (m, 2H, CH_2_), 1.87–1.12 (m, 28H, 14 × CH_2_), 0.95–0.82 (m, 12H, 4 × CH_3_); ^13^C NMR (50 MHz, CDCl_3_): *δ* = 175.03, 174.96, 171.8, 171.6, 72.3, 58.7, 58.4, 39.42, 39.38, 35.1, 32.0, 31.5, 31.1, 29.83, 29.78, 29.5, 25.3, 25.2, 22.8, 20.23, 20.20, 19.4, 18.7, 18.6, 14.2, 13.8; HRMS (ESI) [M + Na]^+^ *m/z*: 449.3715; (calculated for [C_25_H_50_N_2_NaO_3_]^+^ 449.3714).

#### 4.2.5. (2*S*)-Methyl 2-(2-hydroxyhexadecanamido)-3-(2-oxopyrrolidin-3-yl)propanoate (**6e**)

Yield 57%; Yellow oil; ^1^H NMR (200 MHz, CDCl_3_): *δ* = 8.45 (d, *J* = 7.8 Hz, 0.17H, NH), 8.27 (d, *J* = 8.6 Hz, 0.17H, NH), 7.97 (d, *J* = 7.4 Hz, 0.33H, NH), 7.84 (d, *J* = 7.9 Hz, 0.33H, NH), 6.69 (s, 0.17H, NH), 6.59 (s, 0.17H, NH), 6.54 (s, 0.33H, NH), 6.46 (s, 0.33H, NH), 4.64–4.49 (m, 1H, CH), 4.14–4.04 (m, 1H, CH), 3.71 (s, 3H, OCH_3_), 3.51–3.40 (m, 1H, C*H*H), 3.36–3.28 (m, 1H, CH*H*), 2.54–2.04 (m, 4H, CH_2_, CH, OH), 1.94–1.53 (m, 4H, 2 × CH_2_), 1.49–0.94 (m, 24H, 12 × CH_2_), 0.85 (t, *J* = 5.9 Hz, 3H, CH_3_); HRMS (ESI) [M + Na]^+^ *m/z*: 463.3139; (calculated for [C_24_H_44_N_2_NaO_5_]^+^ 463.3142).

#### 4.2.6. (2*S*)-*tert*-Butyl 2-((2*S*)-2-(2-hydroxyhexadecanamido)propanamido)propanoate (**6f**)

Yield 75%; White solid; mp: 65–66 °C; ^1^H NMR (200 MHz, CDCl_3_): *δ* = 7.41 (d, *J* = 7.9 Hz, 1H, NH), 7.28–7.23 (m, 1H, NH), 4.65–4.51 (m, 1H, CH), 4.42–4.28 (m, 1H, CH), 4.18–4.03 (m, 2H, CH, OH), 2.01–1.10 (m, 41H, 13 × CH_2_, 5 × CH_3_), 0.84 (t, *J* = 6.5 Hz, 3H, CH_2_C*H_3_*); ^13^C NMR (50 MHz, CDCl_3_): *δ* = 174.7, 174.6, 172.3, 172.1, 171.9, 171.8, 81.98, 81.95, 72.1, 72.0, 48.9, 48.4, 34.9, 32.0, 29.8, 29.74, 29.66, 29.6, 29.4, 28.0, 25.1, 22.8, 18.8, 18.6, 18.2, 18.1, 14.2; HRMS (ESI) [M + Na]^+^ *m/z*: 493.3612; (calculated for [C_26_H_50_N_2_NaO_5_]^+^ 493.3612).

#### 4.2.7. (2*S*)-*tert*-Butyl 2-(2-hydroxydodecanamido)-3-methylbutanoate (**6g**)

Yield 87%; White solid of low melting point; ^1^H NMR (400 MHz, CDCl_3_): *δ* = 6.94 (d, *J* = 9.3 Hz, 0.5H, NH), 6.76 (d, *J* = 9.4 Hz, 0.5H, NH), 4.45 (dd, *J* = 9.1, 4.5 Hz, 1H, CH), 4.14 (dd, *J* = 7.8, 3.8 Hz, 1H, CH), 2.24–2.14 [m, 1H, C*H*(CH_3_)_2_], 1.87–1.79 (m, 1H, C*H*HCHOH), 1.69–1.59 (m, 1H, CH*H*CHOH), 1.47–1.25 (m, 25H, 8 × CH_2_, 3 × CH_3_), 0.98–0.86 (m, 9H, 3 × CH_3_); ^13^C NMR (100 MHz, CDCl_3_): *δ* =173.94, 173.89, 171.3, 171.1, 82.2, 72.5, 72.1, 57.3, 57.1, 35.3, 35.1, 32.0, 31.6, 31.5, 29.72, 29.70, 29.65, 29.6, 29.52, 29.46, 28.2, 25.1, 25.0, 22.8, 19.12, 19.07, 17.8, 17.7, 14.2; HRMS (ESI) [M + Na]^+^ *m/z*: 394.2928; (calculated for [C_21_H_41_NNaO_4_]^+^ 394.2928).

### 4.3. General Procedure for the Oxidation of Hydroxyamides **6a**–**g** to Oxoamides **7a**–**g**

To a stirred solution of α-hydroxy-amides (**6a**–**g)** (1 mmol) in dry CH_2_Cl_2_ (0.2 M), under an inert argon atmosphere, Dess–Martin periodinane (1.3 mmol, 551 mg) was added. After stirring for 1 h, the solvent was removed under reduced pressure and Et_2_O (30 mL) was added. The organic layer was washed with a saturated solution of aqueous NaHCO_3_ (20 mL) containing Na_2_S_2_O_3_ (1.5 g, 9.5 mmol), H_2_O (20 mL), and dried over Na_2_SO_4_. After removal of the organic solvent under reduced pressure, the residue was purified by flash chromatography eluting with the appropriate mixture of EtOAc:petroleum ether (40–60 °C).

#### 4.3.1. (*S*)-*tert*-Butyl 2-(2-oxohexadecanamido)propanoate (**7a**)

Yield 87%; White solid; mp: 44–45 °C; [α]_D_ = +5 (c = 1 in CHCl_3_); ^1^H NMR (400 MHz, CDCl_3_): *δ* = 7.42 (d, *J* = 7.5 Hz, 1H, NH), 4.35 (qu, *J* = 7.2 Hz, 1H, C*H*NH), 2.83 (t, *J* = 7.3 Hz, 2H, CH_2_CO), 1.57–1.50 (m, 2H, C*H_2_*CH_2_CO), 1.41 (s, 9H, 3 × CCH_3_), 1.35 (d, *J* = 7.1 Hz, 3H, CHC*H_3_*), 1.27–1.09 (m, 22H, 11 × CH_2_), 0.81 (t, *J* = 6.5 Hz, 3H, CH_2_C*H_3_*); ^13^C NMR (100 MHz, CDCl_3_): *δ* = 198.5, 171.1, 159.6, 82.2, 48.6, 36.7, 31.9, 29.70, 29.69, 29.67, 29.6, 29.5, 29.38, 29.35, 29.1, 27.9, 23.2, 22.7, 18.2, 14.1; HRMS (ESI) [M + Na]^+^ *m/z*: 420.3084; (calculated for [C_23_H_43_NNaO_4_]^+^ 420.3084).

#### 4.3.2. (*S*)-*N*-(1-((*tert*-Butyldimethylsilyl)oxy)-3-methylbutan-2-yl)-2-oxohexadecanamide (**7b**)

Yield 89%; Colorless oil; [α]_D_ = −19 (c = 1 in CHCl_3_); ^1^H NMR (400 MHz, CDCl_3_): *δ* = 7.12 (d, *J* = 9.6 Hz, 1H, NH), 3.73 (dd, *J* = 10.2, 3.3 Hz, 1H, C*H*HO), 3.64 (ddd, *J* = 13.3, 7.4, 3.6 Hz, 1H, C*H*NH), 3.56 (dd, *J* = 10.2, 3.9 Hz, 1H, CH*H*O), 2.89 (t, *J* = 7.4 Hz, 2H, CH_2_CO), 1.99–1.87 (m, 1H, C*H*CHNH), 1.62–1.55 (m, 2H, C*H_2_*CH_2_CO), 1.34–1.18 (m, 22H, 11 × CH_2_), 0.93 (d, *J* = 6.8 Hz, 3H, C*H_3_*CH_2_), 0.89–0.84 (m, 15H, 2 × C*H_3_*CH, 3 × CH_3_C), 0.01 (d, *J* = 1.9 Hz, 6H, 2 × CH_3_Si); ^13^C NMR (100 MHz, CDCl_3_): *δ* = 199.6, 160.0, 62.7, 56.1, 36.9, 32.0, 29.80, 29.79, 29.77, 29.7, 29.6, 29.5, 29.2, 29.0, 25.9, 23.4, 22.8, 19.6, 19.0, 18.3, 14.2, −5.45, −5.47; HRMS (ESI) [M + Na]^+^ *m/z*: 492.3843; (calculated for [C_27_H_55_NNaO_3_Si]^+^ 492.3843).

#### 4.3.3. (*S*)-*N*-(1-(Benzyloxy)-3-methylbutan-2-yl)-2-oxohexadecanamide (**7c**)

Yield 93%; White solid; mp: 47–48 °C; [α]_D_ = −34 (c = 1 in CHCl_3_); ^1^H NMR (400 MHz, CDCl_3_): *δ* = 7.37–7.28 (m, 5H, 5 × ArH), 7.17 (d, *J* = 9.7 Hz, 1H, NH), 4.51 (d and d, *J* = 12.1 Hz, 2H, CH_2_Ph), 3.87–3.80 (m, 1H, C*H*NH), 3.61 (dd, *J* = 9.7, 4.2 Hz, 1H, C*H*HO), 3.46 (dd, *J* = 9.7, 4.0 Hz, 1H, CH*H*O), 3.00–2.86 (m, 2H, CH_2_CO), 2.05–1.97 (m, 1H, C*H*CHNH), 1.66–1.59 (m, 2H, C*H_2_*CH_2_CO), 1.39–1.20 (m, 22H, 11 × CH_2_), 0.96–0.89 (m, 9H, 2 × C*H_3_*CH, C*H_3_*CH_2_); ^13^C NMR (100 MHz, CDCl_3_): *δ* = 199.5, 160.1, 138.0, 128.5, 127.8, 127.7, 73.3, 69.7, 54.6, 36.9, 32.0, 29.8, 29.75, 29.73, 29.68, 29.5, 29.43, 29.36, 29.2, 23.3, 22.8, 19.5, 18.9, 14.2; HRMS (ESI) [M + Na]^+^ *m/z*: 468.3448; (calculated for [C_28_H_47_NNaO_3_]^+^ 468.3448).

#### 4.3.4. (*S*)-*N*-(1-(Butylamino)-3-methyl-1-oxobutan-2-yl)-2-oxohexadecanamide (**7d**)

Yield 89%; White solid; mp: 78–79 °C; [α]_D_ = −20 (c = 1 in CHCl_3_); ^1^H NMR (400 MHz, CDCl_3_): *δ* = 7.56 (d, *J* = 9.2 Hz, 1H, NHCO), 6.52 (br s, 1H, N*H*CH_2_), 4.18–4.14 (m, 1H, C*H*NH), 3.33–3.25 (m, 1H, C*H*HNH), 3.18–3.10 (m, 1H, CH*H*NH), 2.91–2.76 (m, 2H, CH_2_CO), 2.17–2.07 (m, 1H, C*H*CHNH), 1.61–1.53 (m, 2H, CH_2_), 1.49–1.42 (m, 2H, CH_2_), 1.34–1.13 (m, 24H, 12 × CH_2_), 0.94–0.82 (m, 12H, 4 × CH_3_); ^13^C NMR (100 MHz, CDCl_3_): *δ* = 198.3, 170.1, 160.3, 59.0, 39.4, 36.9, 32.0, 31.7, 31.3, 29.74, 29.73, 29.71, 29.66, 29.5, 29.4, 29.2, 23.2, 22.7, 20.2, 19.3, 18.4, 14.2, 13.8; HRMS (ESI) [M + Na]^+^ *m/z*: 447.3557; (calculated for [C_25_H_48_N_2_NaO_3_]^+^ 447.3557).

#### 4.3.5. (2*S*)-Methyl 2-(2-oxohexadecanamido)-3-(2-oxopyrrolidin-3-yl)propanoate (**7e**)

Yield 57%; White solid; mp: 59–61 °C; [α]_D_ = −3 (c = 1 in CHCl_3_); ^1^H NMR (400 MHz, CDCl_3_): *δ* = 8.91 (d, *J* = 7.8 Hz, 0.25H, NH), 8.19 (d, *J* = 7.9 Hz, 0.75H, NH), 7.07 (s, 0.25H, NH), 6.88 (s, 0.75H, NH), 4.66–4.61 (m, 0.25H, NHC*H*), 4.54–4.48 (m, 0.75H, NHC*H*), 3.72 (s, 3H, CH_3_O), 3.37–3.27 (m, 2H, NHC*H_2_*), 2.85 (t, *J* = 7.4 Hz, 2H, CH_2_CO), 2.50–2.14 (m, 3H, CH_2_, CH), 2.02–1.78 (m, 2H, CH_2_), 1.60–1.52 (m, 2H, CH_2_), 1.32–1.18 (m, 22H, 11 × CH_2_), 0.84 (t, *J* = 6.9 Hz, 3H, C*H_3_*CH_2_); ^13^C NMR (100 MHz, CDCl_3_): *δ* = 198.4, 198.2, 179.9, 179.6, 171.5, 171.4, 160.7, 160.6, 52.7, 52.6, 51.5, 51.3, 40.8, 40.6, 38.5, 38.4, 37.0, 36.9, 33.4, 32.6, 32.0, 29.74, 29.72, 29.70, 29.66, 29.52, 29.51, 29.4, 29.1, 28.5, 28.4, 23.21, 23.18, 22.7, 14.2; HRMS (ESI) [M + Na]^+^ *m/z*: 461.2990; (calculated for [C_24_H_42_N_2_NaO_5_]^+^ 461.2986).

#### 4.3.6. (*S*)-*tert*-Butyl 2-((*S*)-2-(2-oxohexadecanamido)propanamido)propanoate (**7f**)

Yield 82%; White solid; mp: 92–94 °C; [α]_D_ = −12 (c = 1 in CHCl_3_); ^1^H NMR (400 MHz, CDCl_3_): *δ* = 7.58 (d, *J* = 7.3 Hz, 1H, NHCOCO), 6.82–6.71 (m, 1H, N*H*CHCOO), 4.49 (qu, *J* = 7.1 Hz, 1H, C*H*NHCOCO), 4.41 (qu, *J* = 7.2 Hz, 1H, CHCOO), 2.86 (t, *J* = 7.2 Hz, 2H, CH_2_CO), 1.60–1.53 (m, 2H, C*H_2_*CH_2_CO), 1.43 (s, 9H, 3 × CCH_3_), 1.41 (d, *J* = 7.0 Hz, 3H, C*H_3_*CHNHCOCO), 1.33 (d, *J* = 7.2 Hz, 3H, C*H_3_*CHCOO), 1.29–1.16 (m, 22H, 11 × CH_2_), 0.84 (t, *J* = 6.7 Hz, 3H, C*H_3_*CH_2_); ^13^C NMR (100 MHz, CDCl_3_): *δ* = 198.4, 171.9, 170.7, 160.0, 82.2, 48.9, 36.9, 32.0, 29.8, 29.74, 29.71, 29.66, 29.5, 29.4, 29.1, 28.0, 23.2, 22.8, 18.54, 18.46, 14.2; HRMS (ESI) [M + Na]^+^ *m/z*: 491.3455; (calculated for [C_26_H_48_N_2_NaO_5_]^+^ 491.3455).

#### 4.3.7. (*S*)-*tert*-Butyl 3-methyl-2-(2-oxododecanamido)butanoate (**7g**)

Yield 67%; Colorless oil; [α]_D_ = +14 (c = 1 in CHCl_3_); ^1^H NMR (400 MHz, CDCl_3_): *δ* = 7.34 (d, *J* = 9.2 Hz, 1H, NH), 4.33 (dd, *J* = 9.1, 4.6 Hz, 1H, C*H*NH), 2.86 (t, *J* = 7.4 Hz, 2H, CH_2_CO), 2.23–2.12 (m, 1H, C*H*CHNH), 1.61–1.53 (m, 2H, C*H_2_*CH_2_CO), 1.44 (s, 9H, 3 × CCH_3_), 1.32–1.15 (m, 14H, 7 × CH_2_), 0.90 (dd, *J* = 7.0, 5.8 Hz, 6H, 2 × CHC*H_3_*), 0.84 (t, *J* = 6.9 Hz, 3H, CH_2_C*H_3_*); ^13^C NMR (100 MHz, CDCl_3_): *δ* = 198.7, 170.1, 160.1, 82.4, 57.6, 36.8, 32.0, 31.6, 29.6, 29.5, 29.39, 29.36, 29.1, 28.1, 23.3, 22.7, 19.0, 17.6, 14.2; HRMS (ESI) [M + Na]^+^ *m/z*: 392.2769; (calculated for [C_21_H_39_NNaO_4_]^+^ 392.2771).

### 4.4. General Procedure of Deprotection of tert-Butyl Esters to Carboxylic Acids **8a**–**c**

To a stirred solution of *tert*-butyl ester **7a,f,g** (1 mmol) in dry CH_2_Cl_2_ (1 mL), TFA (1 mL) was added and the reaction mixture was left stirring for 3 hrs. After removal of the solvent, the residue was diluted in diethyl ether and precipitation by petroleum ether (40–60 °C) and filtration afforded the desired product.

#### 4.4.1. (*S*)-2-(2-Oxohexadecanamido)propanoic Acid (**8a**)

Yield 96%; White solid; mp: 101–103 °C; [α]_D_ = +13 (c = 1 in CHCl_3_); ^1^H NMR (400 MHz, CDCl_3_): *δ* = 8.88 (br s, 1H, COOH), 7.42 (d, *J* = 7.6 Hz, 1H, NH), 4.58 (qu, *J* = 7.3 Hz, 1H, C*H*CH_3_), 2.90 (t, *J* = 7.3 Hz, 2H, CH_2_CO), 1.65–1.56 (m, 2H, C*H_2_*CH_2_CO), 1.52 (d, *J* = 7.2 Hz, 3H, CHC*H_3_*), 1.36–1.20 (m, 22H, 11 × CH_2_), 0.87 (t, *J* = 6.6 Hz, 3H, CH_2_C*H_3_*); ^13^C NMR (100 MHz, CDCl_3_): *δ* = 198.4, 176.8, 160.0, 48.1, 36.8, 32.0, 29.77, 29.75, 29.7, 29.54, 29.45, 29.4, 29.1, 23.2, 22.8, 17.8, 14.2; HRMS (ESI) [M-H]^-^ *m/z*: 340.2492; (calculated for [C_19_H_34_NO_4_]^−^ 340.2493).

#### 4.4.2. (*S*)-2-((*S*)-2-(2-Oxohexadecanamido)propanamido)propanoic Acid (**8b**)

Yield 84%; White solid; mp: 157–158 °C; [α]_D_ = +8 (c = 1 in DMF); ^1^H NMR (400 MHz, DMSO-d_6_): *δ* = 12.52 (br s, 1H, COOH), 8.35 (d, *J* = 7.9 Hz, 1H, NHCOCO), 8.25 (d, *J* = 7.3 Hz, 1H, N*H*CHCOOH), 4.32 (qu, *J* = 7.1 Hz, 1H, C*H*NHCOCO), 4.20 (qu, *J* = 7.3 Hz, 1H, C*H*COOH), 2.78 (t, *J* = 7.3 Hz, 2H, CH_2_CO), 1.55–1.43 (m, 2H, C*H_2_*CH_2_CO), 1.30–1.20 (m, 28H, 11 × CH_2_, 2 × C*H_3_*CH), 0.86 (t, *J* = 6.8 Hz, 3H, C*H_3_*CH_2_); ^13^C NMR (100 MHz, DMSO-d_6_) *δ* = 198.8, 173.8, 171.1, 160.6, 47.9, 47.5, 36.5, 31.3, 29.03, 29.02, 29.00, 28.99, 28.96, 28.84, 28.78, 28.7, 28.4, 22.7, 22.1, 18.0, 17.1, 13.9; HRMS (ESI) [M-H]^−^ *m/z*: 411.2864; (calculated for [C_22_H_39_N_2_O_5_]^−^ 411.2864).

#### 4.4.3. (*S*)-3-Methyl-2-(2-oxododecanamido)butanoic Acid (**8c**)

Yield 59%; White solid; mp: 53–54 °C; [α]_D_ = +6 (c = 1 in CHCl_3_); ^1^H NMR (400 MHz, CDCl_3_): *δ* = 10.59 (s, 1H, COOH), 7.39 (d, *J* = 9.1 Hz, 1H, NH), 4.57–4.44 (m, 1H, C*H*NH), 2.89 (t, *J* = 7.5 Hz, 2H, CH_2_CO), 2.36–2.23 (m, 1H, C*H*(CH_3_)_2_), 1.65–1.52 (m, 2H, C*H_2_*CH_2_CO), 1.36–1.16 (m, 14H, 7 × CH_2_), 1.03–0.79 (m, 9H, 3 × CH_3_); ^13^C NMR (100 MHz, CDCl_3_): *δ* = 198.5, 176.1, 160.3, 57.2, 36.9, 32.0, 31.2, 29.6, 29.5, 29.43, 29.40, 29.1, 23.3, 22.8, 19.1, 17.6, 14.2; HRMS (ESI) [M-H]^−^ *m/z*: 312.2176; (calculated for [C_17_H_30_NO_4_]^−^ 312.2180).

### 4.5. (S)-1-((tert-Butyldimethylsilyl)oxy)-3-methylbutan-2-amine (**5b**)

To a solution of benzyl (*S*)-(1-((*tert*-butyldimethylsilyl)oxy)-3-methylbutan-2-yl)carbamate (1.0 mmol, 353 mg) in MeOH (10 mL), 10% Pd/C (0.05 mmol, 53 mg) was added, and the reaction was left stirring under H_2_ for 16 hrs. Upon completion, the reaction mixture was filtered through celite, and the solvent was evaporated under reduced pressure to afford the desired product. Yield 89%; Pale yellow solid of low melting point; [α]_D_ = +4 (c = 1 in CHCl_3_); ^1^H NMR (200 MHz, CDCl_3_): *δ* = 3.65 (dd, *J* = 9.9, 4.2 Hz, 1H, C*H*H), 3.43 (dd, *J* = 9.9, 7.2 Hz, 1H, CH*H*), 3.01 (br s, 2H, NH_2_), 2.66–2.57 (m, 1H, C*H*NH_2_), 1.77–1.61 (m, 1H, C*H*(CH_3_)_2_), 1.04–0.71 (m, 15H, 5 × CH_3_), 0.03 (s, 6H, 2 × CH_3_Si); HRMS (ESI) [M + H]^+^ *m/z*: 218.1934; (calculated for [C_11_H_28_NOSi]^+^ 218.1935).

### 4.6. (S)-tert-Butyl (1-(benzyloxy)-3-methylbutan-2-yl)carbamate (**10**)

To a flame-dried flask, under argon, NaH 60% (1.3 mmol, 52 mg) and dry *N*,*N*-dimethylformamide (DMF) (1.3 mL) were added. A solution of alcohol **9** (1.0 mmol, 203 mg) in dry DMF (0.7 mL) was added dropwise at 0 °C. The reaction mixture was stirred for 30 min at 0 °C and then benzyl bromide (1.1 mmol, 0.13 mL) was added dropwise. The reaction mixture was left stirring for 16 hrs at room temperature. Upon completion, the reaction mixture was quenched with a saturated aqueous solution of NH_4_Cl (1 mL), H_2_O (3 mL) was added, the aqueous layer was extracted with ethyl acetate (2 × 10 mL) and the combined organic layers were washed with H_2_O (15 mL). The organic layer was collected, and after drying over Na_2_SO_4_, the solvent was removed under reduced pressure. The product was purified by flash chromatography eluting with a mixture of EtOAc:petroleum ether (40–60 °C)–5:95. Yield 47%; Colorless oil; [α]_D_ = −20 (c = 1 in CHCl_3_); ^1^H NMR (200 MHz, CDCl_3_): *δ* = 7.44–7.13 (m, 5H, 5 × ArH), 4.78 (d, *J* = 7.9 Hz, 1H, NH), 4.56–4.42 (m, 2H, CH_2_Ph), 3.61–3.37 (m, 3H, C*H_2_*CH, C*H*NH), 1.99–1.82 (m, 1H, C*H*(CH_3_)_2_), 1.45 (s, 9H, C(CH_3_)_3_), 0.92 (d, *J* = 6.8 Hz, 3H, CH_3_), 0.91 (d, *J* = 6.8 Hz, 3H, CH_3_); ^13^C NMR (50 MHz, CDCl_3_): *δ* = 155.9, 138.3, 128.4, 127.62, 127.58, 78.9, 73.1, 70.5, 55.5, 29.6, 28.4, 19.6, 18.7; HRMS (ESI) [M + H]^+^ *m/z*: 294.2064; (calculated for [C_17_H_28_NO_3_]^+^ 294.2064).

### 4.7. (S)-1-(Benzyloxy)-3-methylbutan-2-aminium chloride (**5c**)

*N*-Boc-Protected amine **10** (1.0 mmol, 294 mg) was stirred for 2 hrs with a 4N solution of HCl in MeOH (50.0 mmol, 12.5 mL). Upon completion of the reaction, Et_2_O was added, and the solvents were removed under reduced pressure. The latter was repeated until complete removal of HCl, to afford the desired product. Yield 100%; White solid; mp: 115–118 °C; [α]_D_ = +20 (c = 1 in MeOH); ^1^H NMR (200 MHz, CD_3_OD): *δ* = 7.41–7.25 (m, 5H, 5 × ArH), 4.65–4.52 (m, 2H, CH_2_Ph), 3.73–3.56 (m, 2H, C*H_2_*CH), 3.16–3.08 (m, 1H, C*H*NH_2_), 2.11–1.93 (m, 1H, C*H*(CH_3_)_2_), 1.03 (d, *J* = 6.8 Hz, 3H, CH_3_), 0.97 (d, *J* = 6.8 Hz, 3H, CH_3_); HRMS (ESI) [M + H]^+^ *m/z*: 194.1535; (calculated for [C_12_H_20_NO]^+^ 194.1539).

### 4.8. Enzyme Assay

The enzyme inhibition assay was performed as previously described [15]. A buffer composed of 20 mM Tris, 100 mM NaCl, 1 mM EDTA, pH 7.3 was used for the enzyme inhibition assay. For the determination of the inhibition rate, 0.5 μM of SARS-CoV-2 M^pro^ was incubated with 40 μM or 100 μM of 2-oxoamide in the buffer at 37 °C for 10 min. The FRET substrate was then added to each well at a final concentration of 10 μM and a final total volume of 50 μL, to initiate the reaction. The GraphPad Prism 6.0 software (GraphPad) was used for the calculation of % inhibition rate. Measurements of the inhibition rate for the compounds were performed in triplicate and are presented as mean ± SD.

### 4.9. Covalent Docking Calculations

#### 4.9.1. Protein Preparation

Docking calculations were performed using the SARS-CoV-2 M^pro^ crystallographic structure in complex with the covalent α-ketoamide inhibitor **13b** (PDB ID: 6Y2F) [15]. Preparation and minimization of M^pro^, using the Protein Preparation Wizard tool within the Maestro Schrodinger suite, were performed to ensure structural correctness. Hydrogen addition, bond orders and steric clashes correction, water molecules and HetAtoms deletion, charge optimization and restrained minimization supported by the OPLS3 force field were achieved [35]. Moreover, addition of the missing residues E47 and D48 was performed using the Crosslink Proteins tool, in the Maestro Schrodinger suite. The inhibitor GK241 and analogs **8a** and **8d** were prepared for docking using the LigPrep tool, in the Maestro Schrodinger suite [36].

#### 4.9.2. Covalent Docking

Covalent docking is a multiple step process, that is designed upon Schroedinger’s Glide and Prime, capable to determine ligands activity against a protein target taking into account both non-covalent interactions and covalent bond formation. Covalent docking calculations were carried out using the Covalent Docking application, implemented in Maestro Schrodinger suite. Initially, pose selection was carried out using non-covalent docking simulations (Glide) and positional constraints. Specifically, ligand docking was performed in a mutated binding site. The reactive residue was transformed to alanine and the ligand warhead (the ligand moiety able to form covalent bond) was docked closely to the catalytic residue avoiding unfavorable clashes. Subsequently, the mutation was reversed, and receptor sampling was performed. Covalent bond formation was achieved based on geometric criteria and structural optimization. The following step involved both minimization of protein–ligand complexes in vacuum, and clustering of the optimized poses. This early selection was used as a basis for the further minimization, scoring and ranking of covalent docking poses using the Prime VSGB2.0 energy model. Finally, an additional scoring function generated the affinity score that represents an average value of both the pre-reaction and post-reaction Glide Scores assessing the overall covalent docking procedure. Simultaneously, MM-GBSA energy property calculations for the structure of the receptor, the ligand and the protein-ligand complex were carried out for every docking pose [37]. In this case, Cys145 was identified as the reactive residue in M^pro^ and nucleophilic addition to a double bond as the reaction type. MM-GBSA scoring was selected and an output of 100 poses per ligand reaction site was achieved [37]. A covalent bond was formed between the reactive residue, Cys145 and the C2 carbonyl of the inhibitor. Subsequently, M^pro^–ligand complexes with thiohemiketal formation between ligand the 2-oxo group and the catalytic Cys145 were selected.

### 4.10. Molecular Dynamics Simulations

MD simulations were performed using Desmond software, which offers a simple setup. Each calculation comprised an eight-step, automated workflow, divided into two categories. A seven-step protocol enabling the system to be minimized and equilibrated was followed by simulation during the last step of the process. This workflow process allowed for the study of protein–ligand interactions and their conformational variations over time and evaluation of the effects of water molecules in the complex [38]. Initially, the System Builder tool in Desmond was used for the preparation of the complexes. TIP3P was selected as a solvent model, OPLS_2005 was assigned as the force field and the system was embedded in a triclinic shaped box. The volume of the box was minimized, and the negative charges were neutralized by the addition of Na**^+^** ions and 0.15 M of salt was added. Subsequently, MD simulations of 50 ns were performed in Desmond2020-1 using an NPT ensemble. A Nose–Hoover chain thermostat and Martyna–Tobias–Klein barostat were applied to maintain the temperature and pressure constant at 300 Kelvin and 1.01325 bar, respectively.

## 5. Conclusions

In this paper, we present our results on the identification of a synthetic 2-oxoamide inhibitor, which is a known potent inhibitor of GIIA sPLA_2_ (IC_50_ 143 nM) [24], and, at the same time, can weakly inhibit SARS-CoV-2 M^pro^ (IC_50_ 24 μM). The known 2-oxoamides AX109 and AX074, which selectively inhibit GIVA cPLA_2_, as well as pentafluoroethyl ketone GK187, which is a selective and potent inhibitor of GVIA iPLA_2_, did not exhibit any appreciable inhibition of SARS-CoV-2 M^pro^. The free carboxyl group and the long chain of the inhibitor GK241 were necessary for the inhibition of SARS-CoV-2 M^pro^. Since the role of GIIA sPLA_2_ in mortality from COVID-19 has recently been recognized [12], the development of dual inhibitors of GIIA sPLA_2_ and SARS-CoV-2 M^pro^ appears to represent an attractive strategy for the development of novel agents to treat COVID-19. 2-Oxoamide GK241 may provide a lead structure for the development of such dual inhibitors.

## Figures and Tables

**Table 1:** In vitro inhibition of SARS-CoV-2 M^pro^ by known PLA_2_ inhibitors.

Entry	Code	Structure	Inhibition Rate (%) (40 μM) ^a^	Inhibition Rate (%) (100 μM) ^a^
1	AX109	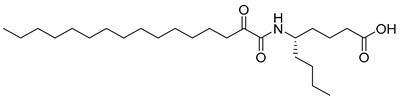	20.38 ± 2.75	32.11 ± 5.44
2	AX074	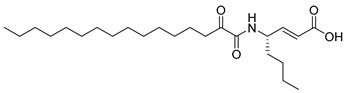	47.83 ± 3.27	50.52 ± 2.63
3	GK241	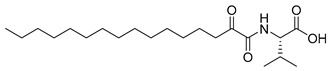	76.90 ± 0.41	97.33 ± 1.15
4	GK187	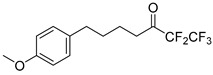	26.72 ± 3.50	32.62 ± 4.67

^a^ [SARS-CoV-2 M^pro^]: 0.5 μM, buffer: 20 mM Tris, 100 mM NaCl, 1 mM EDTA, pH 7.3.

**Table 2:** In vitro inhibition of SARS-CoV-2 M^pro^ by GK241 analogs.

Entry	Code	Structure	Inhibition Rate (%) ^a^ (40 μM)
1	**7a**	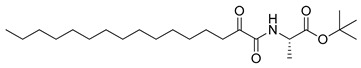	- ^b^
2	**7b**	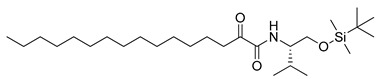	- ^b^
3	**7c**	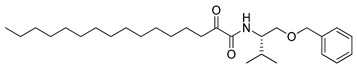	- ^b^
4	**7d**	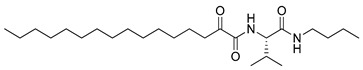	- ^b^
5	**7e**	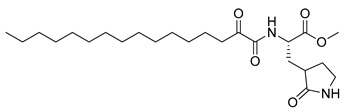	- ^b^
6	**7f**	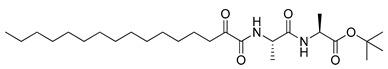	3.83 ± 1.34
7	**8a**	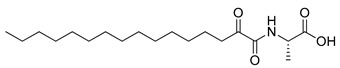	65.49 ± 2.79
8	**8b**	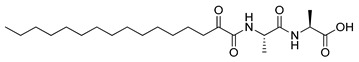	6.21 ± 5.24
9	**8d**	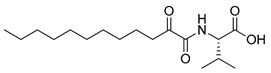	- ^c^

^a^ [SARS-CoV-2 M^pro^]: 0.5 μM, buffer: 20 mM Tris, 100 mM NaCl, 1 mM EDTA, pH 7.3. ^b^ Enhanced the enzymatic activity. ^c^ No inhibition.

## Data Availability

All data are available in the main text or the Supplementary Materials.

## Supplementary Materials

The following supporting information can be downloaded at https://www.mdpi.com/article/10.3390/ph15080961/s1: Table S1. Structural details of structures 1–7 concerning the configuration of 2-carbon and the dihedral angle Cβ_C145_-S_C145_-C2-O2 related to the covalent bond formatted between Cys145 and 2-oxoamide moiety. Molecular dynamics RMSDs for the protein and the ligand along with MM-GBSA binding energy; Figure S1. Structural details for the M^pro^-GK241 interactions in structures 1–6 resulting from covalent docking calculations; Figure S2. Protein residues root mean square fluctuation during the 50 nsec MD simulation showing local changes in the protein chain. Figure S3. Structures of **8d** (**A**) in magenta and **8a** (**B**) in blue bound to M^pro^ in comparison with GK241 (**C**) in green and crystal structure PDB 6Y2F (**D**) in khaki. The side-chain in derivative **8d** adopts a curved conformation exploring part of S1 and S2 protease cavities. ^1^H NMR and ^13^C NMR spectra of the compounds synthesized.

## Abbreviations

AA	arachidonic acid
DMF	*N*,*N*-dimethylformamide
EDC∙HCl	1-ethyl-3-(3-dimethylaminopropyl)carbodiimide hydrochloride
EDTA	ethylene diamine tetra-acetic acid
FDA	Food and Drug Administration
FFAs	free fatty acids
FRET	fluorescence resonance energy transfer
GIIA sPLA_2_	secreted PLA_2_
GIVA cPLA_2_	cytosolic PLA_2_
GVIA iPLA_2_	calcium-independent PLA_2_
HOBt	1-hydroxybenzotriazole
HRMS	high-resolution mass spectrometry
M^pro^	main protease
MD	molecular dynamics
OA	oleic acid
PCs	phosphatidylcholines
PLA_2_	phospholipase A_2_
RdRp	RNA-dependent RNA polymerase
r.t.	room temperature
SARS-CoV-2	severe acute respiratory syndrome coronavirus-2
TFA	trifluoroacetic acid
THF	tetrahydrofuran
TLC	thin-layer chromatography
Tris	tris(hydroxymethyl)aminomethane
