# Supplementary material for: Identification of a Dual Inhibitor of Secreted Phospholipase A2 (GIIA sPLA2) and SARS-CoV-2 Main Protease
Source: Pharmaceuticals (Basel). 2022 Aug 3;15(8):961. doi: 10.3390/ph15080961 (PMC9414318; doi:10.3390/ph15080961)
Supplement: Supplementary file 1 [file pharmaceuticals-15-00961-s001.zip › pharmaceuticals-1685211-supplementary.pdf]

Identification of a Dual Inhibitor of Secreted Phospholipase A_2 (GIIA $sPLA_2$) and SARS-CoV-2 Main Protease

Maria A. Theodoropoulou [1,2], Giorgos S. Koutoulogenis [1,2], Linlin Zhang [3], Ifigeneia Akrani [4], Emmanuel Mikros [4,5], Rolf Hilgenfeld [3,6] and George Kokotos [1,2,*]

[1]*Department of Chemistry, National and Kapodistrian University of Athens, Panepistimiopolis, 15771 Athens, Greece*

[2]*Center of Excellence for Drug Design and Discovery, National and Kapodistrian University of Athens, 15771 Athens, Greece*

[3] *Institute of Molecular Medicine, University of Lübeck, Ratzeburger Allee 160, 23562 Lübeck, Germany*

[4] *Department of Pharmacy, National and Kapodistrian University of Athens, Panepistimiopolis, 15771 Athens, Greece*

[5] *Athena Research and Innovation Center in Information Communication & Knowledge Technologies, Marousi, 15125, Greece*

[6] *German Center for Infection Research (DZIF), Hamburg – Lübeck – Borstel – Riems Site, University of Lübeck, 23562 Lübeck, Germany*

*To whom correspondence should be addressed. For G.K.: phone: +30 210 7274462; fax: +30 210 7274761; E-mail: gkokotos@chem.uoa.gr.

SUPPLEMENTARY MATERIALS

Contents

	**Page**
**Table S1.** Structural details of structures 1-7 concerning the configuration of 2-carbon and the dihedral angle $C\beta_{C145}$-S_{C145}-C2-O2 related to the covalent bond formatted between Cys145 and 2-oxoamide moiety. Molecular Dynamics RMSDs for the protein and the ligand along with MM-GBSA Binding Energy.	**2**
**Figure S1.** Structural details for the M^{pro}-GK241 interactions in structures 1-6 resulted by covalent docking calculations.	**3**
**Figure S2.** Protein residues Root Mean Square Fluctuation during the 50 nsec MD simulation showing local changes among the protein chain.	**4**
**Figure S3.** Structures of **8d** (**A**) in magenta and **8a** (**B**) in blue bound to M^{pro} in comparison with GK241 (**C**) in green and crystal structure PDB 6Y2F (**D**) in chaki. The side chain in derivative **8d** adopts a curved conformation exploring part of S1 and S2 protease cavities. Derivative **8a** aliphatic chain adopts a very similar structural arrangement to GK241, however the alanine moiety appears to interact weakly with N142 and T26.	**5**
**^{1}H NMR and ^{13}C NMR spectra of the compounds synthesized.**	**6**

Table S1. Structural details of structures 1-7 concerning the configuration of 2-carbon and the dihedral angle $C\beta_{C145}$-S_{C145}-C2-O2 related to the covalent bond formatted between Cys145 and 2-oxoamide moiety. Molecular Dynamics RMSDs for the protein and the ligand along with MM-GBSA Binding Energy.

Structure	Configuration of 2-carbon	MM-GBSA Binding Energy (kcal/mol)	Average Protein RMSD ±StDev	Average Ligand RMSD ±StDev	Major Interactions	Dihedral Angle Cβ-S-C2-O2
1	*R*	-58.69	2.84±0.48	3.36±0.95	S144	-73.68
2	*R*	-56.37	1.77±0.24	4.63±0.70	E166	-175.218
3	*S*	-54.51	1.73±0.28	7.36±3.73	-	86.525
4	*S*	-47.03	2.29±0.35	5.53±0.89	T26, T24	-45.037
5	*R*	-46.41	2.00±0.32	3.97±1.10	H41, E166	-50.033
6	*S*	-45.17	1.95±0.31	5.05±0.95	H41, N142, H164	134.808
7	*S*	-43.26	1.79±0.26	3.22±0.79	T26, H41, N142	-55.195

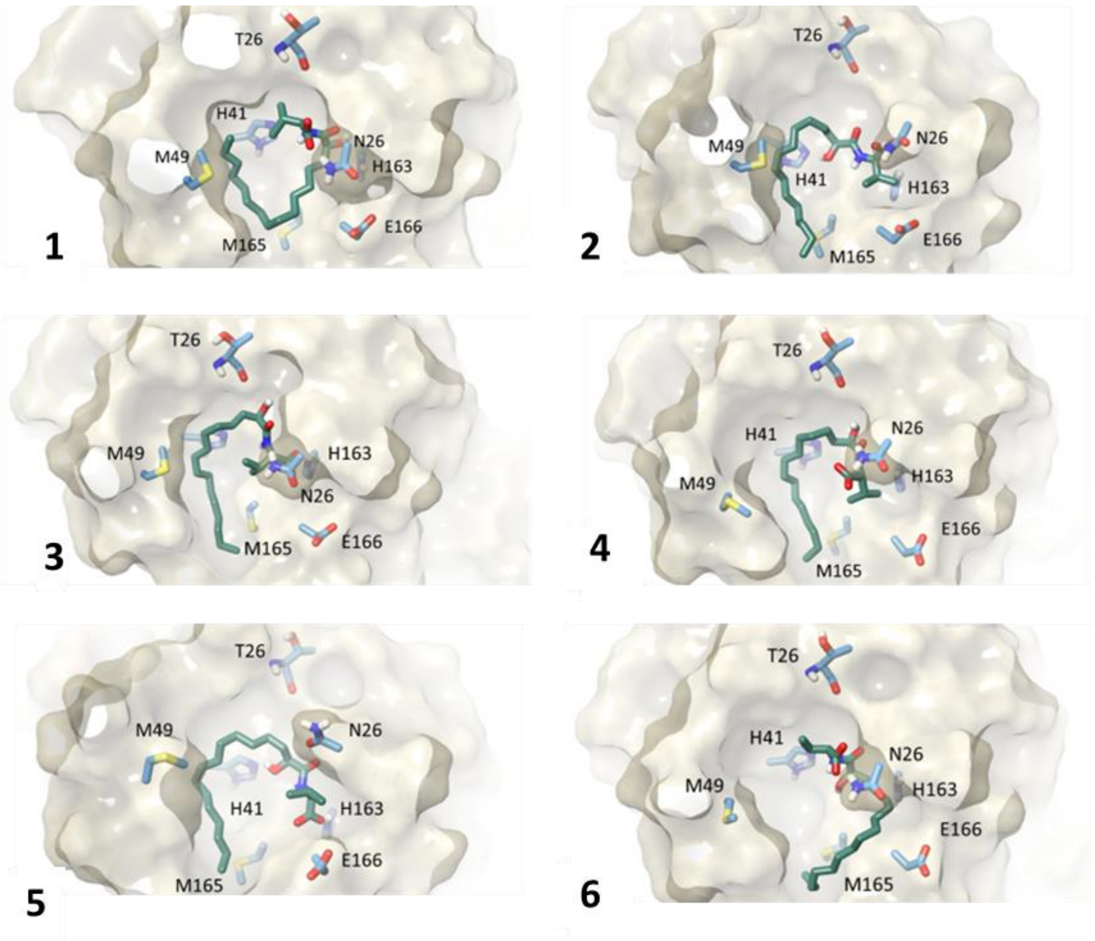

Figure S1. Structural details for the M^{pro}-GK241 interactions in structures 1-6 resulted by covalent docking calculations.

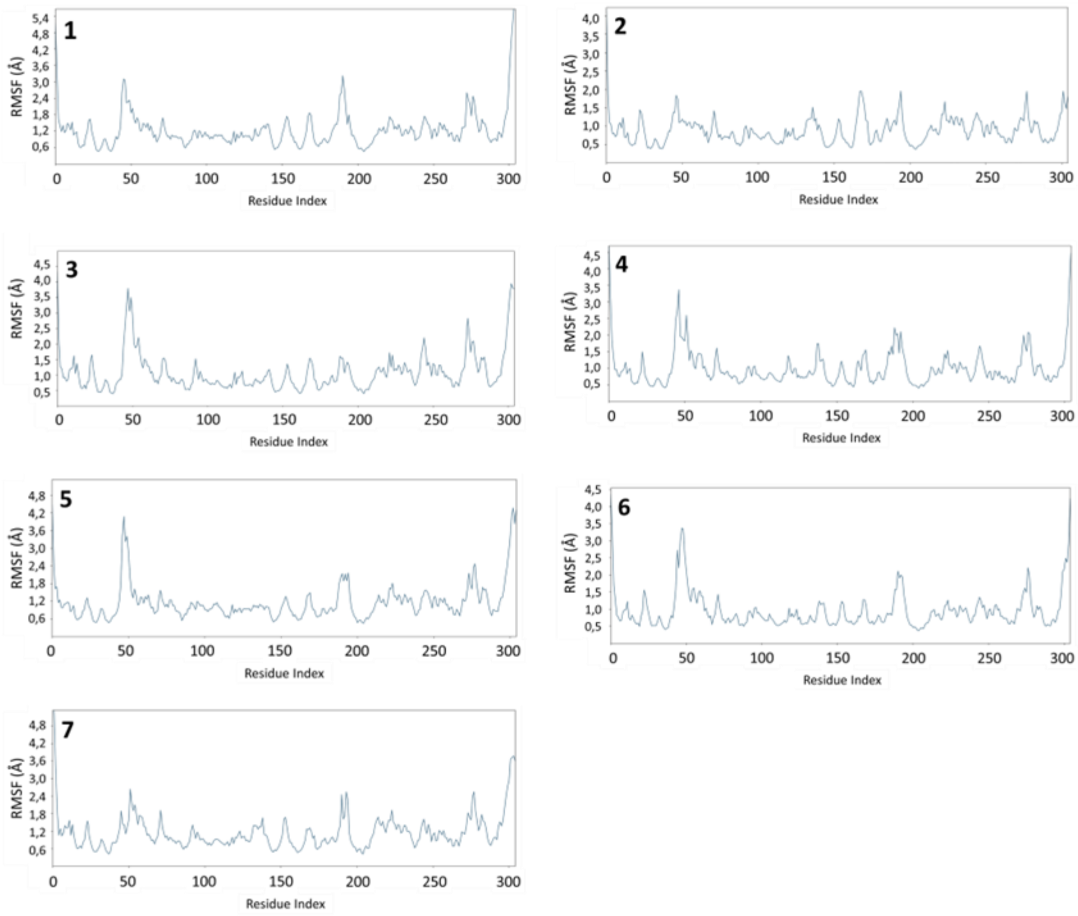

Figure S2. Protein residues Root Mean Square Fluctuation during the 50 nsec MD simulation showing local changes among the protein chain.

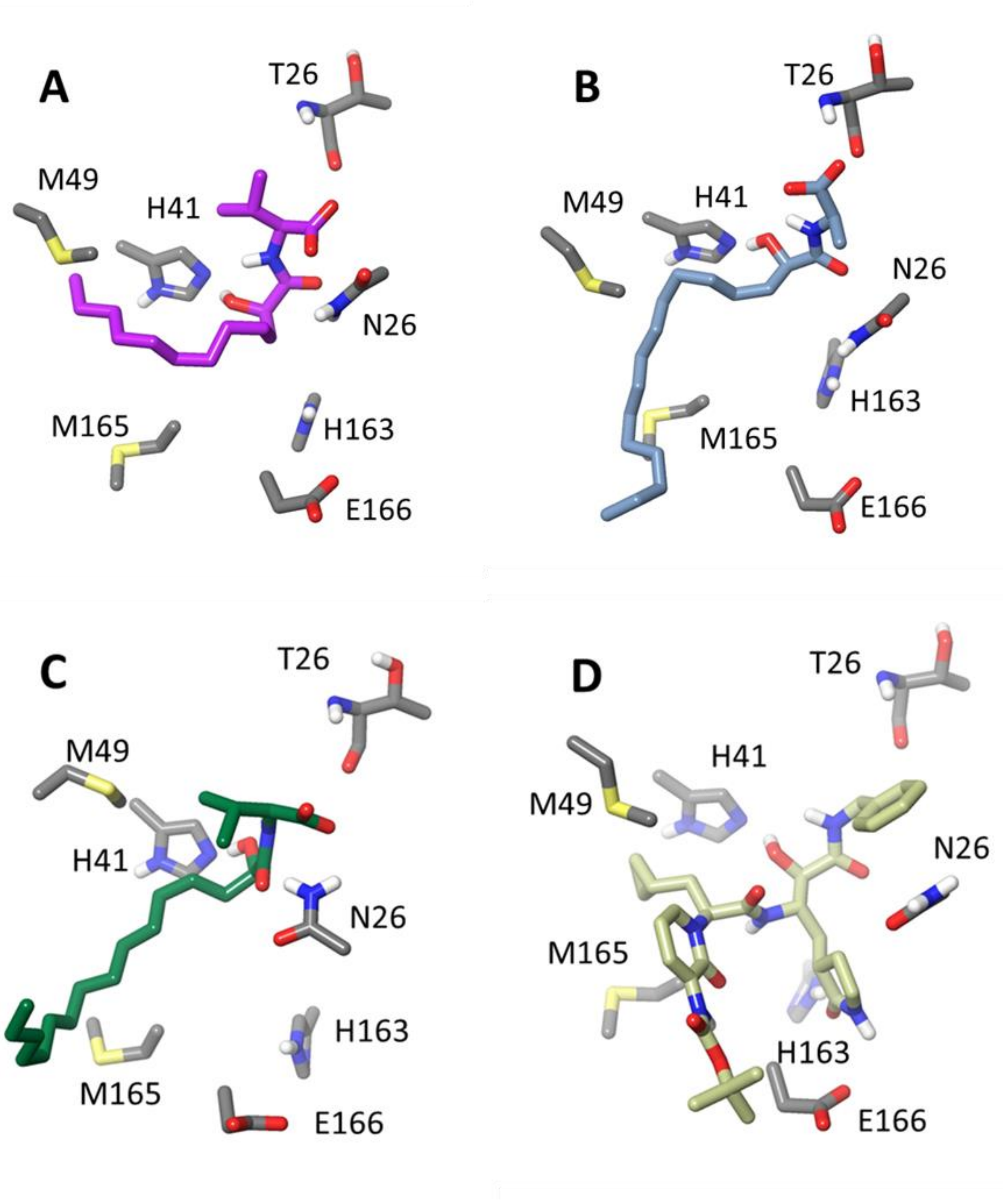

Figure S3. Structures of **8d** (**A**) in magenta and **8a** (**B**) in blue bound to M^{pro} in comparison with GK241 (**C**) in green and crystal structure PDB 6Y2F (**D**) in chaki. The side chain in derivative **8d** adopts a curved conformation exploring part of S1 and S2 protease cavities. Derivative **8a** aliphatic chain adopts a very similar structural arrangement to GK241, however the alanine moiety appears to interact weakly with N142 and T26.

NMR Spectra

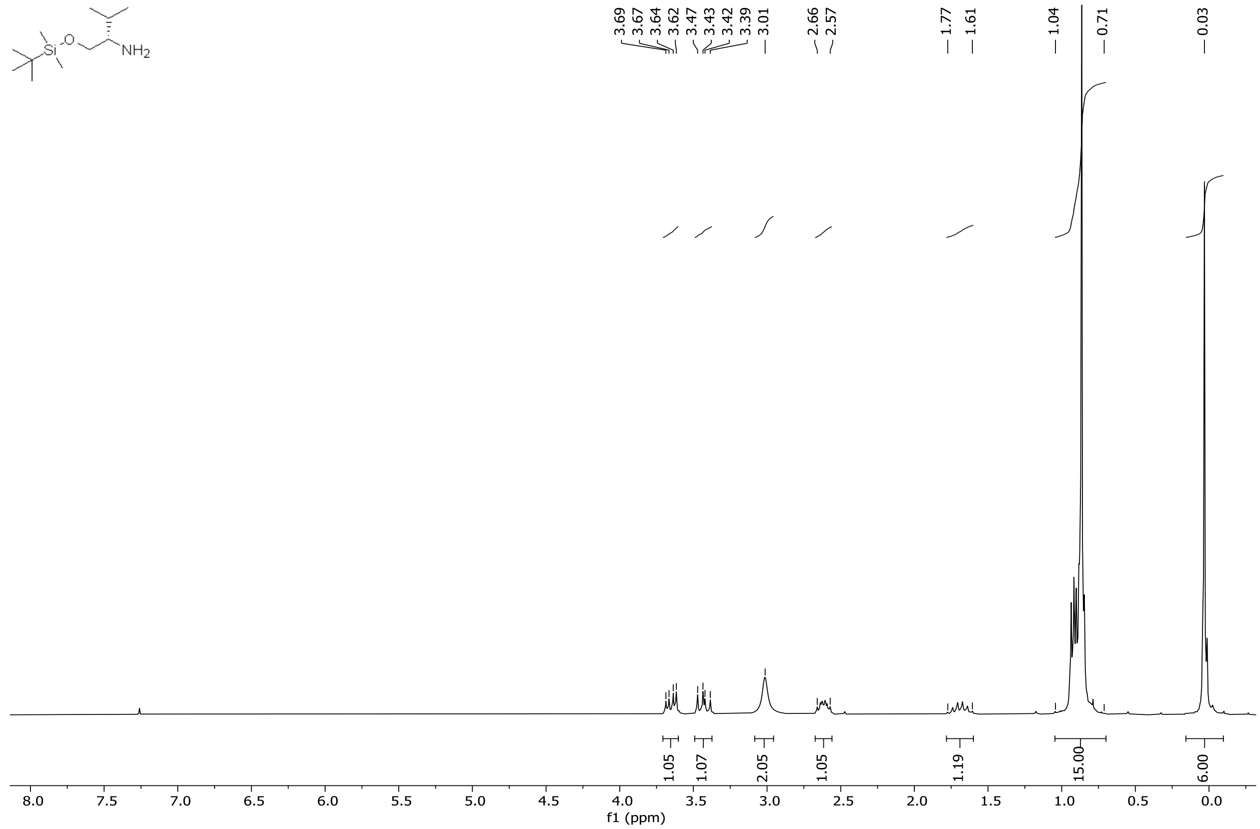

^{1}H NMR spectrum of **5b**.

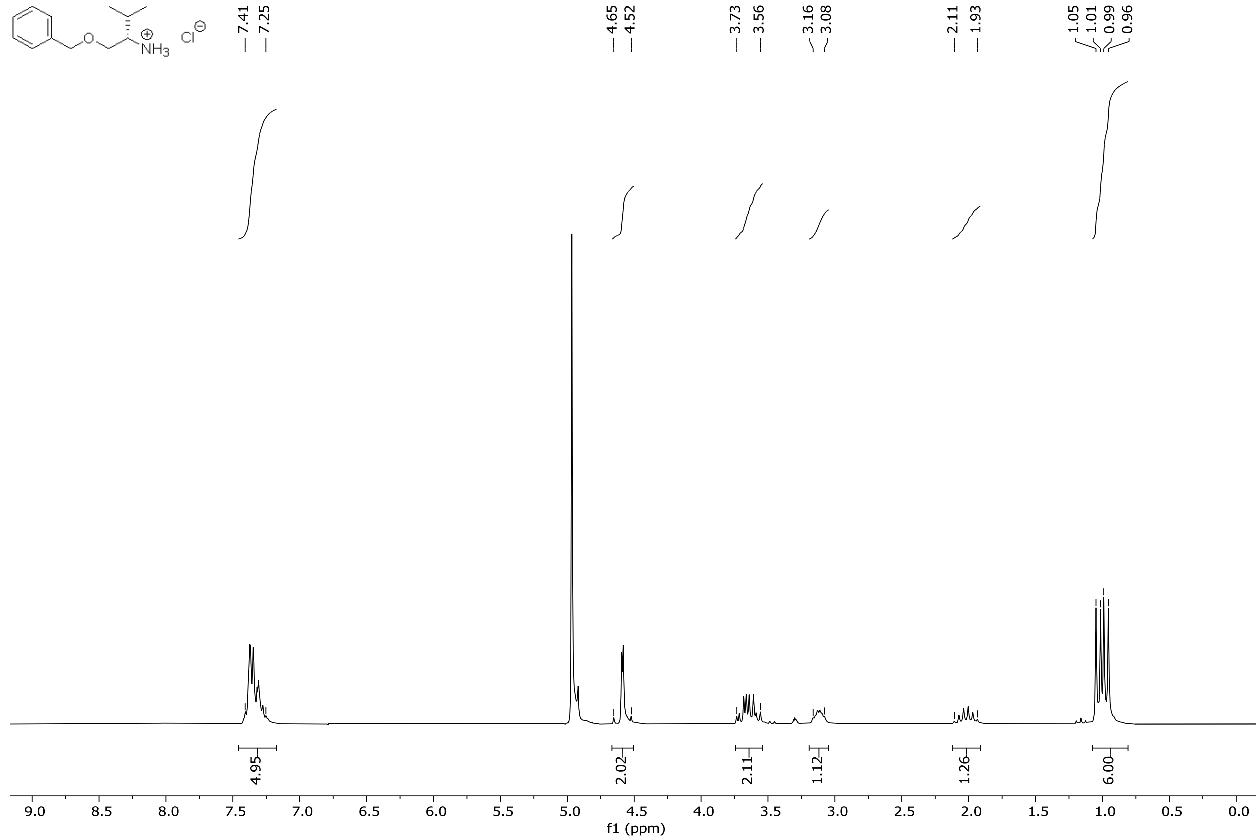

^{1}H NMR spectrum of **5c**.

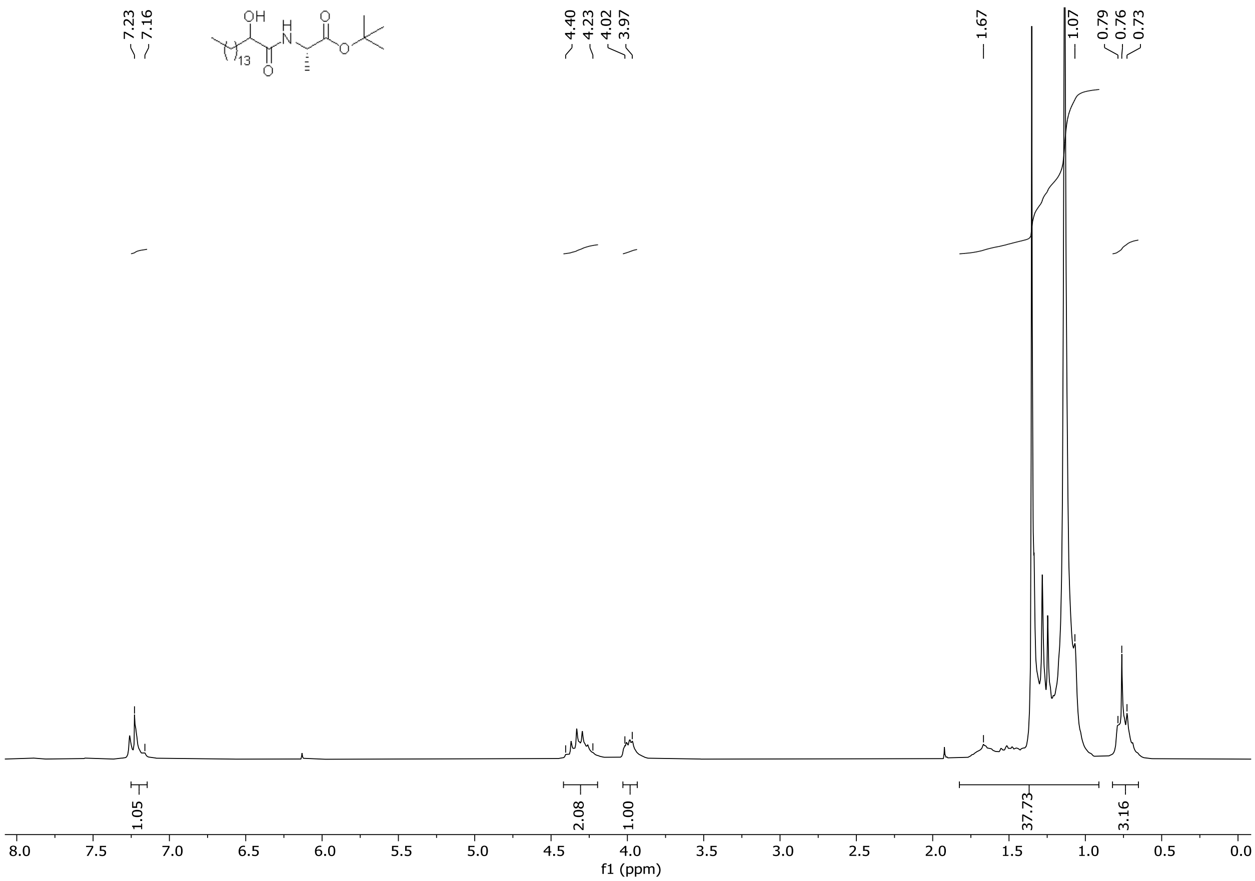

^{1}H NMR spectrum of **6a**, Diastereoisomer 1.

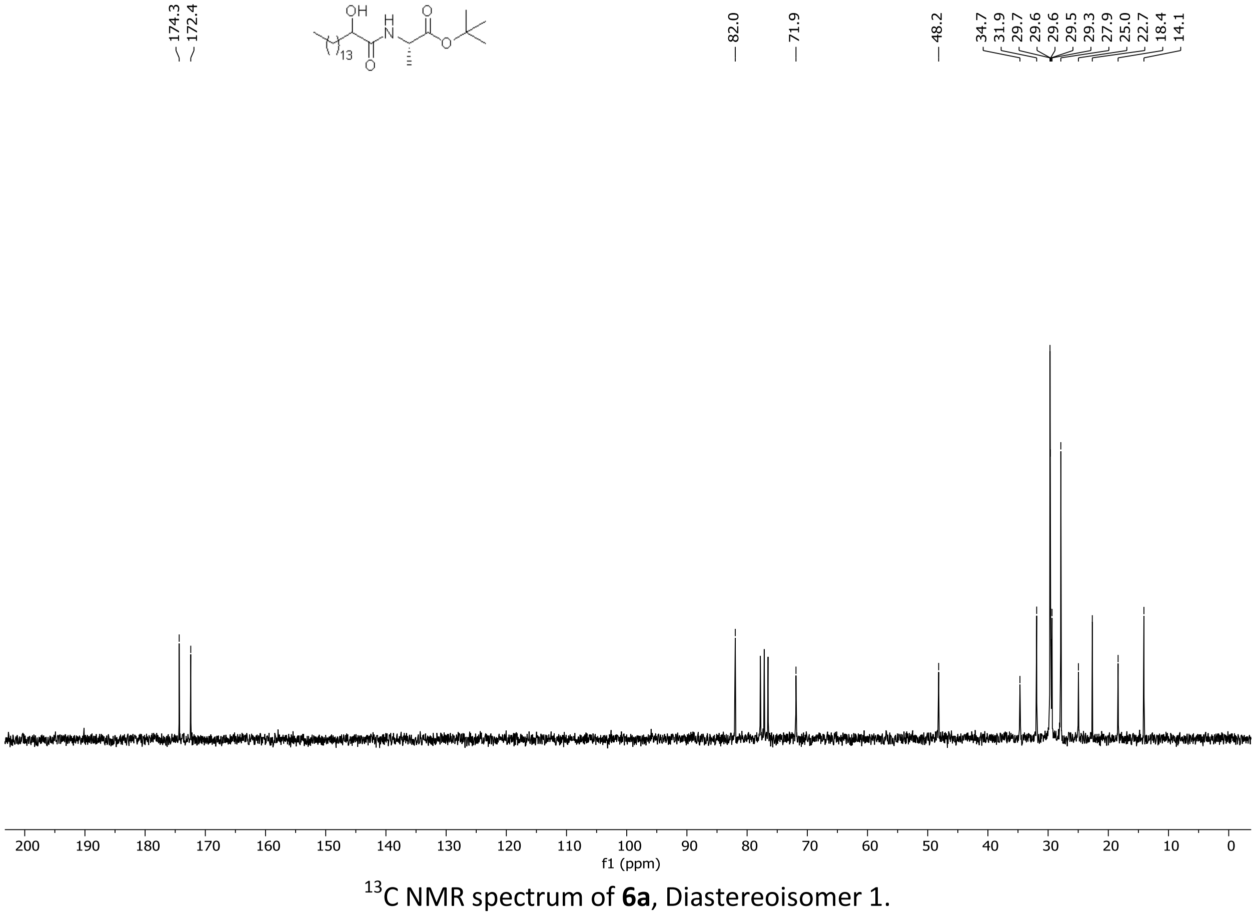

^{13}C NMR spectrum of **6a**, Diastereoisomer 1.

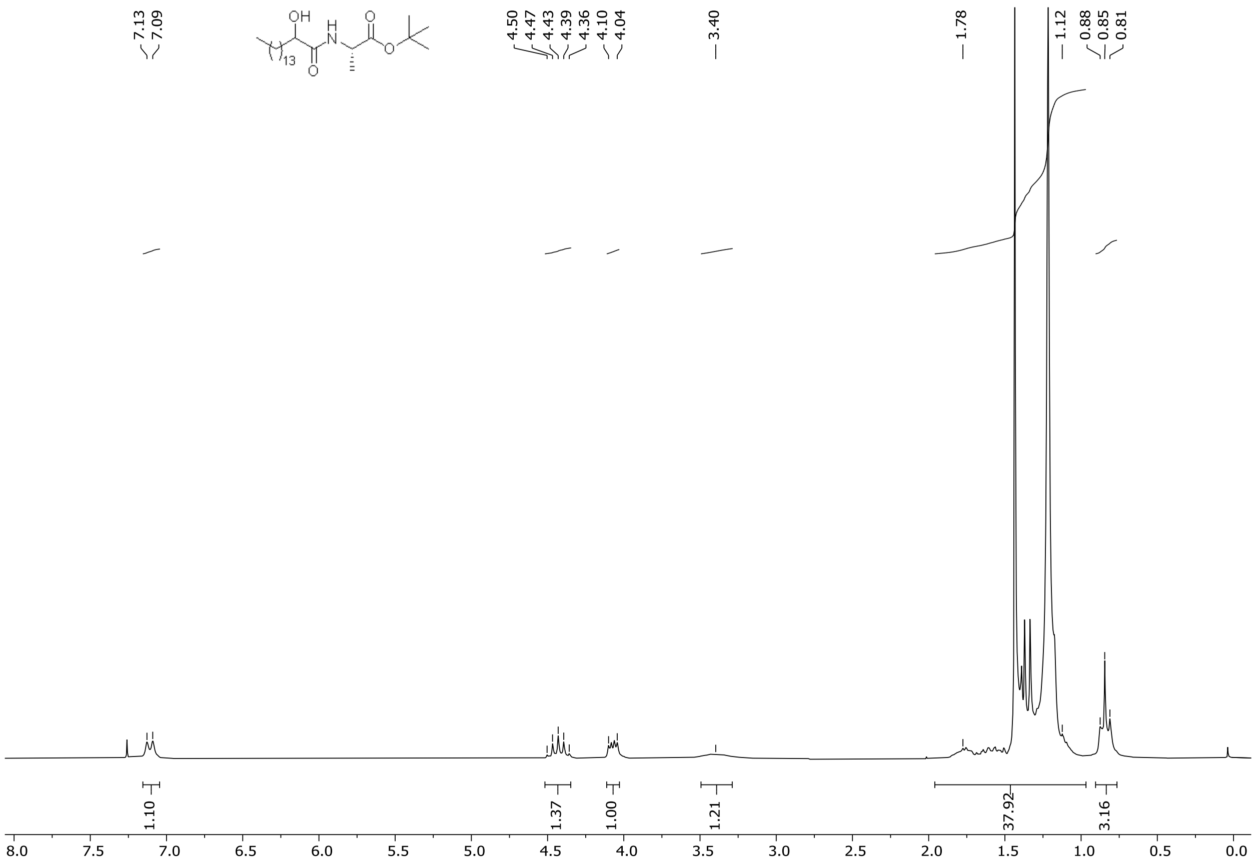

^{1}H NMR spectrum of **6a**, Diastereoisomer 2.

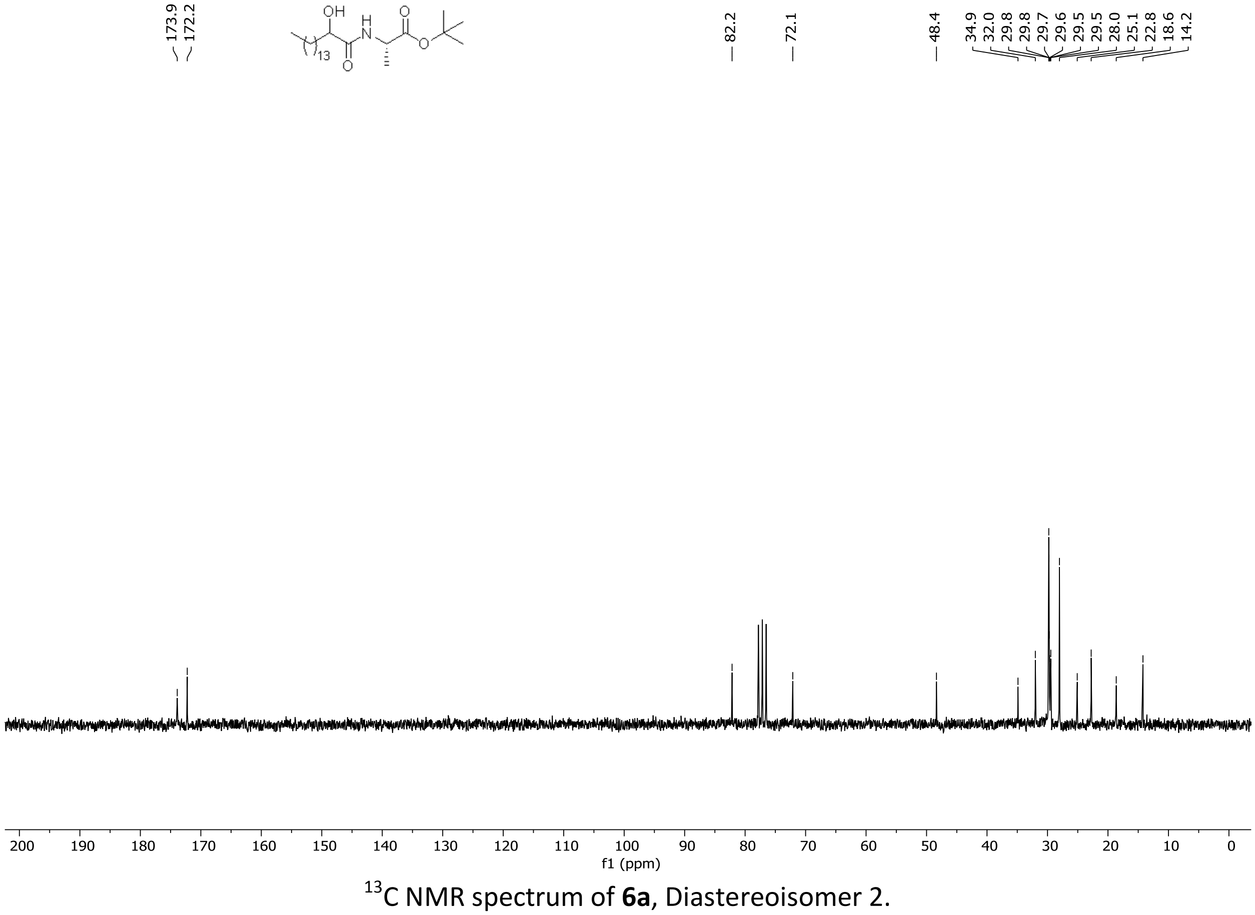

^{13}C NMR spectrum of **6a**, Diastereoisomer 2.

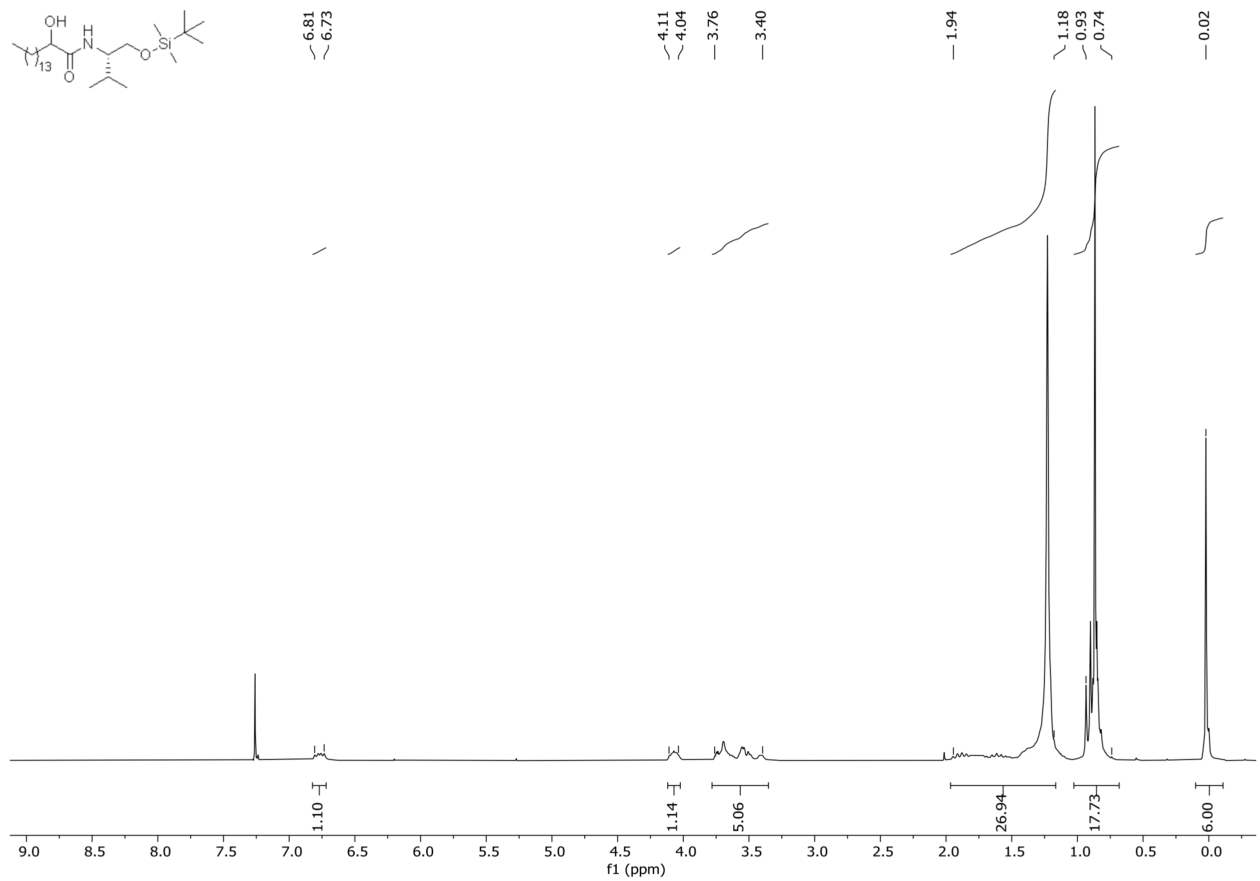

^{1}H NMR spectrum of **6b**.

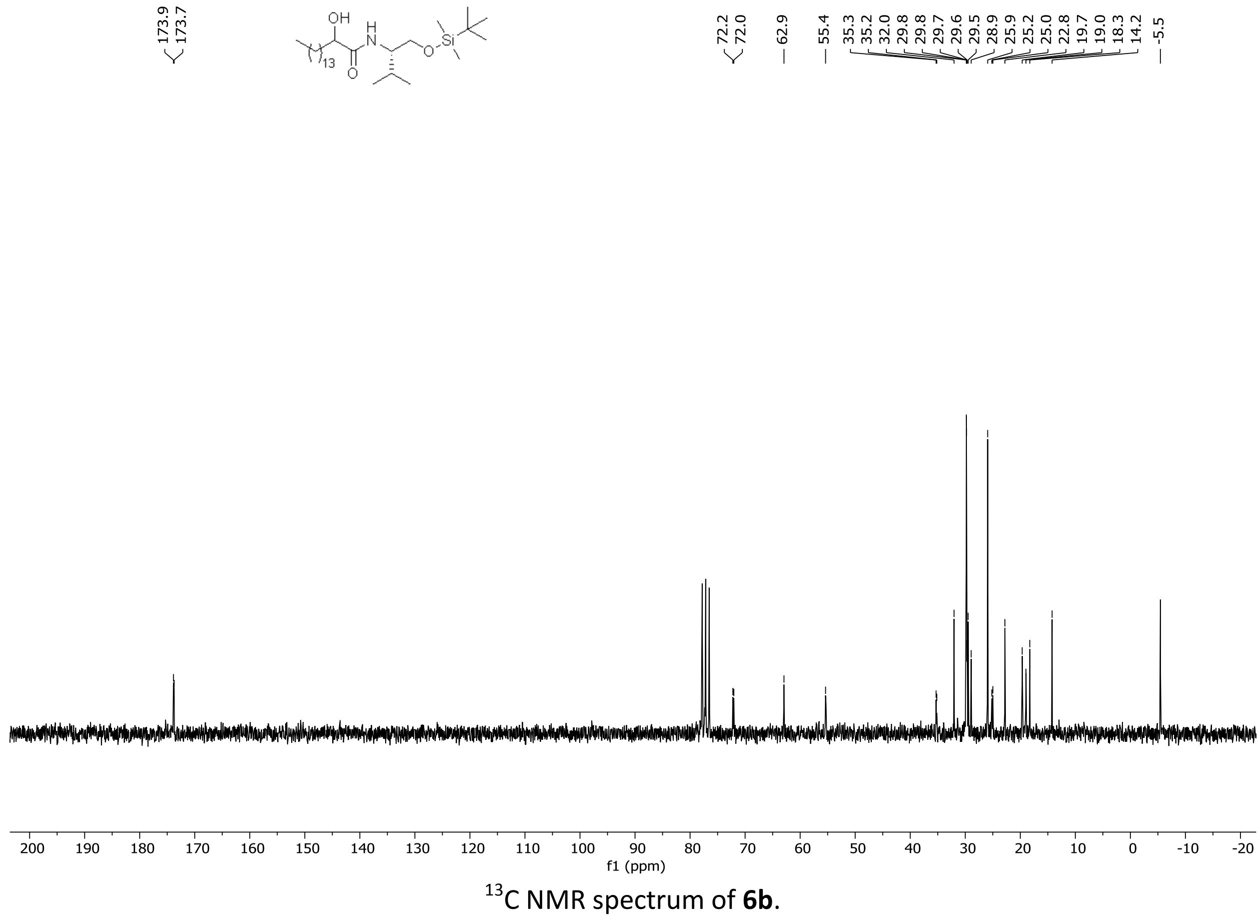

^{13}C NMR spectrum of **6b**.

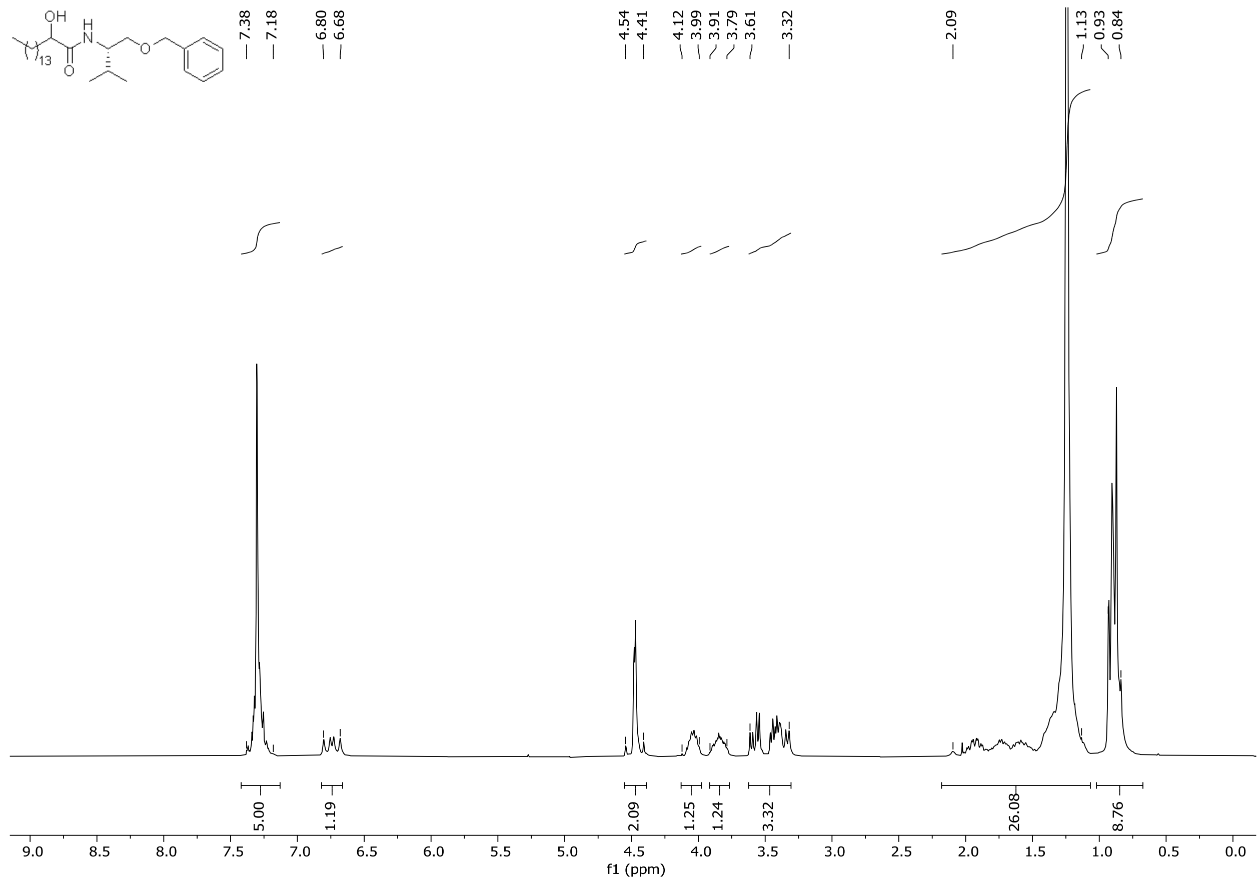

^{1}H NMR spectrum of **6c**.

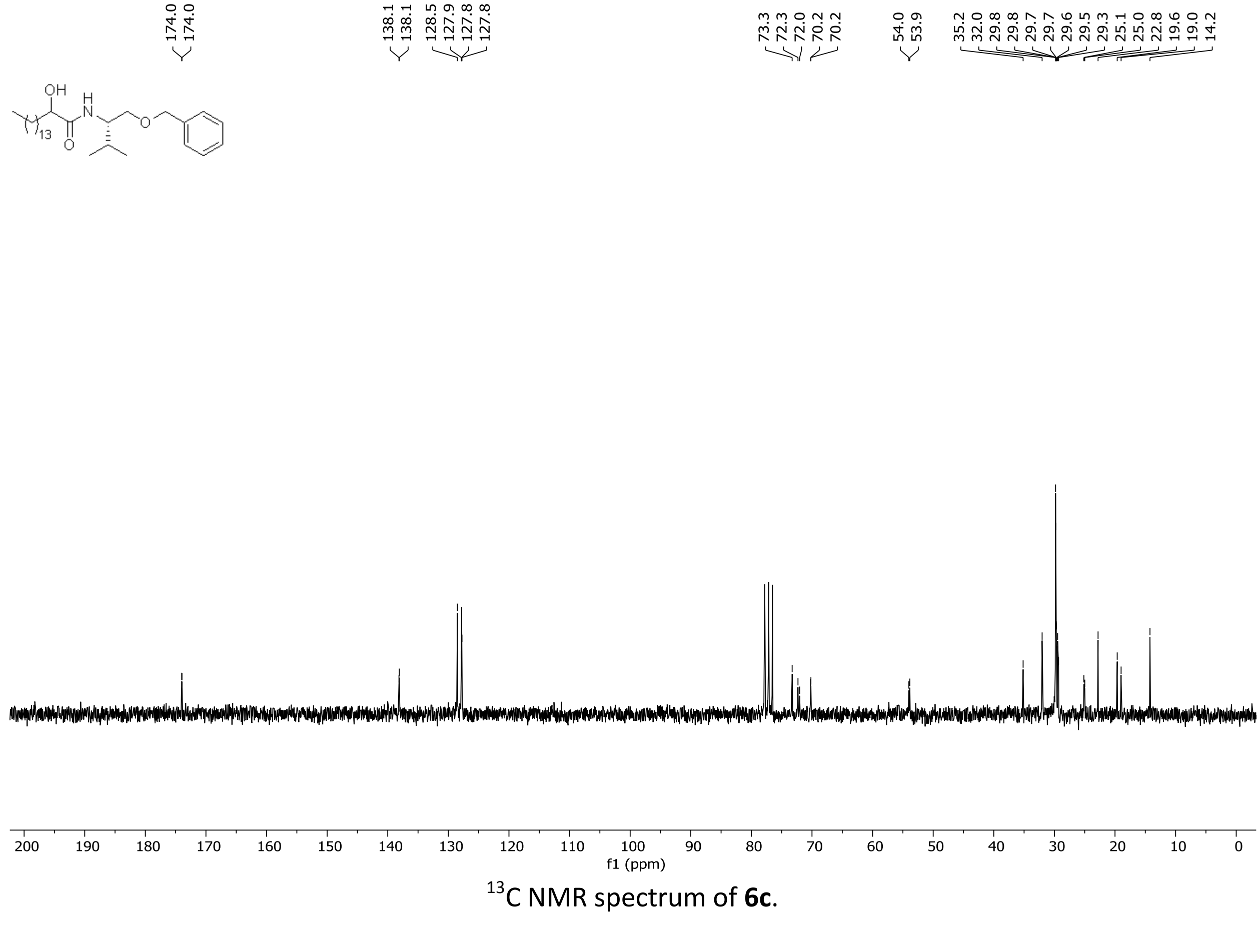

^{13}C NMR spectrum of **6c**.

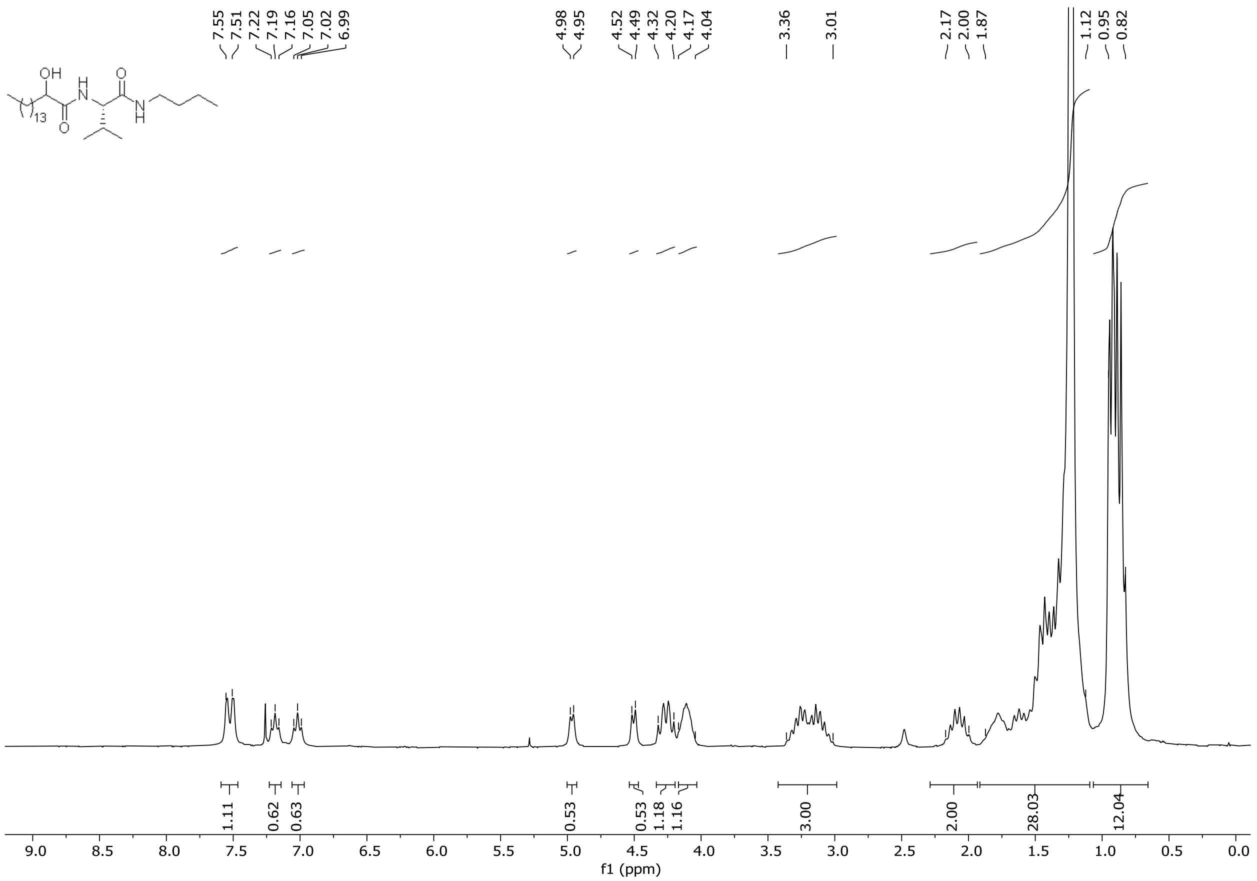

^{1}H NMR spectrum of **6d**.

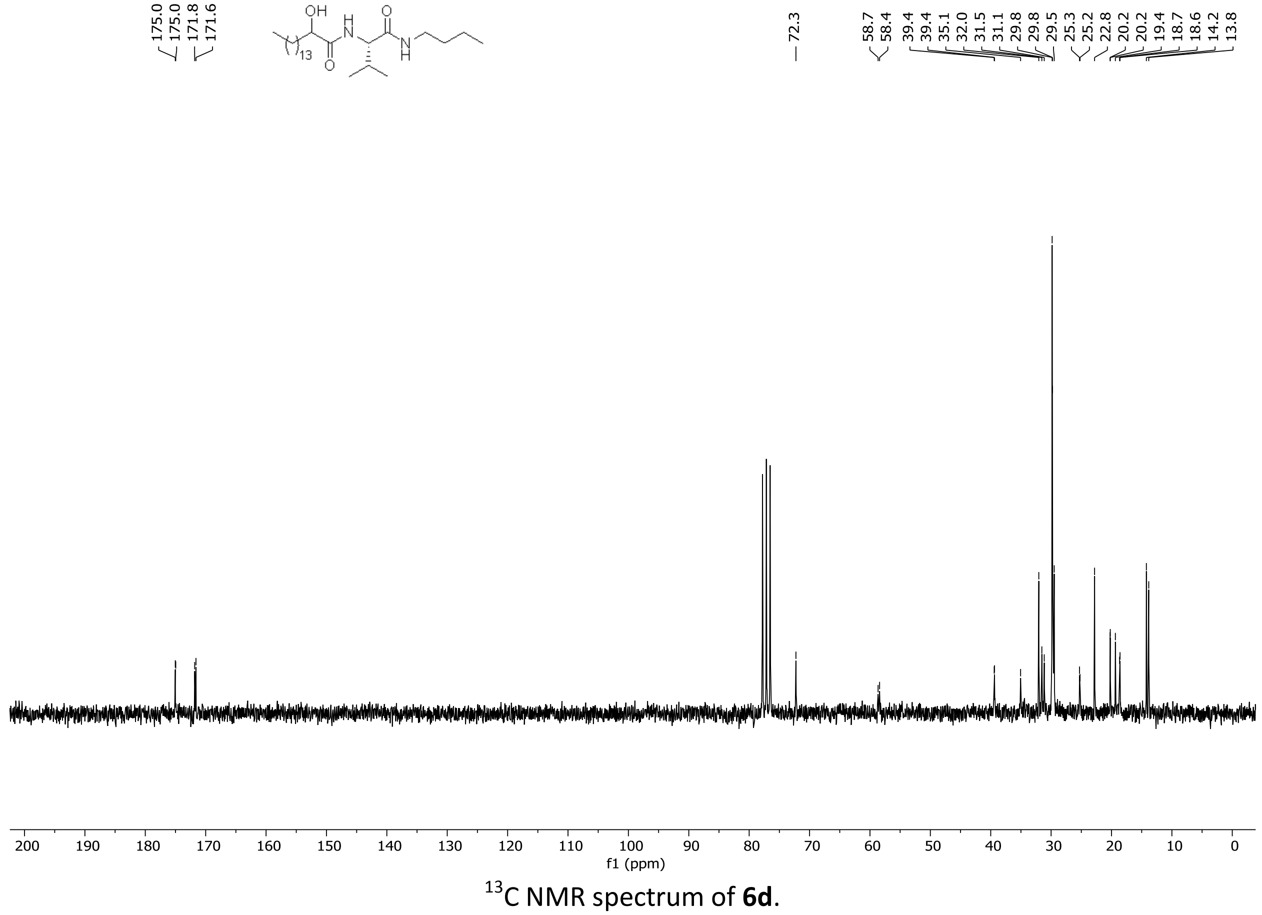

^{13}C NMR spectrum of **6d**.

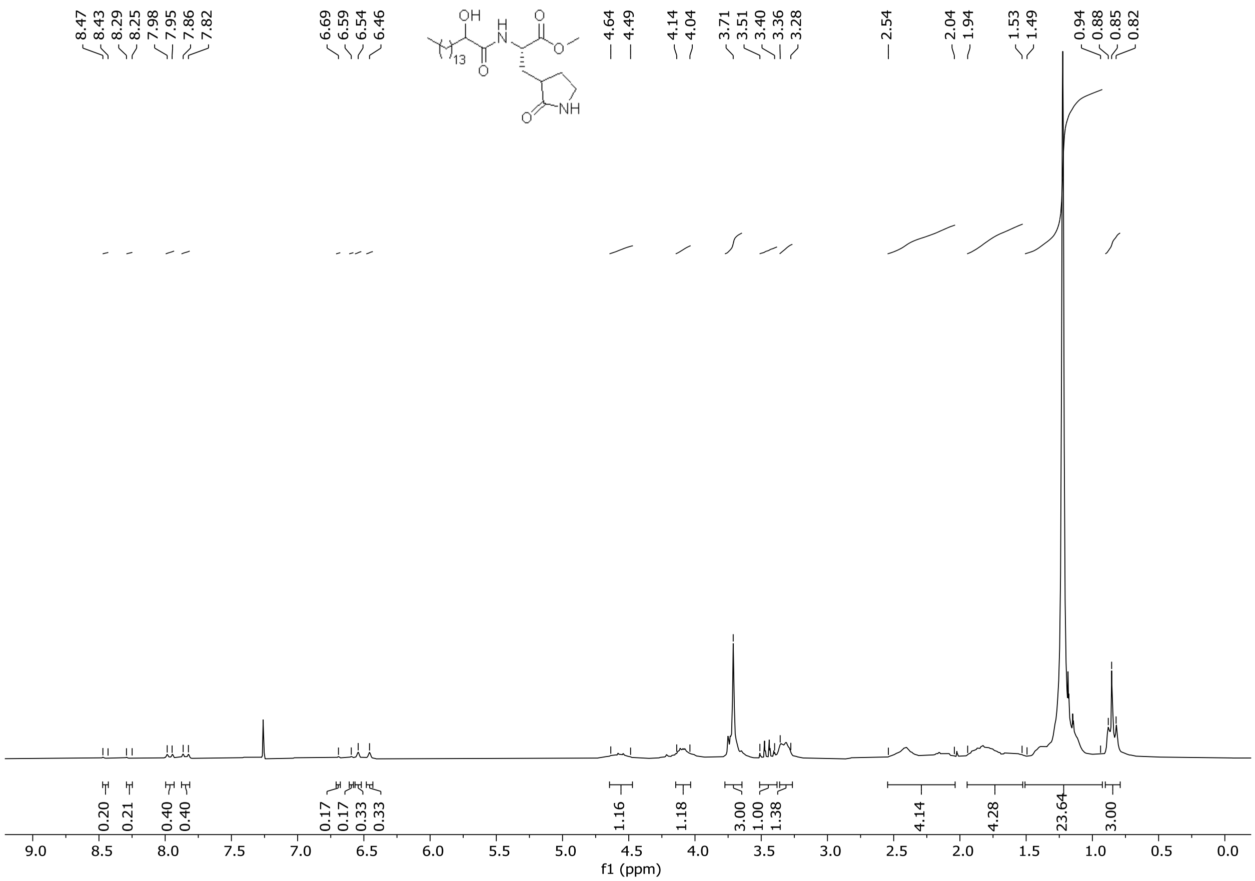

^{1}H NMR spectrum of **6e**.

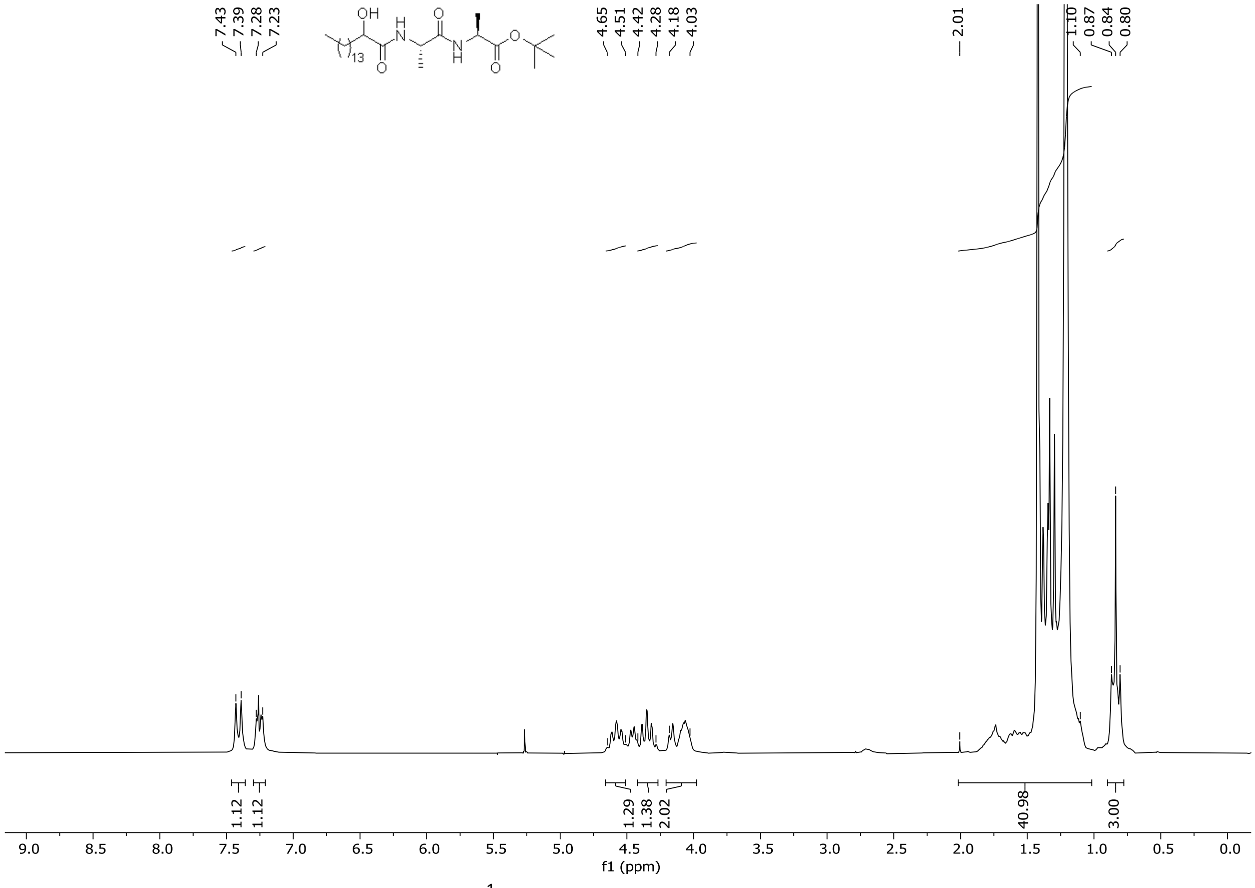

^{1}H NMR spectrum of **6f**.

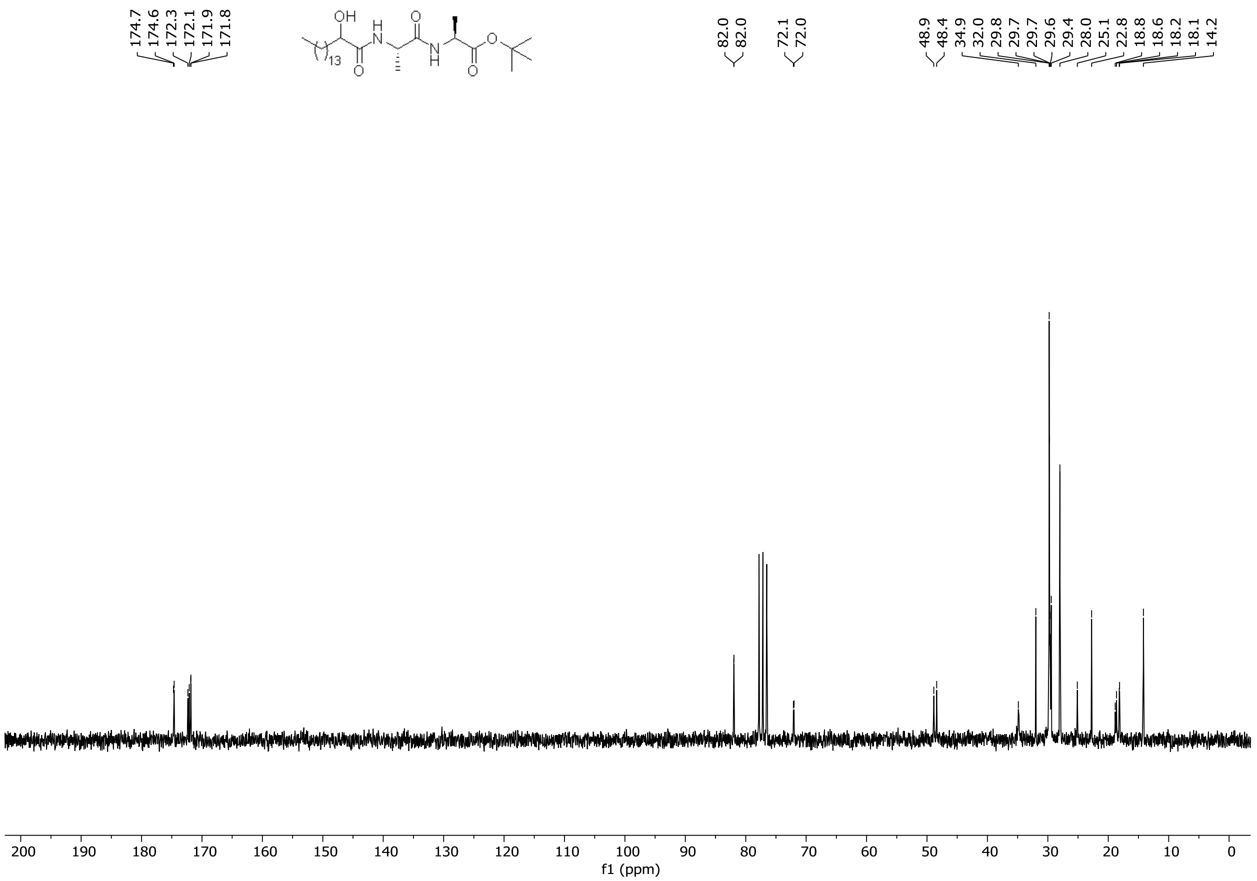

^{13}C NMR spectrum of **6f**.

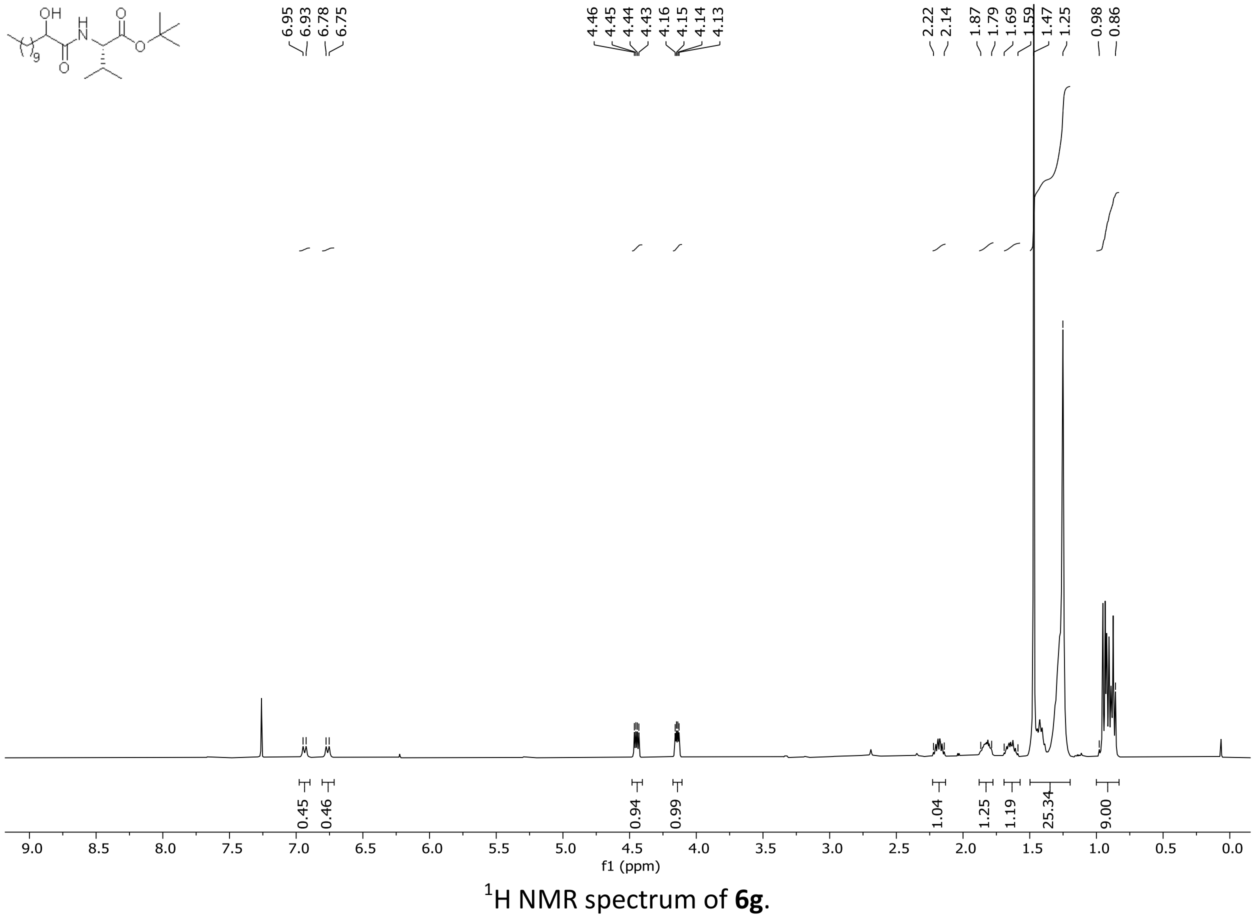

^{1}H NMR spectrum of **6g**.

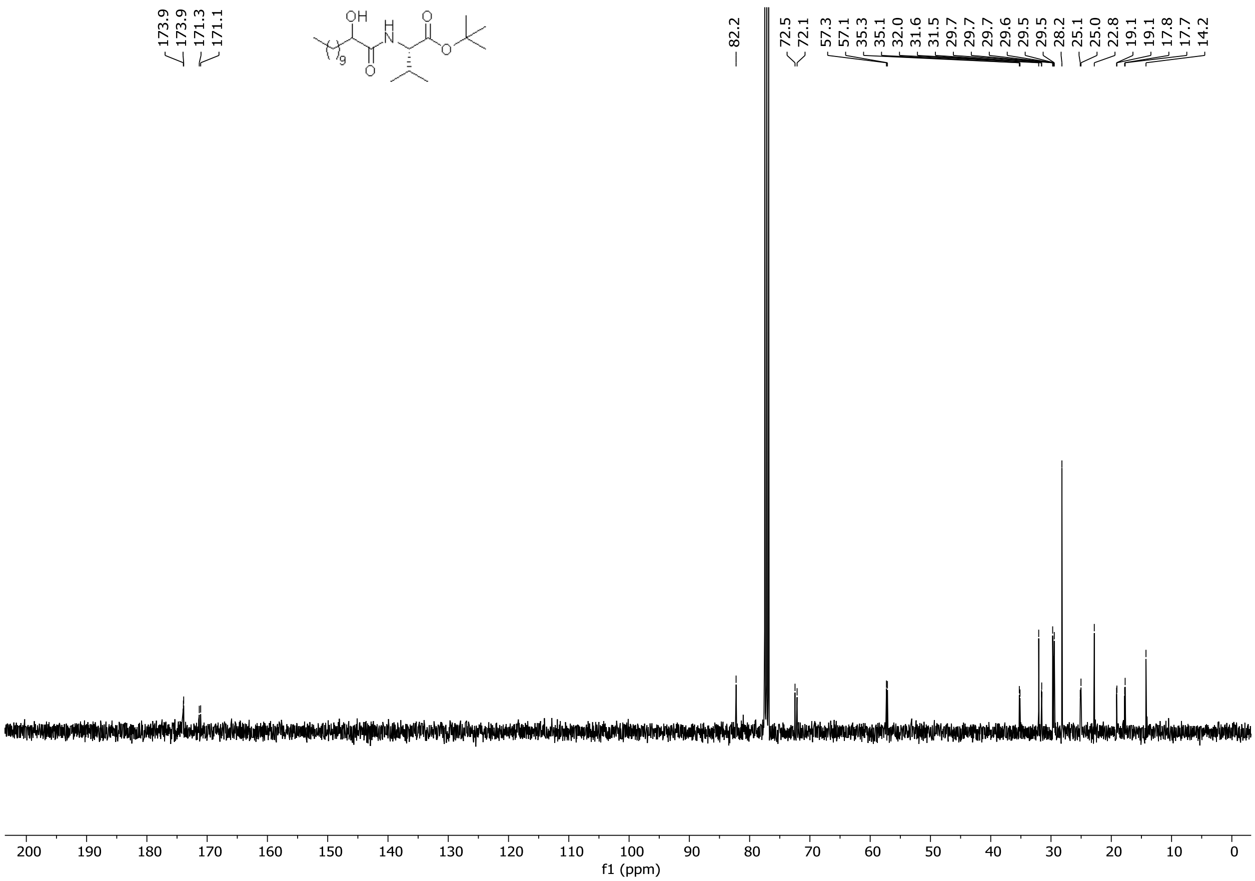

^{13}C NMR spectrum of **6g**.

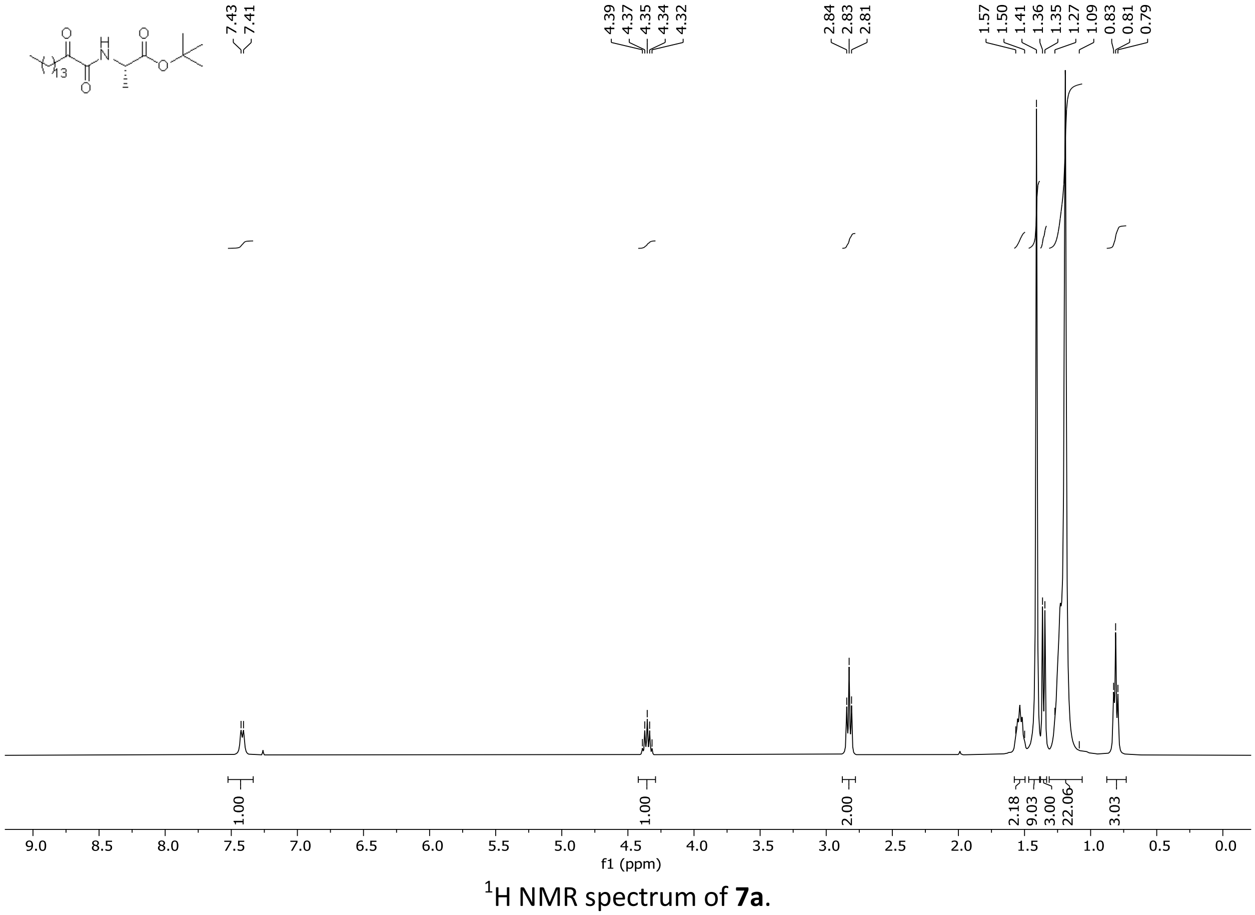

^{1}H NMR spectrum of **7a**.

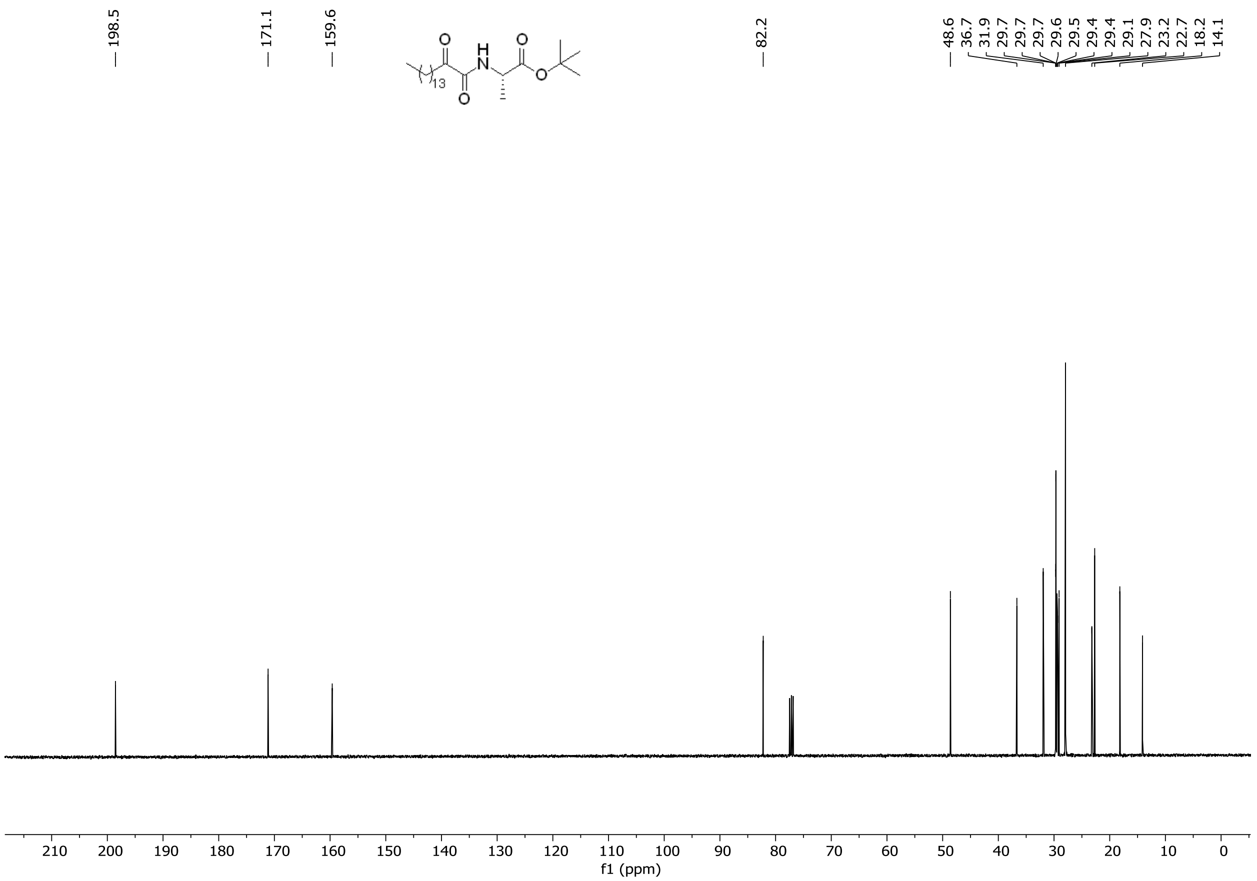

^{13}C NMR spectrum of **7a**.

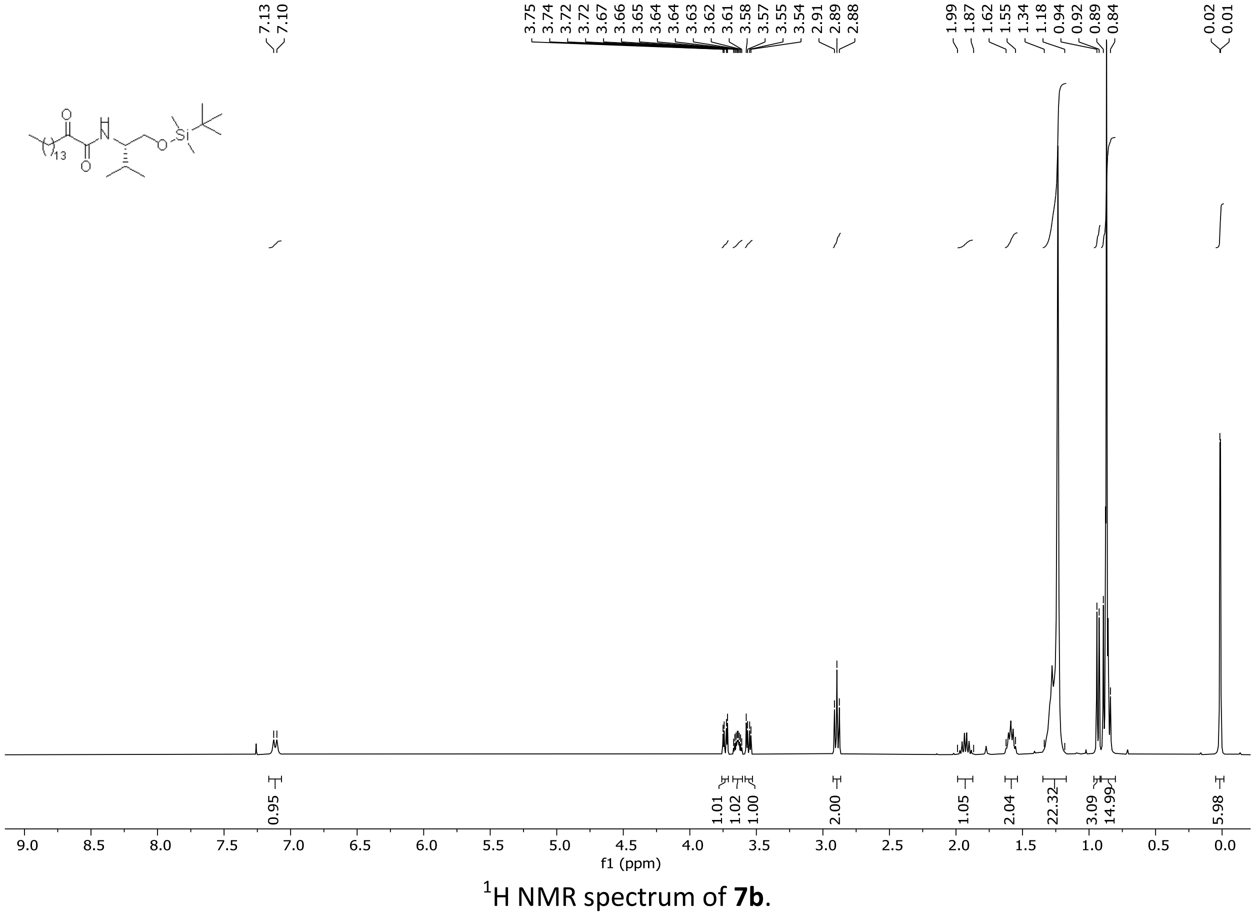

^{1}H NMR spectrum of **7b**.

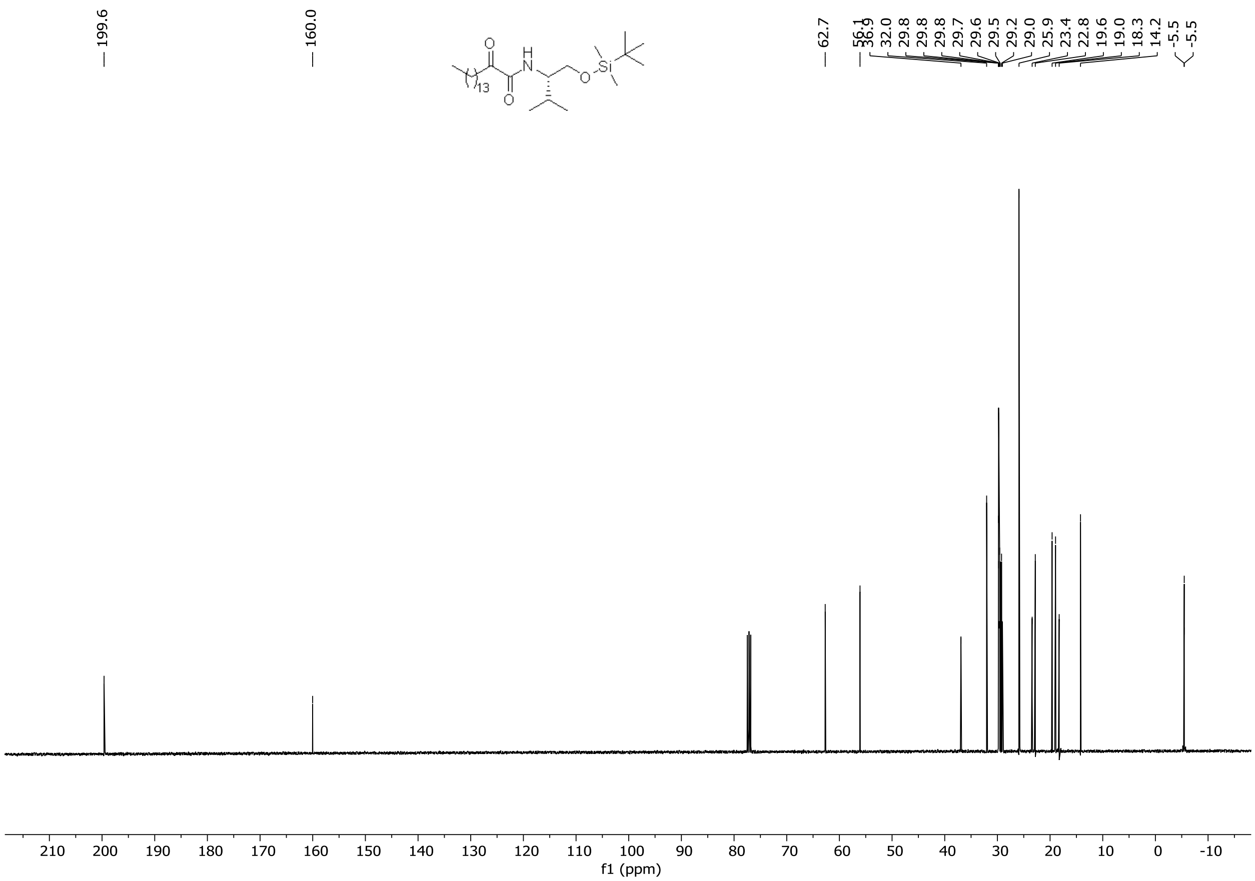

^{13}C NMR spectrum of **7b**.

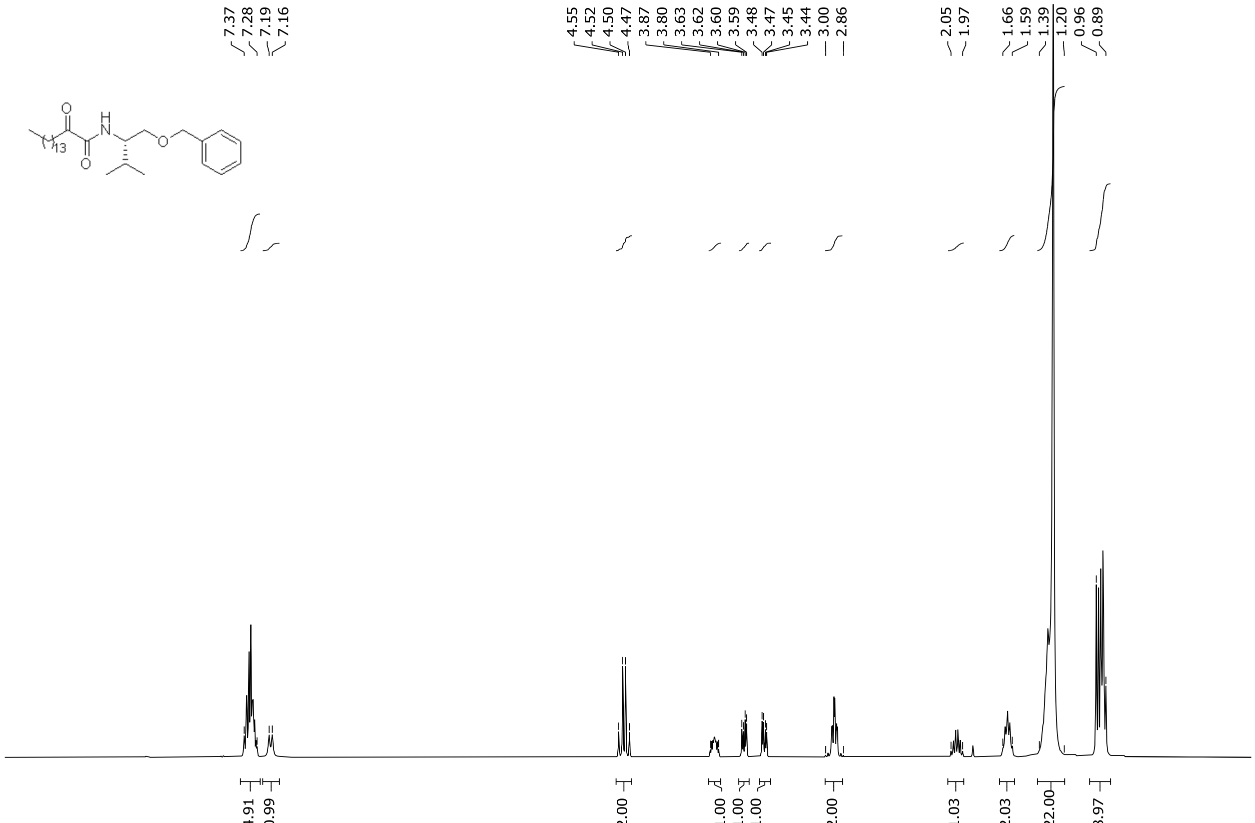

^{1}H NMR spectrum of **7c**.

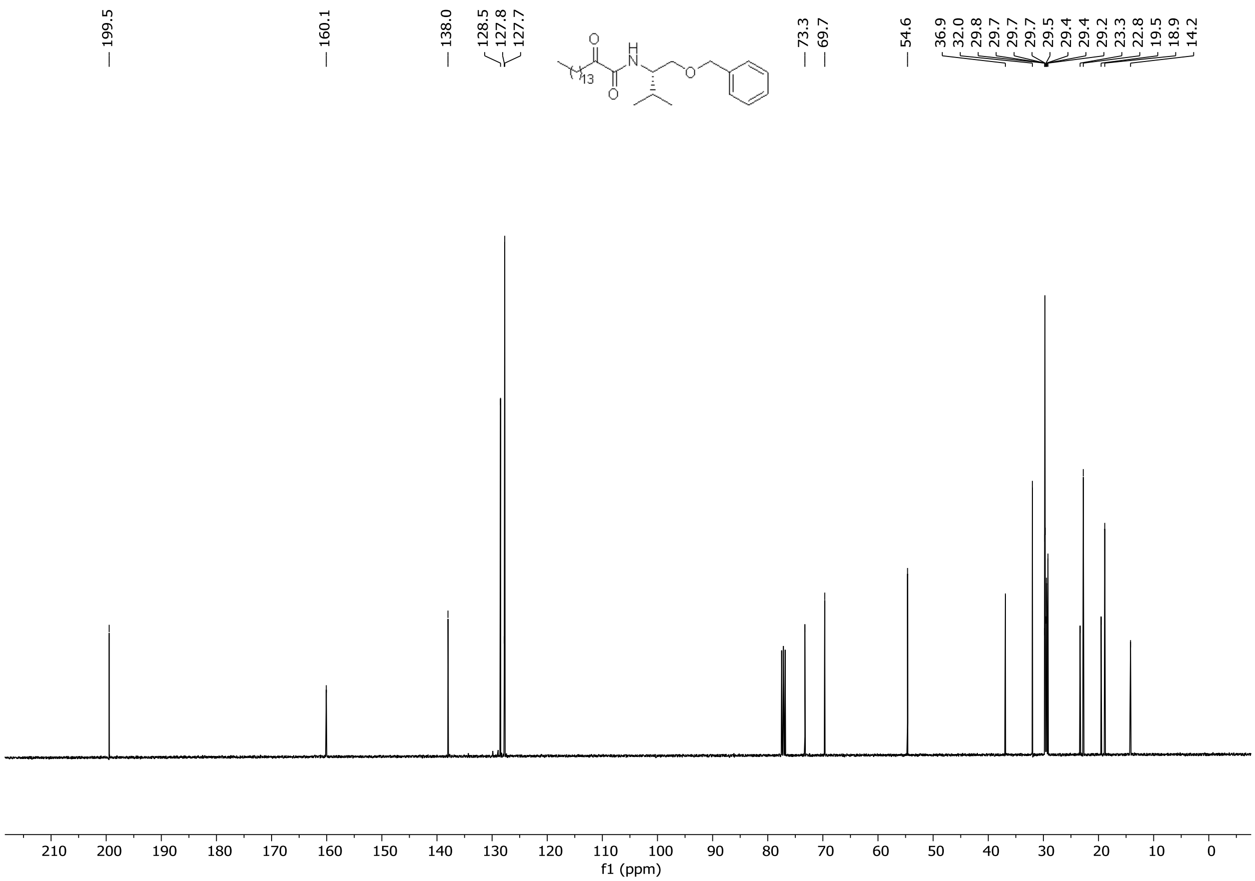

^{13}C NMR spectrum of **7c**.

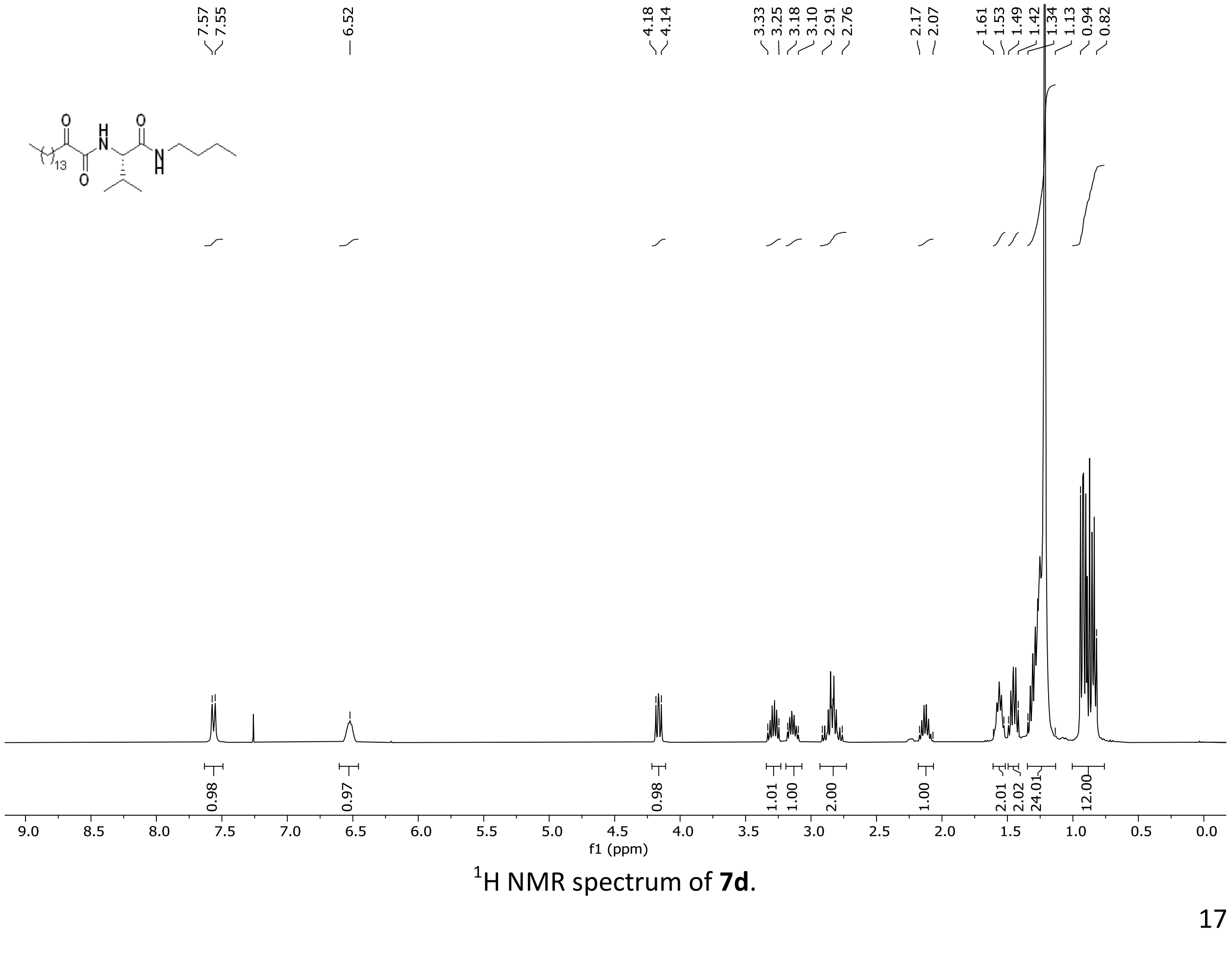

^{1}H NMR spectrum of **7d**.

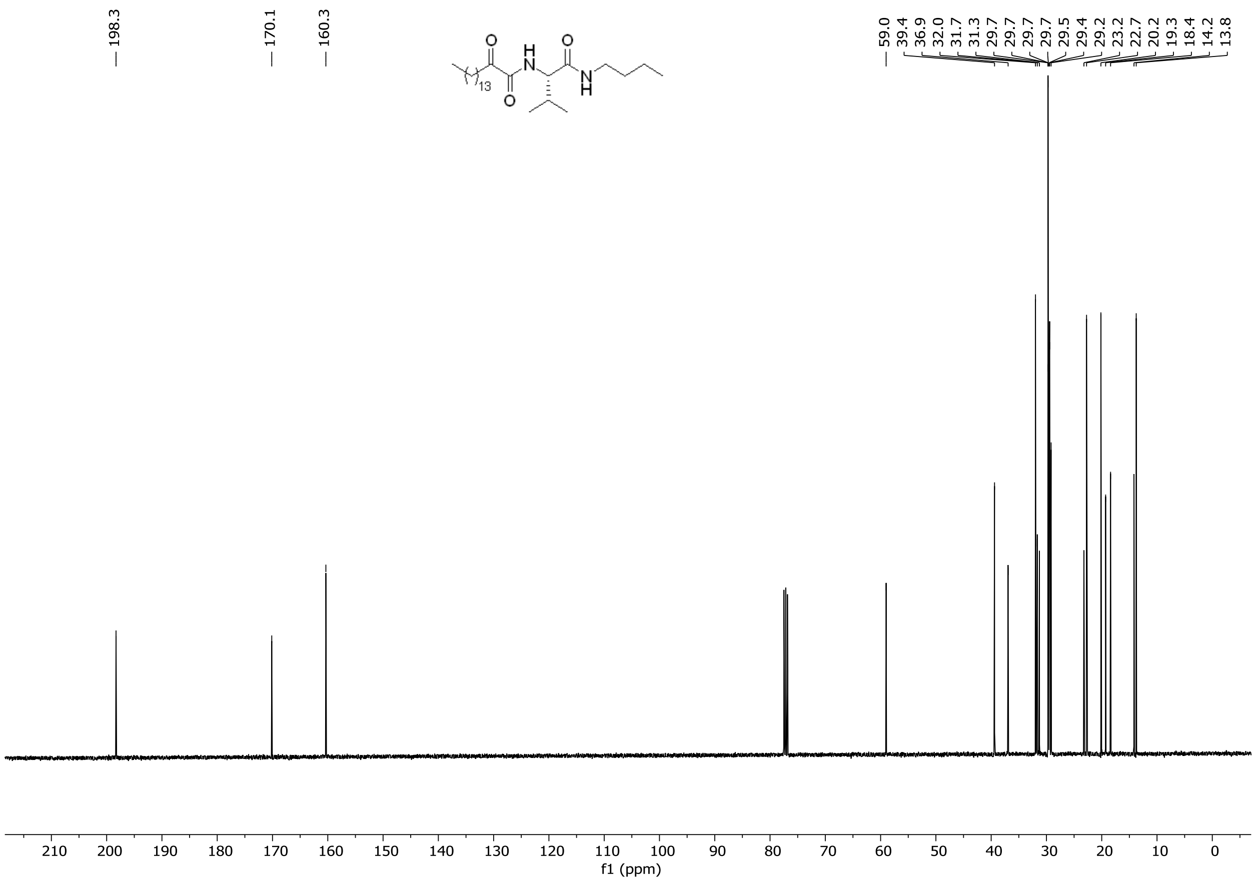

^{13}C NMR spectrum of **7d**.

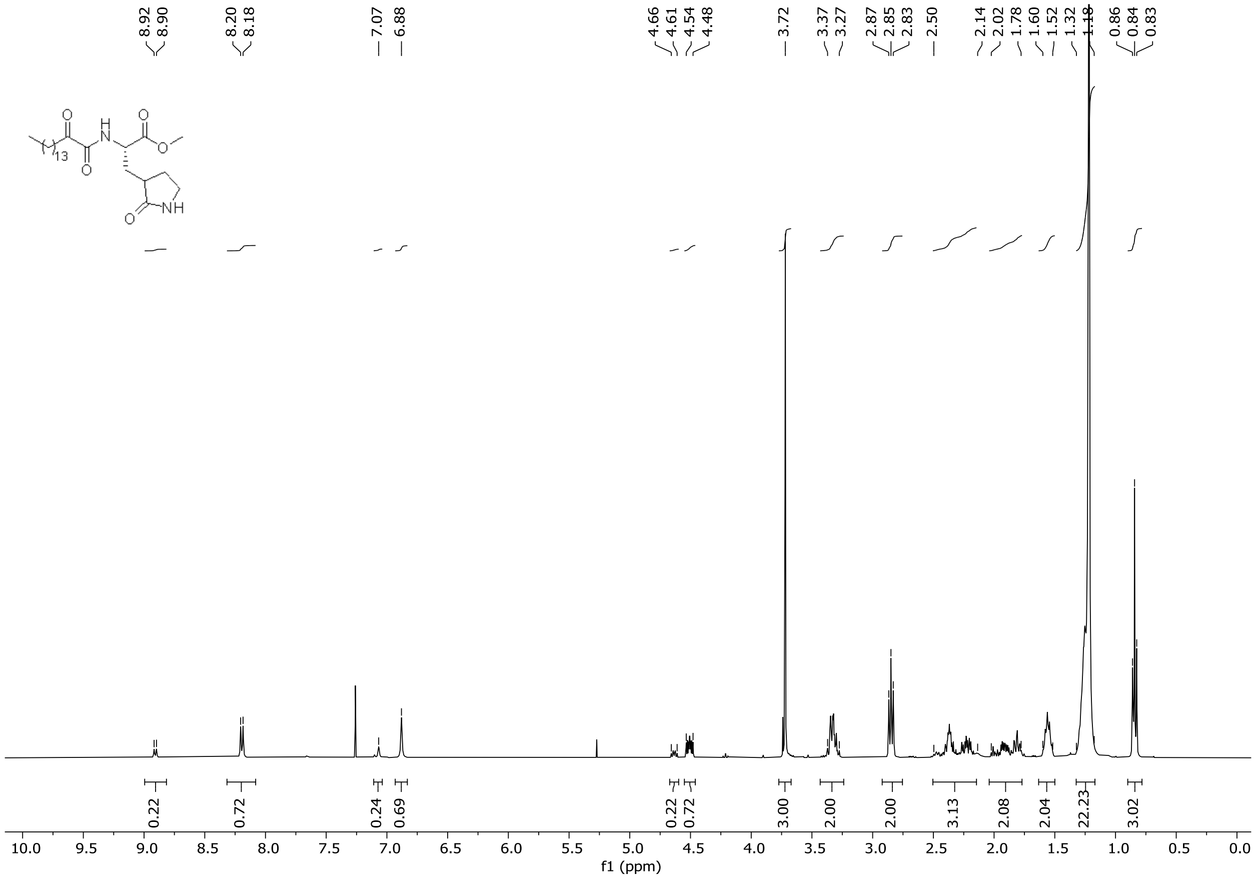

^{1}H NMR spectrum of **7e**.

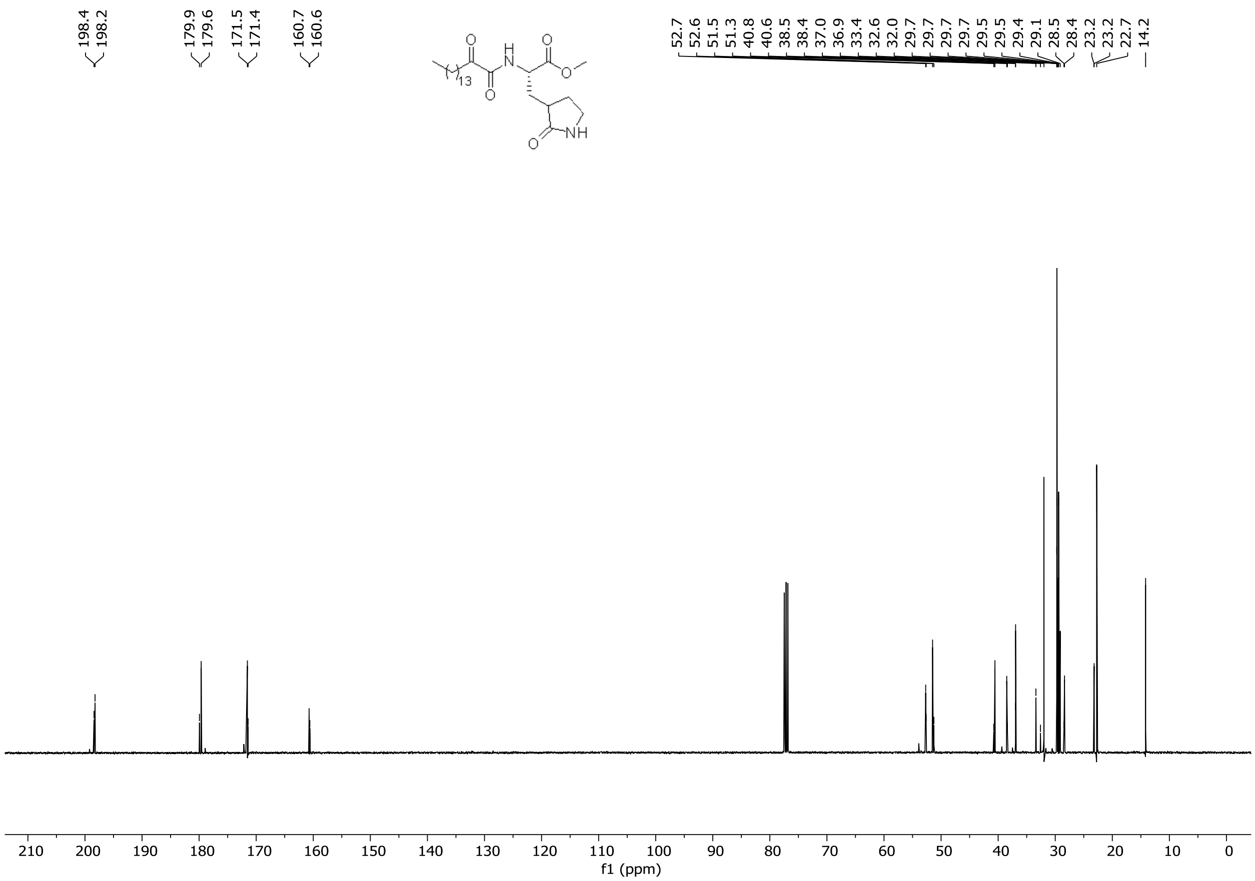

^{13}C NMR spectrum of **7e**.

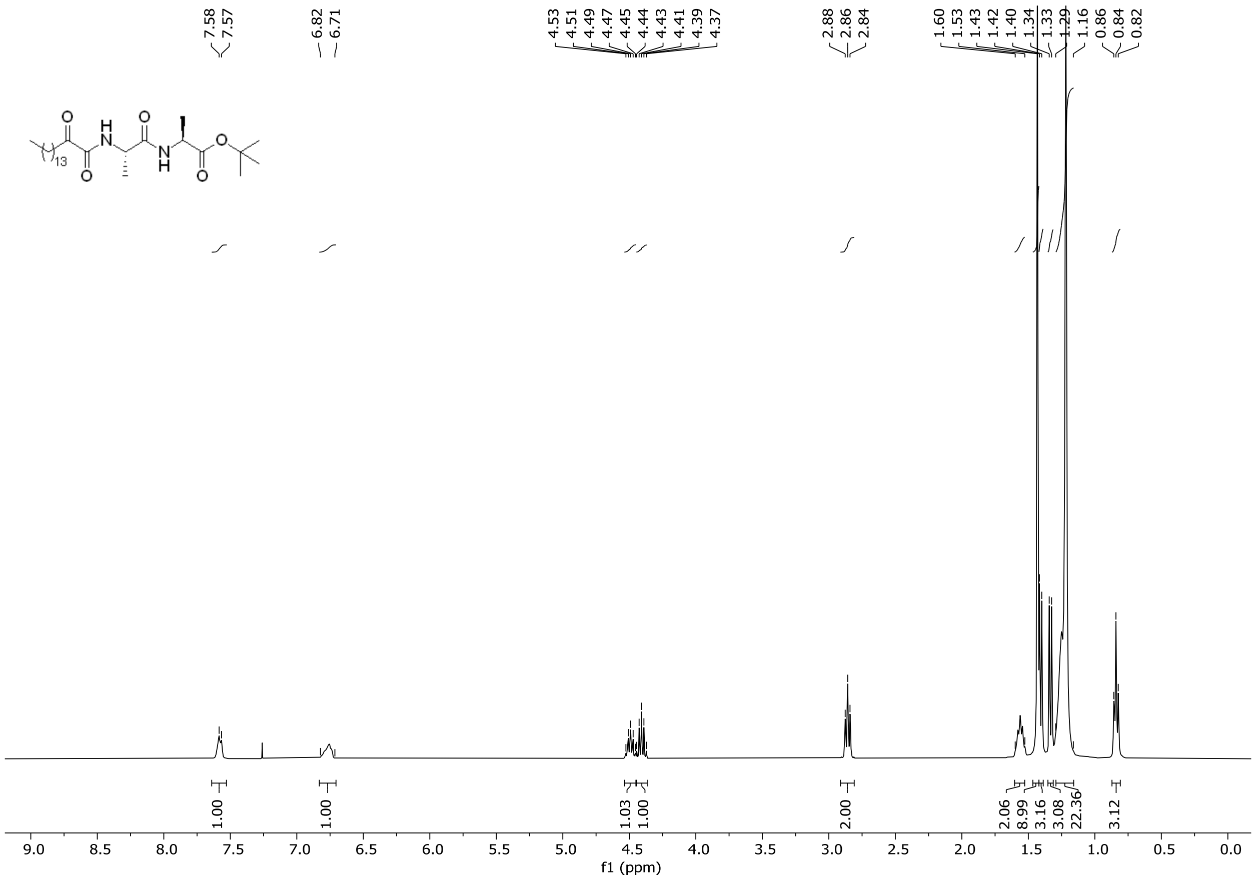

^{1}H NMR spectrum of **7f**.

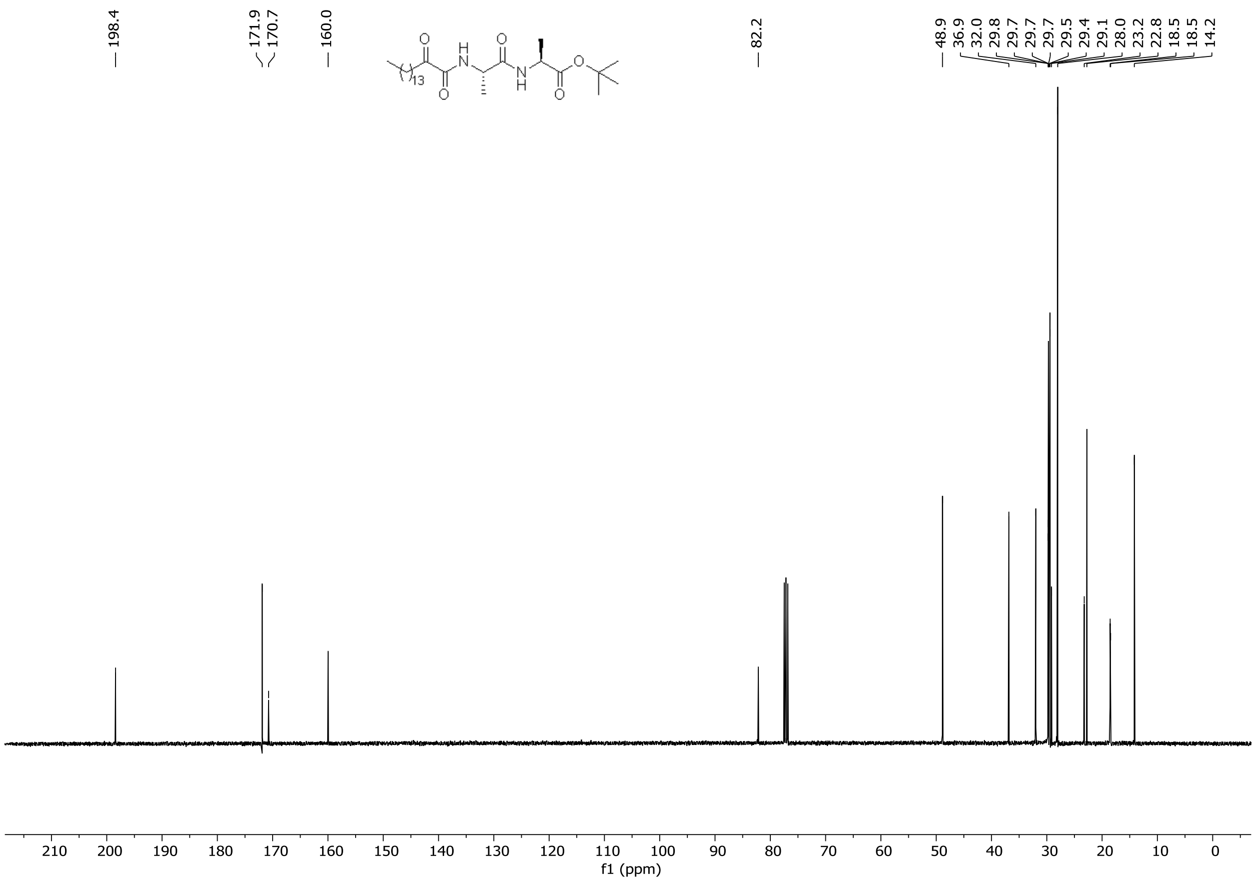

^{13}C NMR spectrum of **7f**.

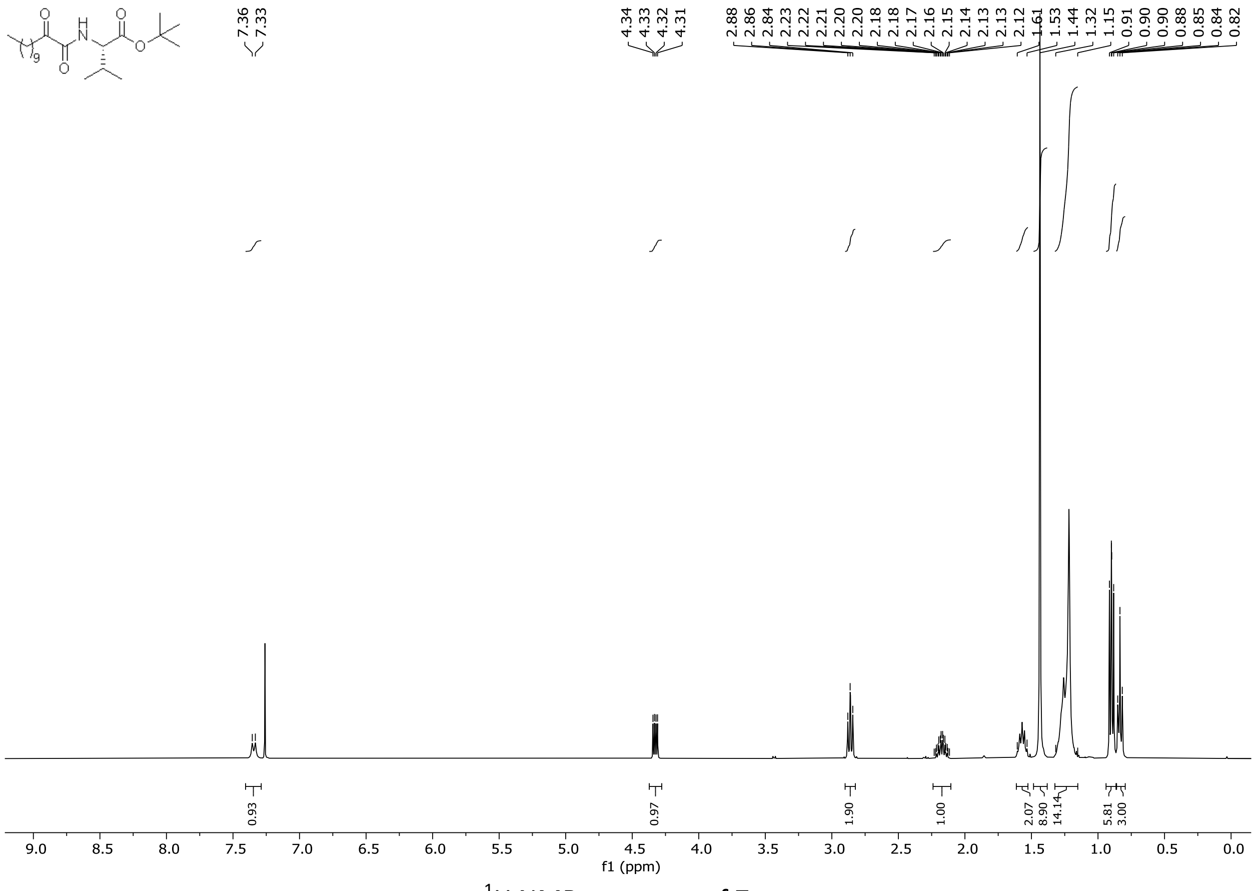

^{1}H NMR spectrum of **7g**.

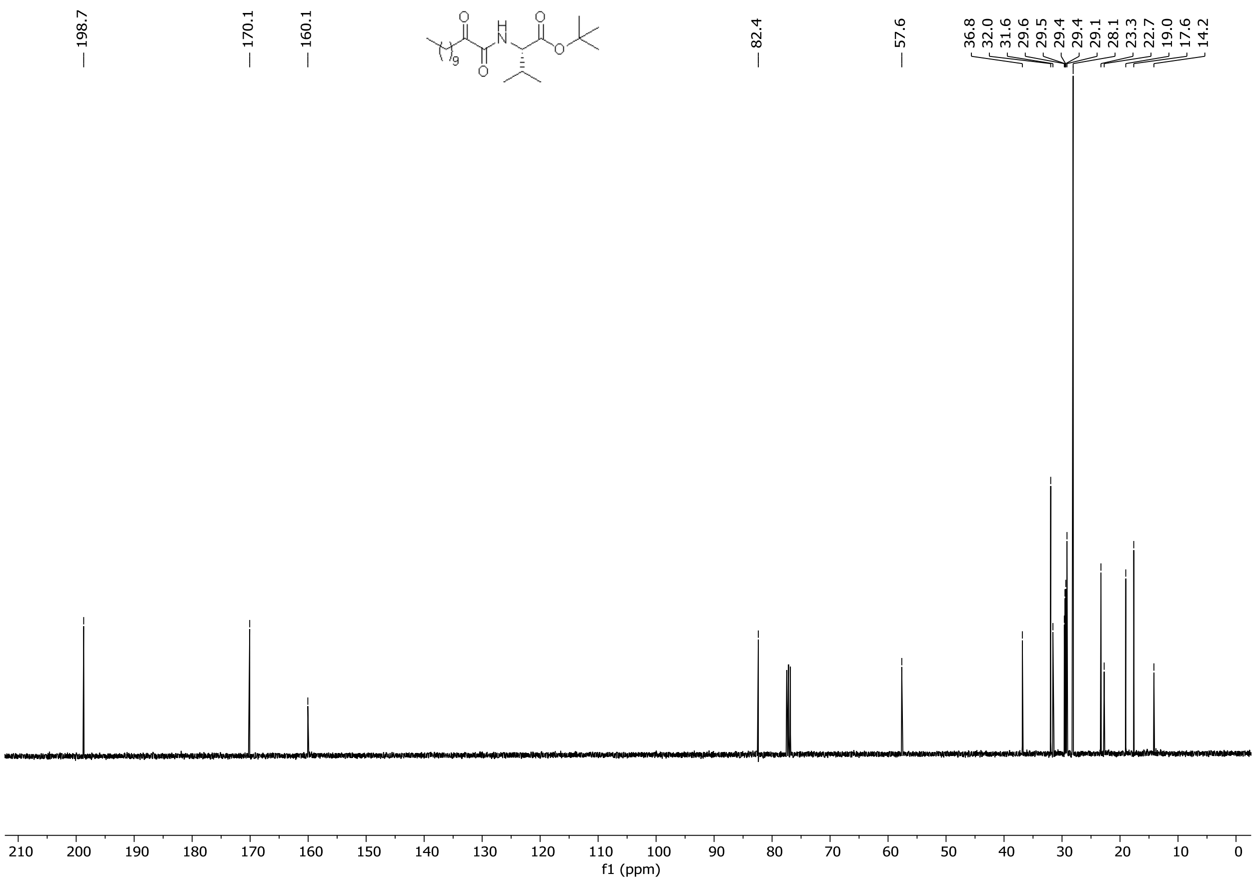

^{13}C NMR spectrum of **7g**.

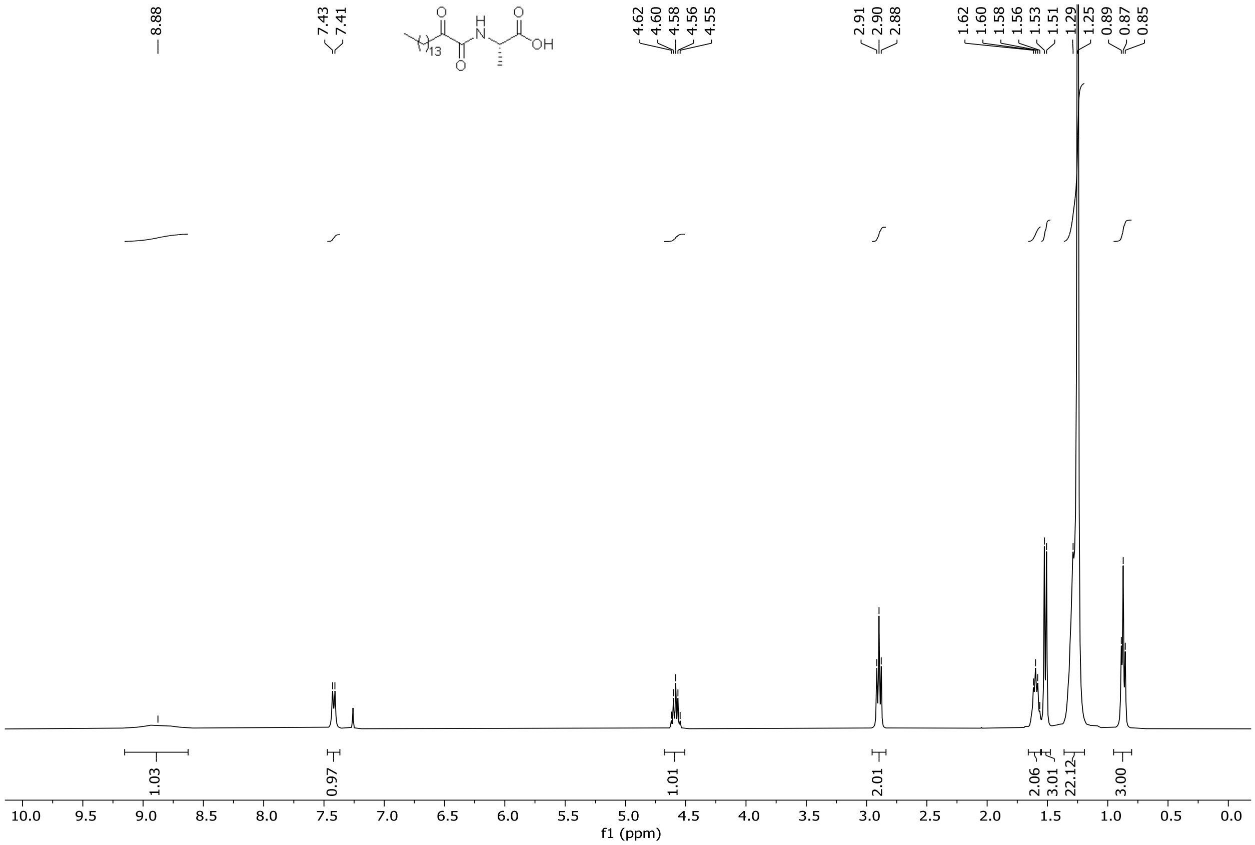

^{1}H NMR spectrum of **8a**.

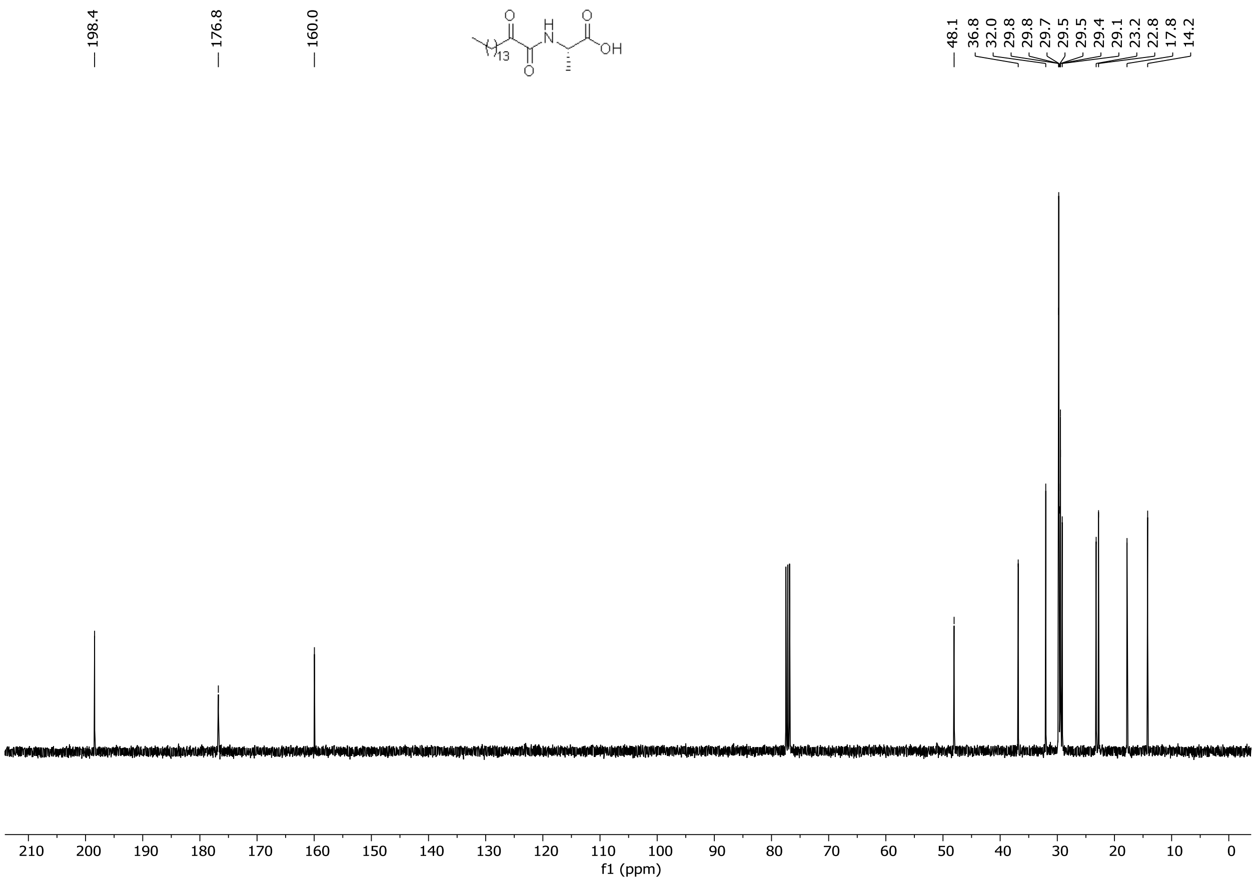

^{13}C NMR spectrum of **8a**.

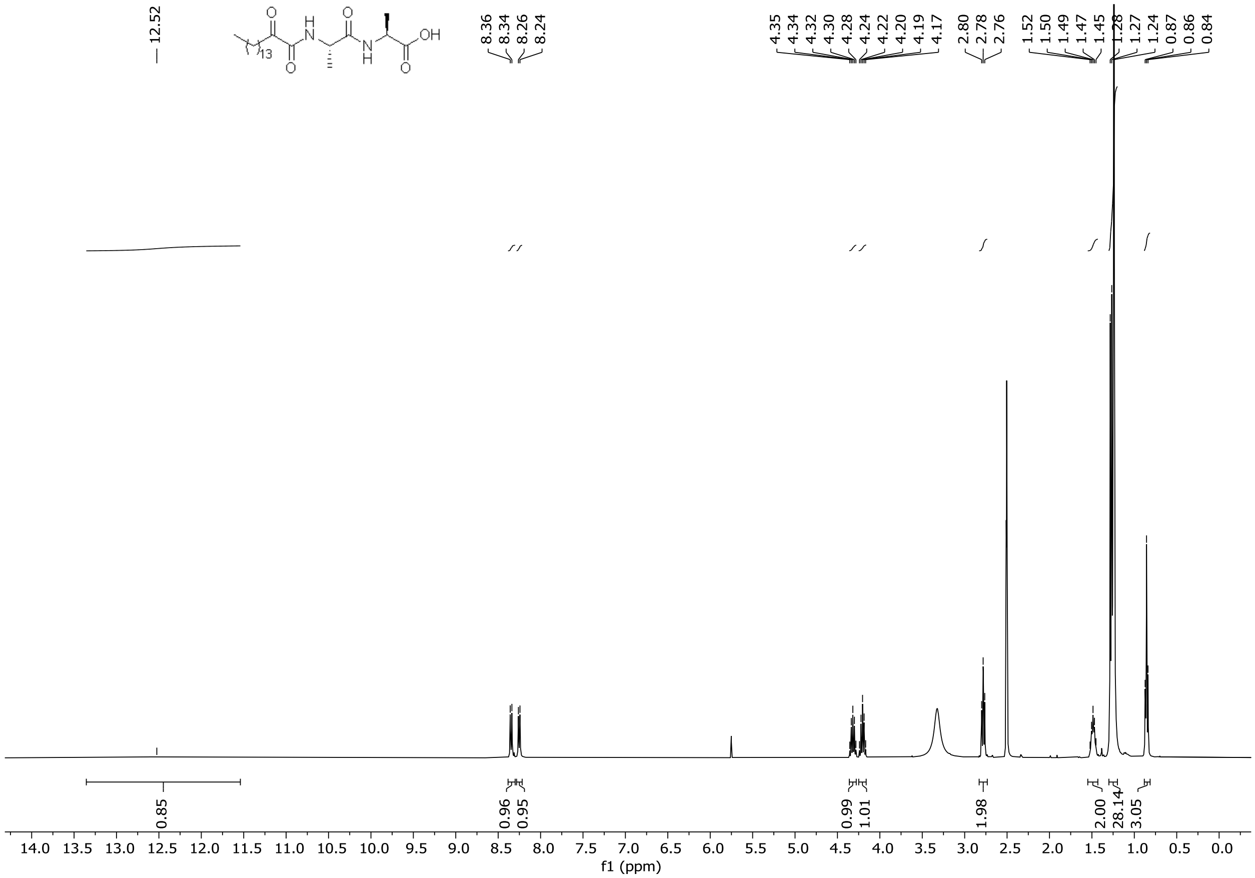

^{1}H NMR spectrum of **8b**.

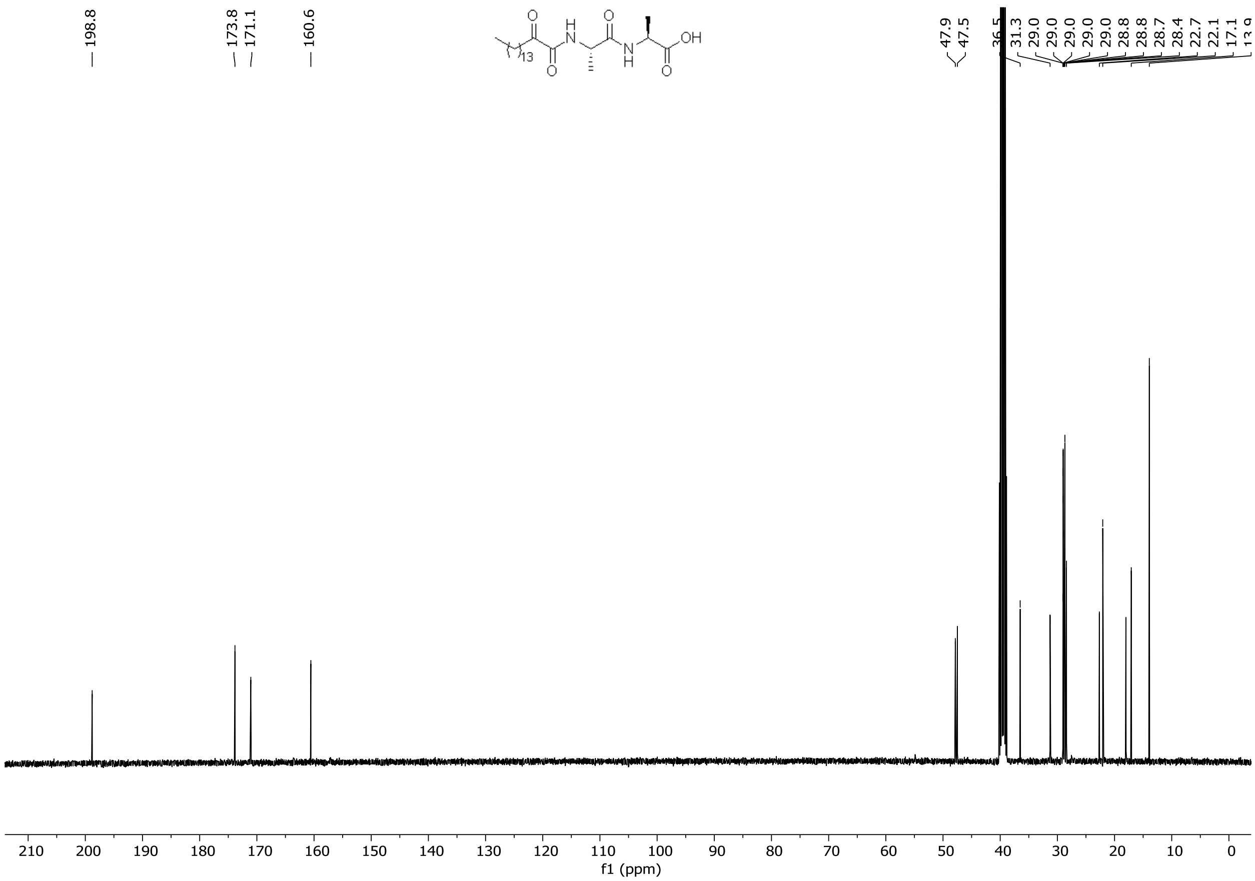

^{13}C NMR spectrum of **8b**.

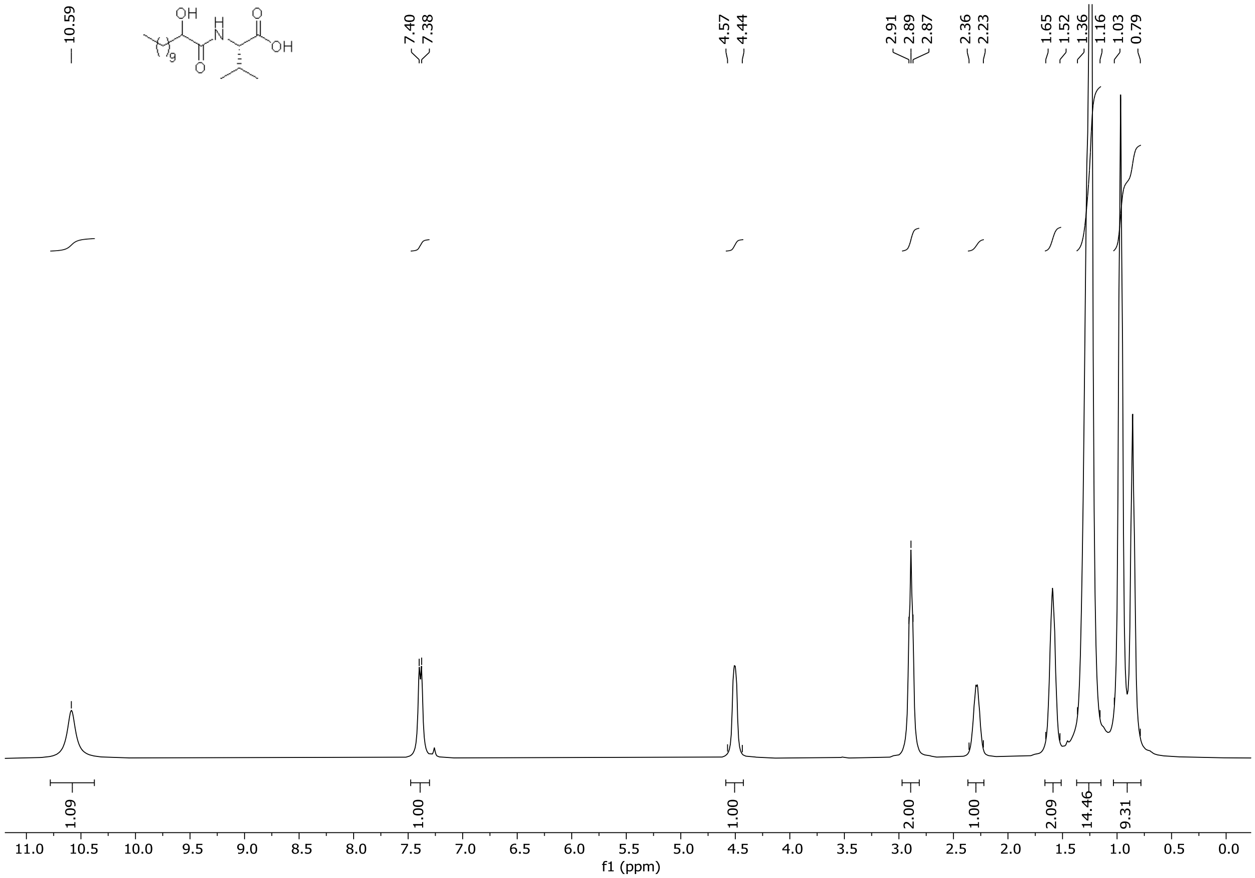

^{1}H NMR spectrum of **8c**.

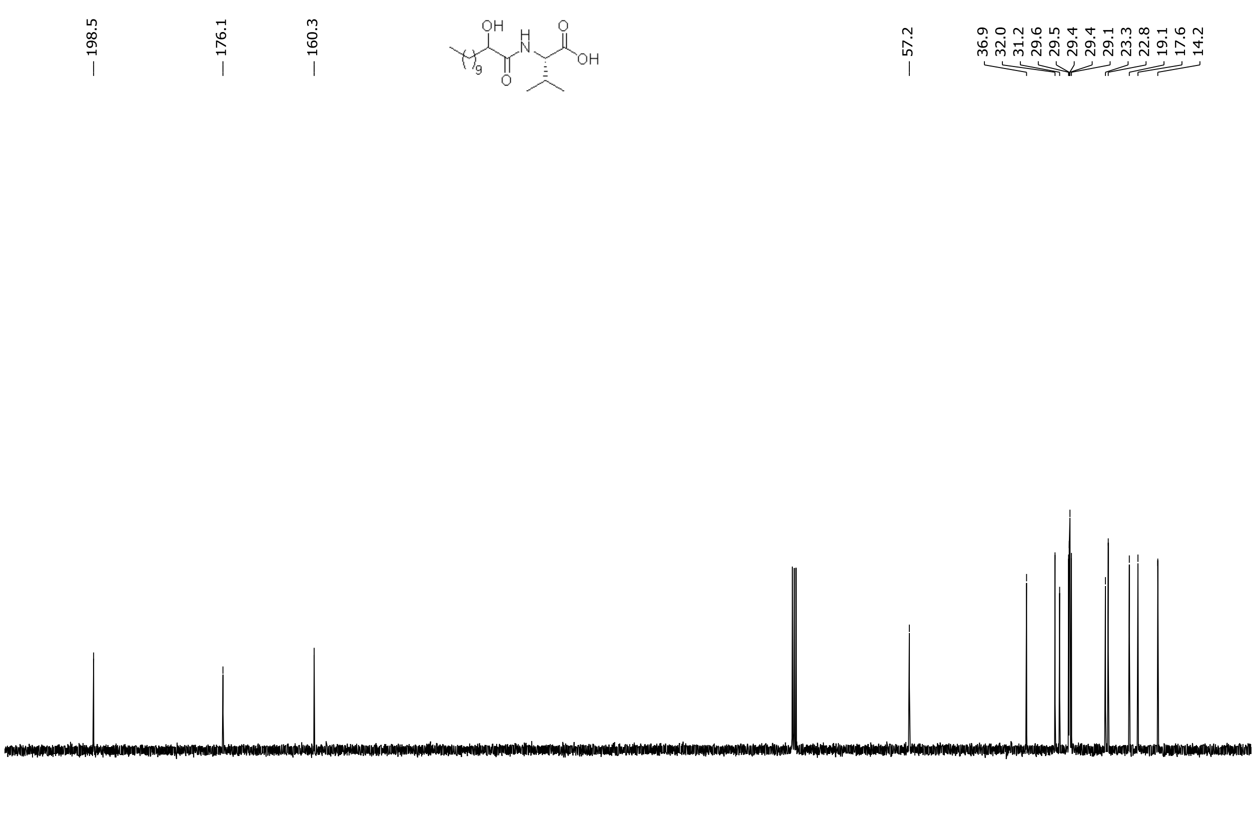

^{13}C NMR spectrum of **8c**.

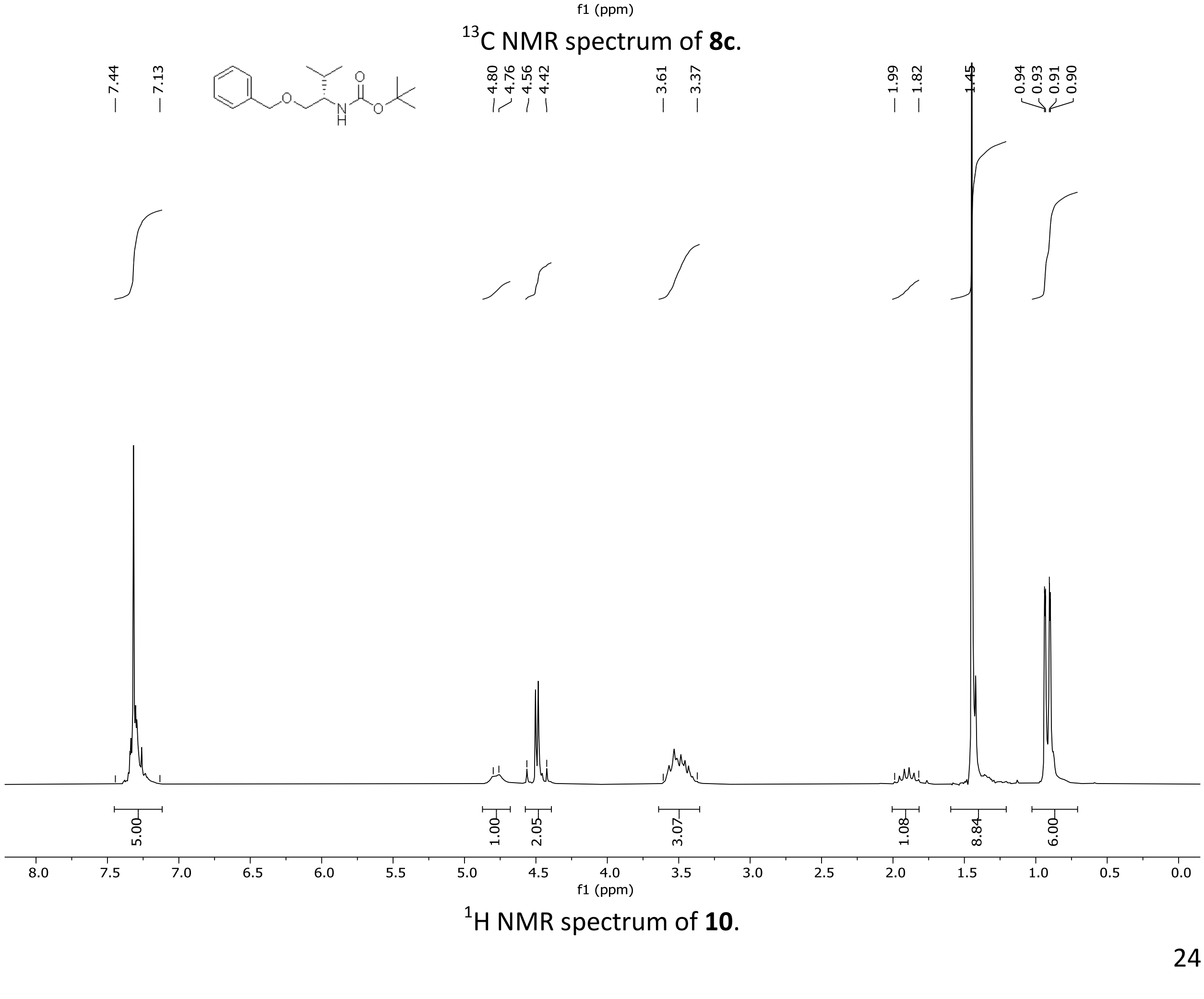

^{1}H NMR spectrum of **10**.

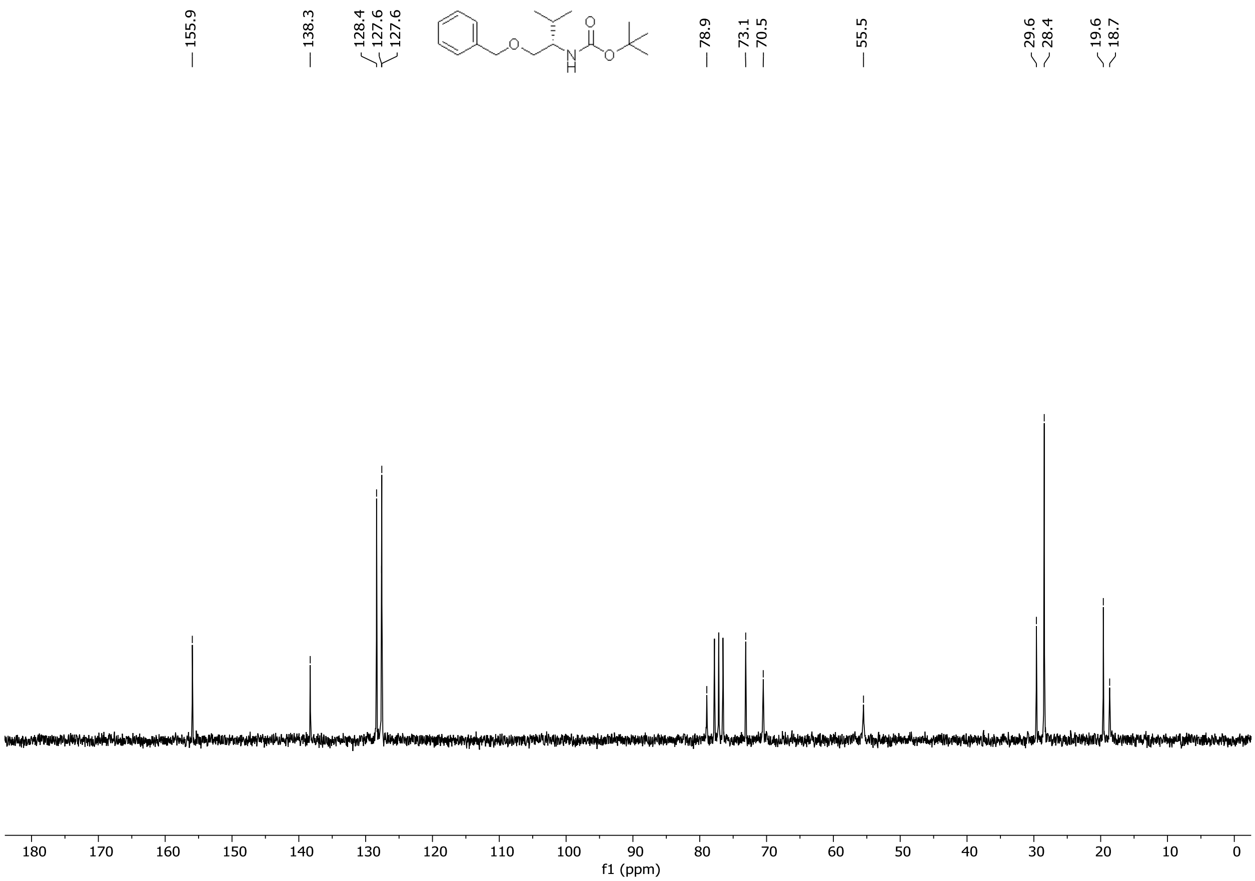

^{13}C NMR spectrum of **10**.
